# Photocatalytic Ammonia Synthesis using Fe-Based MOFs: The Role of Ligand Functionalization

**DOI:** 10.1021/jacs.5c22833

**Published:** 2026-05-19

**Authors:** Jana Bischoff, Cornelia von Baeckmann, Shaghayegh Naghdi, Adrian Ertl, Vasily Vorobyev, Anastasiia Naryshkina, Lakhanlal, Hanspeter Kählig, Laura Kronlachner, Robert T. Woodward, Freddy Kleitz, Andreas Limbeck, Maytal Caspary Toroker, Amanda J. Morris, Dominik Eder

**Affiliations:** † Institute of Materials Chemistry, TU Wien, Getreidemarkt 9, 1060 Vienna, Austria; ‡ Inorganic and Energy Chemistry, Department of Chemistry, Virginia Tech, Blacksburg, Virginia 24060, United States; § Institute of Materials Chemistry and Research, Faculty of Chemistry, 27258 University of Vienna, Währinger Straße 42, Vienna, 1090 Austria; ∥ Department of Materials Science and Engineering, Technion - Israel Institute of Technology, Haifa 3200003, Israel; ⊥ Department of Organic Chemistry, University of Vienna, Währinger Straße 38, 1090 Vienna, Austria; # Institute of Chemical Technologies and Analytics, TU Wien, Getreidemarkt 9, 1060 Vienna, Austria; ∇ Department of Functional Materials and Catalysis, University of Vienna, Währinger Straße 42, 1090 Vienna, Austria; ○ The Nancy and Stephen Grand Technion Energy Program, Technion - Israel Institute of Technology, Haifa 3200003, Israel; ◆ Resnick Sustainability Center for catalysis, Technion − Israel Institute of Technology, Haifa 3200003, Israel

## Abstract

Photocatalytic ammonia (NH_3_) synthesis offers a carbon-neutral alternative to the Haber–Bosch process, which generates 42 million metric tons of CO_2_ equivalent emissions annually. However, solar-to-ammonia conversion with contemporary photocatalysts remains far from practical requirements, and understanding the limiting factors in systems with well-defined active sites is crucial. Here, we show how the μ_3_-oxo-centered trinuclear Fe cluster in MIL-101­(Fe) functions as the catalytic motif for N_2_-to-NH_3_ conversion through combined experimental and computational investigations. Comparative studies with a molecular analogue demonstrate that the cluster is stabilized within the MOF framework, sustaining redox cycling and maintaining high catalytic activity. We systematically functionalized the dicarboxylate ligands of MIL-101­(Fe) with −NH_2_, −Br, −NO_2_, −F, and −CF_3_ to probe how ligand chemistry modulates Fe electron density, N_2_ adsorption capacity, and proton availability, correlating these properties with catalytic performance using spectroscopic and surface characterization techniques alongside time-resolved infrared to assess excited-state lifetimes. F-functionalization optimally balances N_2_ activation, proton availability at Fe active sites, and excited-state lifetimes, boosting NH_3_ production by ∼ 60% relative to unmodified MIL-101­(Fe). This study of ligand-functionalized MIL-101­(Fe) MOFs uncovers the underlying structure-activity relationships and advances design principles for solar-driven NH_3_ synthesis.

## Graphical abstract

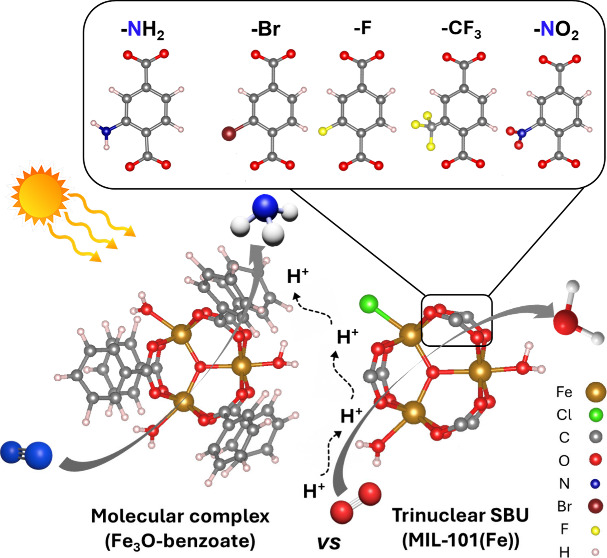

## Introduction

Ammonia (NH_3_) is an essential feedstock in the chemical industry with critical applications in the production of fertilizers, pharmaceuticals, and numerous other nitrogen-containing compounds.^1^ Its potential as a carbon-free hydrogen carrier, with a high hydrogen content, further underscores its importance in sustainable energy solutions.^2,3^ The Haber-Bosch process has enabled large-scale NH_3_ production for over a century but remains highly energy-intensive and dependent on fossil-fuel-derived hydrogen, making it one of the most carbon-intensive industrial processes.^4,5^ Photocatalytic NH_3_ synthesis is envisioned as a promising alternative, requiring only solar energy, nitrogen, and water as a proton source, thereby eliminating the need for external energy input.^6^ However, the activation of the inert N≡N bond^7^ without external energy assistance presents a significant challenge for the catalyst. Additionally, coupling water oxidation to provide protons and N_2_ reduction in a single system, along with the associated multielectron hydrogenation pathway^7^ limit the solar-to-ammonia conversion efficiency.

Metal–organic frameworks (MOFs), as porous materials with well-defined active sites, composed of metal-oxo clusters (secondary building units, SBUs) and organic ligands, have gained increasing interest as catalysts for solar-driven NH_3_ synthesis.^8^ Owing to their modular architecture, MOFs present a tunable well-defined platform to investigate fundamental aspects such as performance limiting factors in photocatalysis. Recently, the use of various metal nodes and fine-tuning of electron density via ligand functionalization to modulate catalytic activity has attracted significant interest.^9–11^ Among the various metal centers explored, Fe stands out due to its biological relevance in nitrogenase, where half-filled d orbitals facilitate N_2_ activation under ambient conditions.^12,13^ Notably, frameworks featuring trinuclear μ_3_-oxo-bridged SBUs (such as MIL-100, MIL-101, and MIL-88B) outperform mononuclear representatives like MIL-53 ^14,15^ and MIL-68,^16^ highlighting the superiority of trinuclear oxo-bridged SBU for efficient NH_3_ synthesis. Several strategies have been explored to improve the photocatalytic performance of MIL-101­(Fe), including defect engineering to increase the accessibility of Fe sites,^14^ creation of mixed-valence Fe­(II)/Fe­(III) SBUs,^17^ enhancement of charge separation via metal doping^18^ as well as incorporation of secondary metals in mixed-metal x/Fe SBUs.^19,20^ Our strategy entails rational modification of organic ligands by introducing substituents that vary in steric demand and electronic character, ranging from electron-donating to strongly electron-withdrawing groups with distinct inductive and resonance effects. This approach enables us to disentangle the multiple effects imposed by ligand functionalization, elucidating how each factor influences photocatalytic performance. As such, our study provides crucial mechanistic insight into this emerging photocatalytic application (Figure 1).

**Figure 1:**
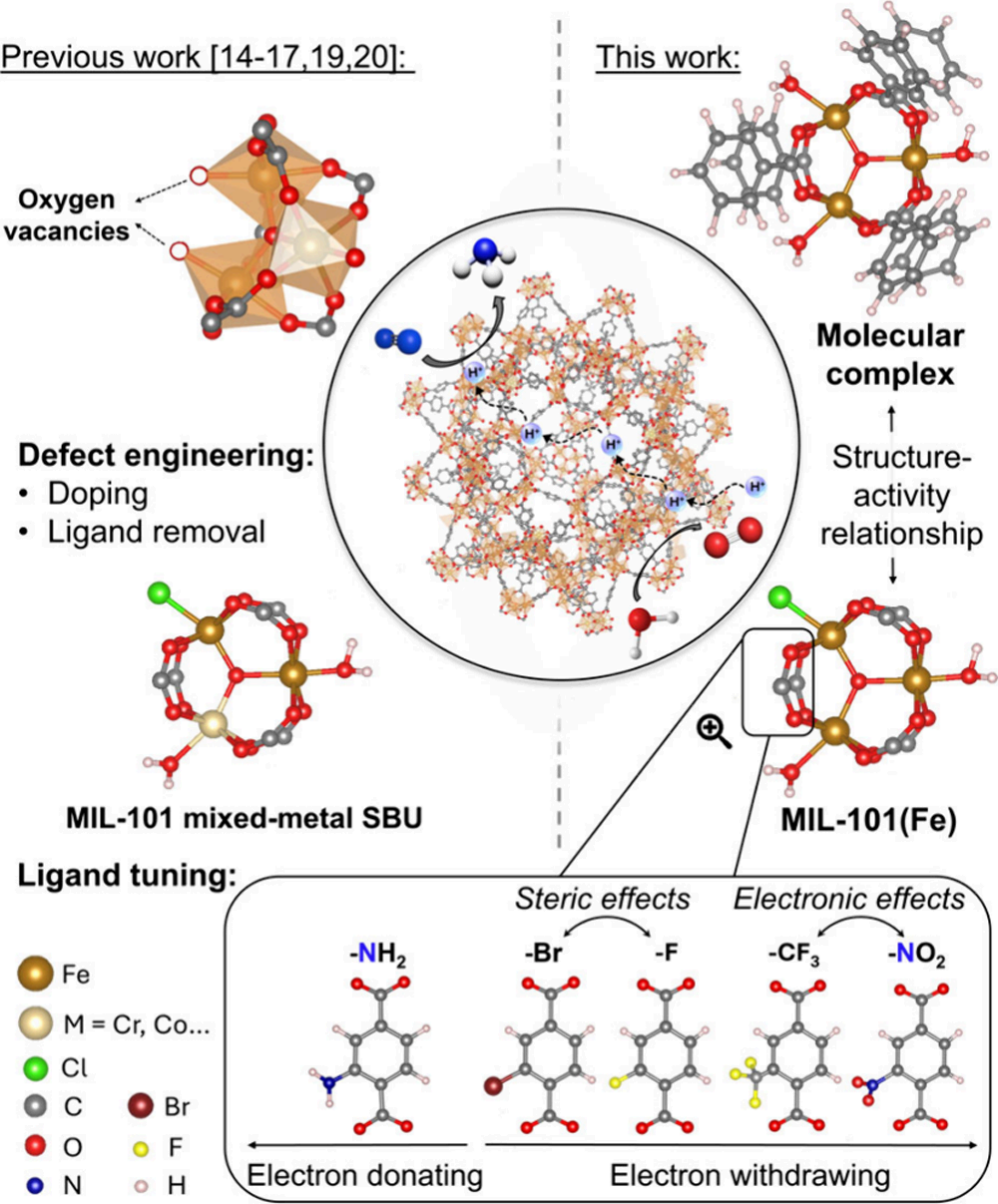
Schematic representation of the present study, illustrating ligand functionalization as a strategy to tune the electronic and steric environment of the MOF SBU.

## Results and Discussion

### Materials Characterization

MIL-101­(Fe) and the μ-oxo-centered trinuclear iron carboxylate complex [Fe_3_O­(PhCOO)_6_(H_2_O)_3_]­ClO_4_ (hereafter referred to as the Fe_3_O-Bz complex) were synthesized following published procedures (details in Methods).^21,22^ Powder X-ray diffraction (PXRD) confirmed the expected crystalline structures of MIL-101­(Fe) without secondary phases or impurities (Figure S1). Scanning electron microscopy (SEM) revealed octahedral MOF particles ranging from several hundred nanometers to the micrometer scale (Figure 2c). Thermogravimetric analysis (TGA) showed structural integrity up to 300 °C, in contrast to the molecular analogue, which undergoes degradation at 165 °C (Figures S3a-S4). Fourier-transform infrared (FTIR) spectroscopy further verified the composition of both MIL-101­(Fe) and the molecular analogue, showing characteristic bidentate bridging coordination, as evidenced by asymmetric and symmetric ν_as_(COO^–^) and ν_sym_(COO^–^) modes at 1591 and 1384 cm^–1^, respectively, for MIL-101­(Fe), with slightly shifted positions observed in the molecular analogue (Figure 2b). X-ray photoelectron spectroscopy (XPS) was employed to probe the surface electronic states (Figures S6a and S7a). The Fe 2p spectrum of MIL-101­(Fe) displays binding energies at 710.2 eV (Fe 2p_3/2_) and 723.3 eV (Fe 2p_1/2_), characteristic of high-spin Fe^3+^ accompanied by satellite shakeup features.^23^ Similar Fe 2p binding energies are observed for the molecular analogue, with slight shifts to higher values (Figures S6g and S7g), attributed to difference in terminal ligands. In the molecular analogue, three aqua ligands coordinate to the Fe_3_(μ_3_-O) core, resulting in a cationic complex, whereas in MIL-101­(Fe), replacement of one aqua ligand by a chloride provides overall charge balance. Nitrogen physisorption analysis at 77 K of MIL-101­(Fe) revealed characteristic Type I isotherms with secondary uptakes at p/p_0_ ≈ 0.1 and p/p_0_ ≈ 0.2, consistent with the presence of two types of microporous windows characteristic of the multiscale porosity of MIL-101­(Fe) (Figure S8a). The corresponding NLDFT pore size distribution shows four distinct pore sizes centered at ∼ 0.6 nm, ∼ 1.1 nm, and ∼ 2.1 nm with an additional shoulder at ∼ 2.7 nm (Figure S8b). These features can be assigned to the internal cavity of the structural supertetrahedron (ST) enclosed by four SBUs, a smaller cage with a pentagonal window, and a larger asymmetric cage with a hexagonal window (structural motifs shown in Figure S10). The apparent Brunauer–Emmett–Teller (BET) surface area was calculated as 2845 m^2^ g^–1^, in good agreement with previous reports.^24,25^ UV–vis diffuse reflectance spectra (DRS) of the Fe_3_O–Bz complex and MIL-101­(Fe) display an intense absorption band centered at ∼ 410 nm, assigned to ligand-to-metal charge transfer (LMCT), together with a weaker absorption feature at longer wavelengths and a broad absorption edge extending to ∼ 600 nm.^26–29^ These features confirm that both the molecular complex and the extended MOF framework exhibit comparable optical responses (Figure S11).

**Figure 2:**
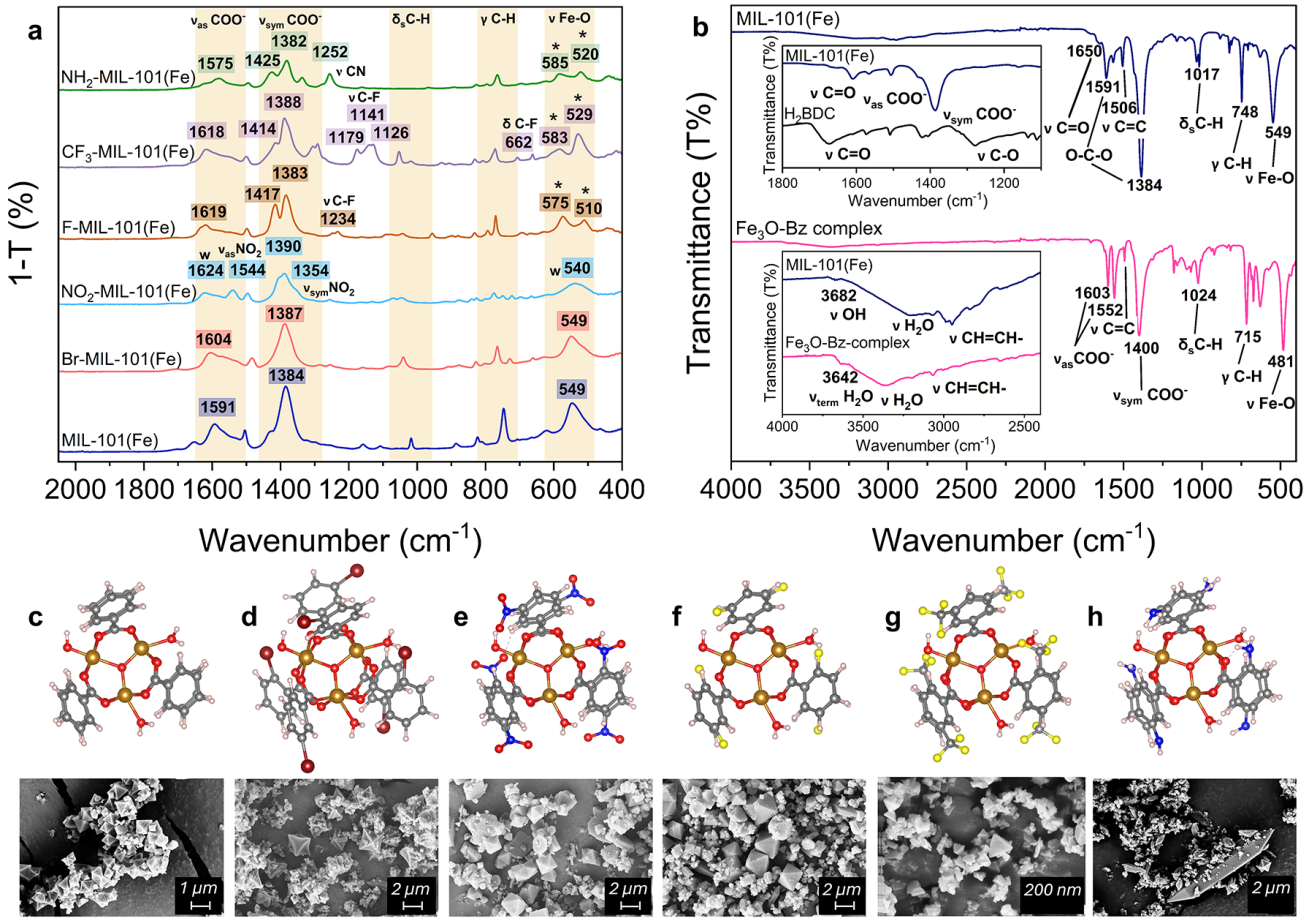
Characterization of single-ligand MIL-101­(Fe) derivatives: (a) FTIR spectra of MIL-101­(Fe) derivatives incorporating BDC, NO_2_–BDC, Br-BDC, F-BDC, NH_2_–BDC, and CF_3_–BDC ligands, showing ligand-specific vibrational features. Hybridization of the NO_2_ group with the aromatic ring broadens the ν_as_(COO^–^) and ν­(Fe–O) bands in NO_2_-MIL-101­(Fe), indicated as wide (w). (b) FTIR spectra of as-prepared MIL-101­(Fe), with an inset comparing the spectrum of the H_2_BDC ligand, and Fe_3_O-Bz complex compared to MIL-101­(Fe), with an enlarged view of the OH and aromatic C–H stretching regions. (c–h) SEM images of the as-prepared MOF samples: (c) MIL-101­(Fe), (d) Br-MIL-101­(Fe), (e) NO_2_-MIL-101­(Fe), (f) F-MIL-101­(Fe), (g) CF_3_-MIL-101­(Fe), and (h) NH_2_-MIL-101­(Fe).

Next, a series of isostructural mixed-ligand MIL-101­(Fe)-based MOFs was synthesized with varying ratios of terephthalic acid (BDC) to amino-terephthalic acid (NH_2_–BDC), denoted as xNH_2_-MIL-101­(Fe), where x represents the percentage of NH_2_–BDC ligand. All compositions were obtained via solvothermal method (details provided in the Methods). The expected MIL-101­(Fe) framework structure was confirmed for all samples by PXRD, SEM, and XPS (Figures S1b; S2, panels f–i; and S6–S7, panels h–k). The actual ligand composition in the mixed-ligand MOFs was quantified by digesting the powders using a 1 M NaOH solution followed by recrystallization with deuterium chloride (DCl) and ^1^H NMR spectroscopy (Figure S14 and Table S1).

Formation of mixed-ligand phases is further supported by FTIR spectra of xNH_2_-MIL-101­(Fe) samples (x = 2%, 5%, 17%, 29%) and the single-ligand NH_2_-MIL-101­(Fe), which exhibit characteristic asymmetric and symmetric N–H stretching bands at 3465 and 3325 cm^–1^. These bands increase in intensity proportionally with the NH_2_–BDC content, accompanied by the appearance of a C–N vibration at 1252 cm^–1^ (Figure S5a), typical of aromatic amines. Notably, a gradual redshift of the ν_as_(COO^–^) peak was observed with increasing NH_2_–BDC ligand content, indicating a weakening of the coordination bond strength (Figure S5b). This shift can be attributed to two factors: (i) the resonance effect of the NH_2_ group with the π system, which increases electron density and reduces carboxylate electrophilicity, thereby diminishing its ionic character toward Fe^3+^ according to the HSAB principle^37^ and (ii) intraframework N–H···O hydrogen bonding between the NH_2_ group and one of the four carboxylate O atoms, which lowers its effective negative charge. Further changes in the coordination environment are evident in the Fe–O stretching region. While non–modified MIL-101­(Fe) shows a band at 549 cm^–1^, the mixed–ligand MOFs display a shoulder at 585 cm^–1^ whose intensity increases with NH_2_–BDC content. This feature supports the presence of asymmetric coordination modes influenced by N–H···O hydrogen bonding. Finally, TGA analysis revealed decomposition temperatures between 270 and 325 °C (Figure S3b), with the onset temperature decreasing progressively with higher NH_2_–BDC content, in line with the FTIR-indicated weakening of the coordination strength. Nitrogen physisorption isotherms for all ligand ratios showed the expected decrease in surface area with increasing NH_2_–BDC content (Figure S8, panels m-n; Table S2). In addition to these structural changes, the amine functional group in the organic ligand acts as a chromophore,^30,31^ leading to bandgap narrowing as observed in DRS measurements (Figure S12).

To exclude effects arising from different ligand ratios, single-ligand, monosubstituted MIL-101­(Fe) variants were synthesized, including NO_2_-, Br-, and NH_2_-MIL-101­(Fe), as well as, to our knowledge, the novel F- and CF_3_-MIL-101­(Fe). The expected framework structure of all variants was confirmed by PXRD, SEM, and XPS analyses (Figures S1a; Figure 2, panels c-h; and Figures S6–S7, panels b-f). PXRD patterns show minor differences in relative peak intensities among functionalized MIL-101­(Fe) derivatives, most notably for CF_3_-MIL-101­(Fe), consistent with slightly smaller particle sizes observed in SEM images (Figure 2g).

The impact of electron-withdrawing groups (EWGs) – Br, NO_2_, F and CF_3_ – on Fe-ligand coordination is reflected in the FTIR spectra of NO_2_-, Br-, F-, and CF_3_-MIL-101­(Fe) (Figure 2a). A blueshift of the ν_as_(COO^–^) peak is observed in all four EWG-MOFs compared to nonfunctionalized MIL-101­(Fe), indicating increased C–O bond polarization and a corresponding strengthening of the coordination bond. The same factors that modulate coordination strength in amine-functionalized MOFs must also be considered here: (i) electron withdrawal through the ligand’s π system and (ii) direct interaction between the functional group and the carboxylate O atom. In the case of EWGs, this direct interaction is expected to be repulsive, due to electrostatic repulsion between the substituent (e.g., F, Br, NO_2_, or CF_3_) and the carboxylate O atoms. In F-MIL-101­(Fe) and CF_3_-MIL-101­(Fe), the ν_sγm_(COO^–^) stretch splits into two bands at 1383 and 1417 cm^–1^ for F-MIL-101­(Fe), and 1388 and 1414 cm^–1^ for CF_3_-MIL-101­(Fe), with the splitting more distinct in F-MIL-101­(Fe). The Fe–O bands are observed at 575 and 510 cm^–1^ for F-MIL-101­(Fe) and 583 and 529 cm^–1^ for CF_3_-MIL-101­(Fe), with the larger Fe–O separation in F-MIL-101­(Fe). The enhanced ν_sγm_(COO^–^) splitting and greater Fe–O band separation in F-MIL-101­(Fe) indicate a more direct interaction between the fluorine substituent and the carboxylate O atoms, whereas the CF_3_ group, being more spatially distant, this effect is weaker. Br-MIL-101­(Fe) does not show pronounced asymmetric features, likely due to weaker repulsion between Br and the carboxylate oxygens. For NO_2_-MIL-101­(Fe), the coordination bands appear broader, reflecting resonance interactions with the BDC π system that enhance electronic delocalization. The ν_as_(COO^–^) band shows the largest blueshift in NO_2_-MIL-101­(Fe), whereas F- and CF_3_-MIL-101­(Fe) exhibit intermediate shifts due to inductive effects, and Br-MIL-101­(Fe), with a weaker inductive character, shows the smallest shift.

To further confirm that ligand functionalization does not distort the Fe_3_O cluster, DFT calculations on the SBU were performed. These results show that interactions between the functional groups (F, CF_3_, NH_2_) and the carboxylate oxygen atoms do not induce significant structural changes. The Fe–O coordination bond lengths and Fe–O–Fe angles, as well as the central O–Fe distances, remain essentially unchanged across all functionalized MOFs, even for the bulkiest CF_3_ variant (Figures S41–S42; Tables S13–S14). Thus, while the IR spectra reflect local polarization and electronic effects, the overall geometry of the Fe_3_O cluster is preserved, in agreement with previous computational studies.^32^

TGA analysis of the single-ligand MIL-101­(Fe)-based MOFs (Figure S3a) shows decomposition temperatures of 262 °C for Br-MIL-101­(Fe), 314 °C for NO_2_-MIL-101­(Fe), 329 °C for F-MIL-101­(Fe), and 339 °C for CF_3_-MIL-101­(Fe). Nitrogen adsorption isotherms revealed Type I behavior for all samples (Figure S8), indicating that the characteristic topology of MIL-101­(Fe) is retained despite ligand functionalization. Pore size distributions reflect the steric influence of the ligands. The smallest pores (∼ 0.61 nm) are only subtly affected, whereas the differential pore volumes of larger cages (1.1, 2.1, and 2.7 nm) decrease with increasing ligand bulk, with the corresponding PSD peaks appearing broader and less resolved, specifically for CF_3_-MIL-101­(Fe). The resulting trend in specific surface areas is MIL-101­(Fe) > F-MIL-101­(Fe) > NH_2_-MIL-101­(Fe) > Br-MIL-101­(Fe) > NO_2_-MIL-101­(Fe) > CF_3_-MIL-101­(Fe), with a similar pattern observed for total pore volumes (Table S2). While ligand substitution modulates pore volumes and surface areas, the intrinsic porosity and framework architecture is maintained. DRS and Tauc plots of monofunctionalized MIL-101­(Fe) variants revealed similar absorption edges across all samples (Figure S13). Consistent with FTIR observations, Br- and F-substituted samples show only minor band gap changes, reflecting the modest influence of their electron-withdrawing (−I) inductive effects. For CF_3_-MIL-101­(Fe), the effect is even less pronounced: although CF_3_ is strongly electron-withdrawing, it primarily exerts a through-bond inductive effect without significant conjugation with the aromatic BDC π system, resulting in minimal modulation of the MOF’s band gap.^33^ In contrast, NO_2_–BDC induces a more pronounced band gap reduction of approximately 0.3 eV compared to nonfunctionalized MIL-101­(Fe), arising from conjugation between the NO_2_ group and the BDC π-system, with NO_2_, acting as an electron acceptor, facilitating electron withdrawal from the aromatic carbon.^33^

To assess the transferability of the ligand functionalization strategy across related frameworks, MIL-88B­(Fe), which shares the same Fe_3_-μ_3_-oxo SBU, and its F-functionalized analogue were synthesized and characterized. PXRD, SEM, and XPS confirmed the expected structure (Figures S1d, S2h,i, S6, and S7l,m). While pristine MIL-88-type materials typically exhibit negligible BET surface area due to a closed-pore structure, ligand functionalization can suppress this contraction, resulting in increased accessible pore size and surface area.^34,35^ This effect is evident as well in our samples, where MIL-88B­(Fe) exhibits a low surface area of 564 m^2^ g^–1^, which increases significantly to 1510 m^2^ g^–1^ for F-MIL-88B­(Fe). Both N_2_ physisorption isotherms correspond to Type I (Figure S9), with the F-functionalized variant showing higher uptake at very low p/p_0_, typical of micropore filling, and consequently a higher micropore area (Table S2). FTIR analysis reflects trends similar to those observed for the MIL-101­(Fe) structure. In the fluorine-functionalized MOF, characteristic band splitting of the ν_sγm_(COO^–^) and Fe–O modes is observed (Figure S5c). TGA reveals that MIL-88B­(Fe) has comparable thermal stability to MIL-101­(Fe), with a degradation onset at 333 °C. The F-functionalized MIL-88B­(Fe) exhibits slightly lower thermal stability, with an onset at 318 °C (Figure S3c). DRS spectra (Figure S14c) reveal the same electronic transitions for both MIL-88B­(Fe) variants, as for MIL-101­(Fe) (∼ 410 nm and ∼ 470 nm), consistent with literature reports.^36,37^ The F-functionalized variant exhibits higher absorbance, which reduces the optical bandgap, as shown in the corresponding Tauc plots (Figure S14d).

### Photocatalytic Activity

The photocatalytic NH_3_ synthesis activity of MIL-101­(Fe) and its functionalized derivatives was evaluated in N_2_-saturated ultrapure water under continuous visible light irradiation (AM 1.5 G, λ ≥ 420 nm, 23 mW cm^–2^) using a 150 W xenon arc lamp. Reactions were carried out in a custom-made quartz reactor equipped with a circulating water jacket to maintain a constant temperature of 15 °C (Figure S15). Prior to the reaction, catalysts were activated at 120 °C under vacuum for 12 h to remove adsorbed gases, particularly nitrogen, which could otherwise lead to false positives. The reaction mixture consisted of 5 mg of catalyst dispersed in HPLC-grade water, which was purged with argon to eliminate contamination from external nitrogen sources. To ensure that the NH_3_ originated from N_2_-to-NH_3_ conversion and not from labile nitrogen-containing compounds typically found in the N_2_ gas stream,^38^ the atmosphere, or the catalyst itself, control experiments were performed: (i) without the catalyst, (ii) without light irradiation, and (iii) without N_2_ input. These control experiments ruled out contributions from external sources and confirmed the detection accuracy (Table S3).

The NH_4_^+^ concentrations, as a proxy for NH_3_ synthesis, in the liquid phase were quantified using the Nessler’s reagent colorimetric method (Figure S16) and qualitatively confirmed via ^1^H NMR spectroscopy of the reaction supernatant (Figure S17). We observed a decrease in NH_4_^+^ production yield with increasing NH_2_–BDC content (Figure 3a). All samples were tested for hydrogen evolution (HER) using gas chromatography, and for hydrazine (N_2_H_4_), nitrate (NO_3_^–^), and nitrite (NO_2_^–^) formation by UV–vis spectrometry to assess selectivity. No side products or N_2_H_4_ intermediates were detected in any case (Figures S18–S20, Table S4). While previous studies have associated amino functionalization with enhanced photocatalytic activity due to increased light absorption,^39,40,41^ our results reveal the opposite trend - despite a gradual bandgap reduction confirmed by Tauc plots (Figure S12b), consistent with observations in NH_2_-MIL-125­(Ti).^42^ We hypothesize that in NH_2_-MIL-101­(Fe), photoexcitation-induced LMCT may cause holes to accumulate at the NH_2_ nitrogen, which inhibits water oxidation and reduces the availability of protons, thereby slowing NH_4_^+^ formation. This interpretation is supported by projected density of states (PDOS) analysis, which shows that the states just below the Fermi level in the amino-functionalized MOF originate predominantly from the nitrogen atom (Figure S26e). This suggests that these orbitals are where holes tend to localize after photoexcitation. Our hypothesis is supported by linear sweep voltammetry (LSV) under visible-light irradiation (AM 1.5 G, λ ≥ 420 nm, 23 mW cm^–2^, 150 W Xe lamp), which shows that NH_2_-MIL-101­(Fe) exhibits a positive anodic onset and reduced photocurrent compared to pristine MIL-101­(Fe) (Figures S21 and S22). This observation is consistent with transient photocurrent measurements under chopped light, where the NH_2_-functionalized MOF generates lower photocurrents.

**Figure 3:**
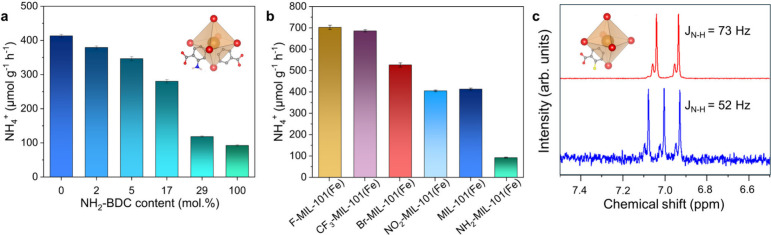
Photocatalytic NH_4_^+^ production rates for (a) mixed-ligand xNH_2_-MIL-101­(Fe) MOFs and (b) single-ligand monofunctionalized MIL-101­(Fe) variants (F-, CF_3_-, Br-, NO_2_-, NH_2_-, and unmodified MIL-101­(Fe)). Error bars represent standard deviation from triplicate measurements. (c) ^1^H NMR spectra of solution after 1 h photocatalysis using F-MIL-101­(Fe) in ^14^N_2_ and ^15^N_2_ atmosphere.

While these results provide a clear trend, additional factors related to ligand functionalization should be considered, including changes in Fe Lewis acidity (influencing N_2_ activation), variations in excited-state lifetime, and functionalization-induced changes in pore structure that affect reactant accessibility as well as proton transfer due to altered surface charge.

To investigate the influence of EWGs, we compared the photocatalytic NH_4_^+^ synthesis performance of single-ligand MIL-101­(Fe) variants. NO_2_-MIL-101­(Fe) (404 μmol g^–1^ h^–1^) exhibited activity nearly identical to unmodified MIL-101­(Fe) (413 μmol g^–1^ h^–1^), whereas Br-MIL-101­(Fe) showed a substantial increase (526 μmol g^–1^ h^–1^). The highest activity was observed for F-MIL-101­(Fe), which generated 702 μmol g^–1^ h^–1^, closely followed by CF_3_-MIL-101­(Fe) at 686 μmol g^–1^ h^–1^ (Figure 3b). Isotope-labeling experiments using F-MIL-101­(Fe) confirmed that NH_4_^+^ originates from introduced N_2_. Experiments under ^14^N_2_ atmosphere, ^1^H NMR displayed the characteristic ^14^NH_4_^+^ triplet (^J^N–H = 52 Hz), whereas experiments under ^15^N_2_ showed two doublets at 6.9 ppm (^J^N–H = 73 Hz), confirming the formation of ^15^NH_4_^+^ (Figure 3c). For all tested single-ligand MOFs, no other reaction products were detected, confirming the selectivity of N_2_-to-NH_3_ conversion (Figures S18–S20). Interestingly, the observed activity trend does not correlate with the MOFs’ optical absorption or electron-withdrawing strength. Although NO_2_-MIL-101­(Fe) exhibits the largest bandgap modulation, it does not show the highest catalytic activity. In contrast, Br-, F-, and CF_3_-MIL-101­(Fe) display only minor changes in their optical properties yet outperform NO_2_-MIL-101­(Fe) in NH_4_^+^ production.

### Structure–Activity Relationship

The electronic impact of EWGs on the Fe centers was examined via Fe 2p XPS analysis. Relative to unmodified MIL-101­(Fe), the Fe 2p_3/2_ binding energies shift positively by 0.3 eV (Br), 0.3 eV (F), 0.4 eV (NO_2_), and 0.6 eV for CF_3_-MIL-101­(Fe), reflecting reduced electron density at Fe and a more ionic Fe-ligand bond. These shifts align with FTIR evidence of coordination modulation, showing that electron-withdrawing ligands deplete electron density at Fe, with the strongest effect observed for CF_3_ substitution.

By contrast, the electron-donating NH_2_ group does not induce a measurable shift (Figure S7, panels b-e). The impact of this modified Fe electron density on N_2_ adsorption was further examined via temperature-programmed desorption (N_2_-TPD) coupled with mass spectrometry (MS) to identify the desorption signals. Prior to measurements, samples were activated, saturated with N_2_, and purged with He to remove excess N_2_, including physisorbed molecules (details in the Methods). As shown in Figure S23, pristine MIL-101­(Fe) and NH_2_-MIL-101­(Fe) exhibit desorption peaks at ∼ 150–60 °C, whereas samples functionalized with EWGs show higher desorption temperatures of ∼ 180 °C, ∼ 194 °C for CF_3_-MIL-101­(Fe), reaching 205 °C for NO_2_-MIL-101­(Fe).

Although several reports have attributed the high-temperature desorption peak (∼ 220 °C) to chemisorbed N_2_,^43,44^ our MS analysis (*m*/*z* = 44) shows that the signal instead corresponds to CO_2_ desorption. This may originate from ligand decarboxylation or from removal of strongly chemisorbed CO_2_. This CO_2_ peak is distinct in the amino-functionalized sample, consistent with the reported high chemisorption due to hydrogen bonding between the oxygen electron pair in CO_2_ and the hydrogen atoms of the NH_2_ group.^45,46^ In contrast, Br-, F-, NO_2_-, and pristine MIL-101­(Fe) exhibit CO_2_ signals that partially overlap with the N_2_ chemisorption peak. Nevertheless, PXRD and FTIR (Figure S21) reveal only minor differences before and after TPD, indicating that ligand decomposition contributes negligibly to the CO_2_ signal. The N_2_ uptake capacities are comparable among the samples, with values of 0.27 mmol g^–1^ for CF_3_-MIL-101­(Fe), 0.28 mmol g^–1^ for Br-MIL-101­(Fe), 0.29 mmol g^–1^ for F-MIL-101­(Fe) (Figure 4d), 0.34 mmol g^–1^ for pristine MIL-101­(Fe), and 0.36 mmol g^–1^ for NO_2_-MIL-101­(Fe). For Br-, F- and CF_3_-MIL-101­(Fe), this correlates with their specific surface areas, while NO_2_-MIL-101­(Fe) shows a notably higher uptake despite its lower surface area due to the bulky substituent. Importantly, the higher desorption temperatures of EWG-functionalized samples indicate stronger N_2_ binding, consistent with FTIR evidence and the electron-withdrawing effects observed in XPS. Noteworthy differences are observed in the desorption peak shapes. NO_2_-MIL-101­(Fe) shows a very broad peak, reflecting both the moderate steric bulk of the NO_2_ group (van der Waals radius 200–210 pm)^47^ and stronger interactions between N_2_ molecules and the framework during desorption, likely due to the polar and electron-withdrawing character of NO_2_. In contrast, CF_3_-MIL-101­(Fe), despite being bulkier (van der Waals radius ∼ 220 pm),^47^ exhibits a narrower peak, consistent with weaker interactions of N_2_ with the CF_3_ group, which is more hydrophobic and oriented away from the pore cavity. Br-MIL-101­(Fe) (185 pm), F-MIL-101­(Fe) (147 pm), and unfunctionalized MIL-101­(Fe) show quite similar, moderately broad peaks, indicating intermediate interactions. These observations suggest that desorption behavior is influenced by steric hindrance imposed by the functional groups, emphasizing the importance of considering pore structure and dangling functional groups pointing inside the pore channels. On the other hand, MIL-88B­(Fe) and F-MIL-88B­(Fe) exhibit very low N_2_ adsorption capacity (∼ 0.014–0.017 mmol g^–1^, Figure S23g,h) due to closed pores and structural collapse under measurement conditions, as evident in postmeasurement PXRD patterns (Figure S24c), However, this does not reflect their behavior in aqueous photocatalysis, where the frameworks adopt an open-pore configuration allowing full reactant accessibility.^48^ Accordingly, their photocatalytic NH_3_ production reaches 114 and 191 μmol g^–1^ for MIL-88B­(Fe) and F-MIL-88B­(Fe), respectively (Figure S27b). No side products or N_2_H_4_ intermediates were detected for either sample (Figures S18–S20; Table S4).

**Figure 4:**
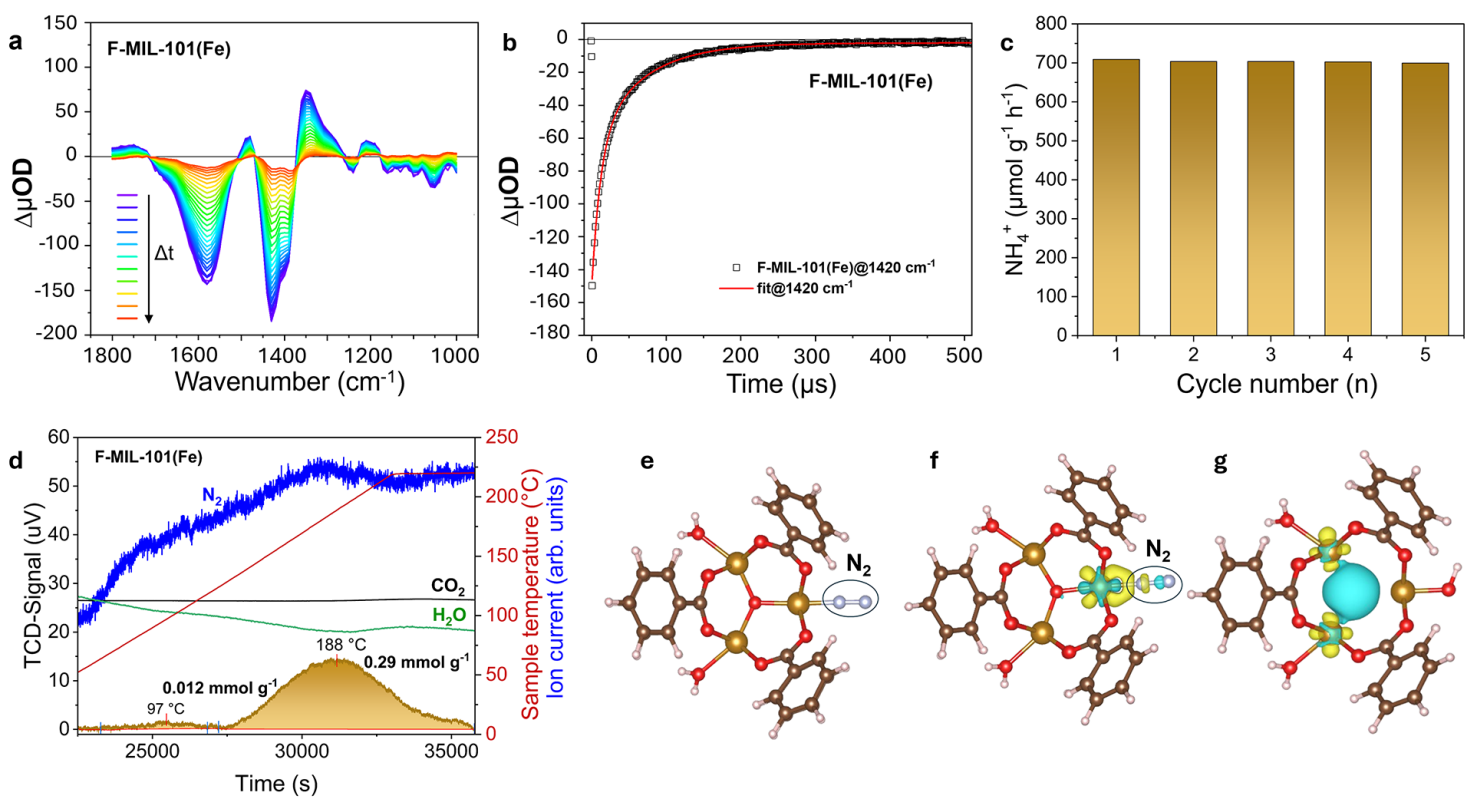
(a) TRIR difference absorption spectra of F-MIL-101­(Fe) at varied time delays (140 ns–114 μs) upon 532 nm excitation. Spectra are color-coded from purple (early) to yellow (late), illustrating temporal evolution. (b) Corresponding decay kinetics with biexponential fits; at 1420 cm^–1^ the photoexcited F-MIL-101­(Fe) exhibits biexponential decay with time constants of 11.8 ± 0.2 μs and 64.3 ± 0.9 μs. (c) NH_4_^+^ yield obtained from photocatalytic cycling experiments over F-MIL-101­(Fe). (d) N_2_-TPD-MS profile of F-MIL-101­(Fe), showing chemisorbed N_2_ desorption features. (e) DFT analysis on a cluster model of the MIL-101­(Fe) SBU: end-on N_2_ adsorption at an exposed Fe site. (f) Charge-density difference map illustrating N≡N bond polarization upon adsorption (cyan: charge depletion; yellow: charge accumulation). (g) Charge-density difference map showing electron distribution after removal of the μ_3_-O atom.

Essentially, ligand functionalization tunes both the coordination strength and the electron density on Fe, both of which are critical for enabling N_2_ activation. In NO_2_-MIL-101­(Fe), despite the electron-withdrawing properties of the NO_2_ group that reduces electron density on Fe (as observed in XPS), the photocatalytic activity is similar to that of unfunctionalized MIL-101­(Fe). This behavior likely arises from the strong electronegativity of the NO_2_ group, which withdraws electron density from the coordinating oxygen atoms and serves as a sink for photogenerated holes, which is likely stabilized via resonance delocalization through the NO_2_ aromatic π system. Similar to NH_2_-MIL-101­(Fe), this effect is supported by linear sweep voltammetry (LSV), which shows a higher water oxidation onset potential for NO_2_-MIL-101­(Fe) compared to unfunctionalized MIL-101­(Fe) (Figure S21). Transient photocurrent measurements under chopped light further confirm slower hole extraction, with NO_2_-MIL-101­(Fe) exhibiting lower overall photocurrents than the other materials (Figure S22).

In contrast, other EWGs MOFs, such as Br-, F-, and CF_3_-MIL-101­(Fe), display opposite trends in LSV and photocurrent measurements, with lower onset potentials and higher photocurrents even compared to unfunctionalized MIL-101­(Fe). These observations indicate that these variants achieve more efficient hole extraction and, correspondingly, higher photocatalytic performance, highlighting the importance of charge extraction.

While the bulk reaction medium is acidic (pH ∼ 3, Table S4), providing abundant protons, local surface charge effects modulate their accessibility at the Fe centers. The markedly lower ζ-potential of NO_2_-MIL-101­(Fe) (+6 mV vs +27 mV for MIL-101­(Fe), Table S5) indicates an altered particle surface charge, which likely reduces the local availability of protons near the catalytic sites compared to the unfunctionalized MOF. In contrast, the slightly higher ζ-potential of NH_2_-MIL-101­(Fe) (28 mV vs 27 mV) suggests that protonation occurs at mainly the amino groups of the ligand, rather than at the Fe centers, illustrating how surface functionality can modulate proton distribution. Combined with its intrinsically poor N_2_ chemisorption capacity and hole trapping, this renders NH_2_-MIL-101­(Fe) the least active sample for NH_4_^+^ formation. In contrast, halogenated ligands exhibit the opposite behavior. Although the bulk pH remains constant (pH ∼ 3), the surface ζ-potentials of Br-, F-, and CF_3_-MIL-101­(Fe) (+17, + 19, and +19 mV, respectively) are higher than that of NO_2_-MIL-101­(Fe) (+6 mV), suggesting enhanced retention of surface protons. Therefore, the electronegative halogen atoms repel protons away from the ligand, directing them toward the Fe centers. This explains why CF_3_-MIL-101­(Fe) and F-MIL-101­(Fe) exhibit similar activity trends, despite the CF_3_ variant having a lower surface area, and why the effect is slightly weaker for Br-MIL-101­(Fe), consistent with the observed trend: F-MIL-101­(Fe) > CF_3_-MIL-101­(Fe) > Br-MIL-101­(Fe).

Protonation effects were further evaluated through pH-dependent activity tests. Upon increasing the initial pH of the dispersion to 7, both F-MIL-101­(Fe) and NO_2_-MIL-101­(Fe) exhibit a clear decrease in activity, retaining approximately 60% and 70% of their initial NH_4_^+^ yields, respectively. At pH 8, the activity drops further to ∼ 25% and ∼ 20% of the initial values, and at pH 10 only a baseline activity of ∼ 14 μmol g^–1^ h^–1^ is observed for both materials (Figure S25a and b). This gradual decline reflects the reduced availability of H^+^, which limits proton-coupled electron transfer steps essential for ammonia formation. In contrast, NH_2_-MIL-101­(Fe) exhibits a slight increase in activity at pH 7 compared to native dispersion pH of ∼ 3. This behavior can be attributed to partial deprotonation of surface – NH_3_^+^ groups, which reduces proton trapping and improves access to active sites. At higher pH (pH 8), the activity decreases again, indicating a transition to a regime where limited H^+^ availability becomes the dominant factor. This is consistent with the trends observed for F- and NO_2_-functionalized samples, which do not undergo significant functional group protonation and therefore show a continuous decline in activity with increasing pH.

Interactions between the carboxylate and the functional groups in the ligand tune the electronic environment of the SBU, impacting the lifetimes of the photoexcited states. In fact, reversible changes in coordination configuration can transiently expose delocalized Fe centers. This phenomenon, known as photoinduced dynamic ligation (PDL), was first reported in MIL-101­(Fe)^21^ and later observed in its NH_2_- and NO_2_-functionalized derivatives.^49^ In this study, TRIR measurements further explored the occurrence of PDL in F-MIL-101­(Fe) and Br-MIL-101­(Fe). Specifically, the presence of excited state absorption (ESA) features at the far red and blue side of the measured spectral range (peaks around 1200 and 1750 cm^–1^) reflected the formation of C–O and C = O bonds in the excited states (Figure 4a-b and Figure S26), indicating a transition from a symmetric ground state coordination to an asymmetric monodentate configuration upon photoexcitation, accompanied by the transient generation of delocalized Fe^2+^ states. Moreover, both F-MIL-101­(Fe) and Br-MIL-101­(Fe) exhibited two-component decay behavior (11.8 ± 0.2; 64.3 ± 0.9 μs and 12.0 ± 0.3; 62.6 ± 1.0 μs, respectively), similar to that observed in the NH_2_-MIL-101­(Fe) and NO_2_-MIL-101­(Fe) systems. According to a previous report,^49^ the longer-lived component corresponds to excited states associated with the functional groups, indicating repulsive interactions between the negatively charged halogen and nearby oxygen atoms of the carboxylate group. The weighted average lifetimes of the photoexcited MOFs decreased in the order NO_2_-MIL-101­(Fe) > F-MIL-101­(Fe) > Br-MIL-101­(Fe) > MIL-101­(Fe) > NH_2_-MIL-101­(Fe) (Table S7). This trend is partially consistent with the observed NH_4_^+^ production, with NO_2_-MIL-101­(Fe) as an exception, highlighting that catalytic performance is determined by multiple factors. Efficient N_2_ hydrogenation relies on the interplay between charge-carrier dynamics and local proton availability, emphasizing that prolonged excited-state lifetimes alone are insufficient to achieve optimal activity.

To investigate how the redox cycle is sustained within the oxo-bridged trinuclear SBU, we tested a μ-oxo-centered trinuclear carboxylate complex, as a molecular analogue of the MIL-101­(Fe) SBU. Its NH_4_^+^ synthesis performance was evaluated under identical conditions, with Fe molarity matched. The molecular complex produced 126 μmol g^–1^ h^–1^ of NH_4_^+^ (Figure S27a), nearly half of that observed with MIL-101­(Fe), likely due to its lower structural stability. While the molecular complex loses crystallinity after reaction (Figure S35c), MIL-101­(Fe) retains its crystalline framework, with initial activity observed even within the first 5 min and higher conversion rates over 1 h (Figures S28–S29). This underscores the advantage of the heterogeneous MOF approach in stabilizing the oxo-bridged SBU and sustaining NH_4_^+^ formation.

DFT calculations were performed to elucidate the N_2_ activation mechanism. Adsorption energy calculations indicate that N_2_ binds end-on at exposed Fe sites (Figure 4e, and Figures S31–32), polarizing the N≡N bond (Figure 4f). This is reflected in the PDOS, where the N_2_ 1π orbital shifts toward the Fermi level, accompanied by changes in the bonding 2σ orbital (Figure S33). These PDOS changes align with established N_2_ activation mechanisms, where σ-donation from N_2_ to Fe and π-backdonation from Fe d-orbitals to N_2_ π* orbitals facilitate activation.^50,51^ While this explains N_2_ activation on exposed Fe sites, the unique catalytic motif capable of performing both water oxidation and N_2_ reduction remains elusive. Achieving a balance between electron acceptance and donation at a single active site is a known challenge for monometallic catalysts, as it involves a trade-off between these interactions.^52,53^

Observing the MIL-101­(Fe) SBU structure and the fact that the molecular complex is also active, albeit with reduced stability, we propose that the central oxygen is crucial for catalytic function. The Fe^3+^ centers within the SBU behave as hard Lewis acid,^54^ accepting electron density from N_2_, consistent with the N_2_ chemisorption observed under dark conditions in N_2_-TPD experiments as the initial step of N_2_ activation. The bridging μ_3_-O atom withdraws electron density from the Fe centers, increasing their positive character and enhancing N_2_ polarization. This is supported by Bader charge analysis of MIL-101­(Fe) and the central-oxygen-removed MIL-101­(Fe), which indicates that removal of the μ_3_-O atom leads to an increase in electronic charge on the nearby Fe centers (see Table S9 for Bader charge data), consistent with the charge redistribution (Figure 4g), resulting in unfavorable N_2_ adsorption. DFT calculations further corroborate this behavior, showing that N_2_ adsorption is highly endergonic (+2.05 eV) in the absence of the central oxygen but becomes favorable (−0.05 eV) when the μ_3_-O is present. The PDOS shown in Figure S26 indicates that Fe initiates and dominates the unoccupied states, making it the most likely site to accept the photogenerated electrons. Upon illumination, the Fe centers adopt a delocalized Fe^2+^ character, enabling electron donation to the adsorbed N_2_. Following electron transfer, the Fe^2+^ states revert to Fe^3+^, with the central μ_3_-O likely stabilizing the transient Fe^2+^ configuration.

### Stability and Recyclability

Given that long-term performance is as important as initial activity, catalyst stability was evaluated. The structural integrity of the MOFs after catalysis was confirmed by PXRD (Figure S35). Inductively coupled plasma analysis of the reaction supernatant revealed only minor Fe leaching (Table S8). SEM images before and after catalysis showed no change in morphology, indicating good stability of the catalysts (Figure S36). In contrast, the molecular analogue released significantly higher Fe content under identical conditions, consistent with the loss of crystallinity observed in the postcatalysis PXRD pattern of the complex (Figure S35c). In addition, XPS spectra recorded before and after photocatalysis revealed only slight shifts of the Fe 2p binding energies, for all samples (Figures S6 and S7). Importantly, no change in oxidation state was observed, confirming the electronic stability of the framework under reaction conditions.

F-MIL-101­(Fe) demonstrated the highest photocatalytic performance and retained its activity for up to five consecutive cycles (Figure 4c). Regarding their hydrophilicity, all MOF samples showed complete wetting (0° contact angle, Figure S37), due to strong capillary forces of porous materials. Furthermore, dynamic vapor sorption (DVS) measurements revealed a similar water uptake capacity of MIL-101­(Fe), NH_2_-MIL101­(Fe), and F-MIL-101­(Fe) (Figure S38). Due to the strong binding of water to the SBU a large hysteresis is observed. Collectively, the characterization of MIL-101­(Fe) and its monofunctionalized derivatives, alongside photocatalytic testing and mechanistic studies, reveals how ligand chemistry modulates the properties and performance of this specific framework. Similar trends in photocatalytic activity were observed, confirming that the conclusions drawn for MIL-101­(Fe) are not strictly system-specific. The observed behavior therefore supports that the same underlying principle - namely the role of proton availability - remains critical for both frameworks. We envision that this strategy could be extended to other photocatalytic systems, providing a versatile approach for rationally tuning photocatalytic performance in functional materials.

## Conclusions

We synthesized a series of functionalized, photoactive MIL-101­(Fe) MOFs to probe how ligand chemistry governs photocatalytic N_2_-to-NH_3_ conversion. In this system, the μ_3_-oxo-centered trinuclear Fe cluster facilitates redox cycling through delocalized Fe^2+^ photoexcited states, which drive N_2_ activation and stepwise protonation. By systematically varying the ligand, we disentangled electronic effects on Fe electron density, from protonation dynamics governed by active-site accessibility and functional group charge. Ligand substitution was correlated with N_2_ binding and proton availability using complementary spectroscopic techniques (XPS, FTIR) and surface characterization methods (N_2_-TPD, zeta potential), while TRIR spectroscopy revealed that ligand chemistry also controls the lifetimes of photoinduced charge-separated states following partial ligand dissociation at the Fe-oxo cluster.

Among the tested variants, fluorinated MIL-101­(Fe) emerges as the benchmark, combining strong N_2_ activation, favorable proton transfer kinetics, long-lived redox states, and high recyclability. In contrast, NH_2_-functionalization reduces N_2_ chemisorption, while also acting as a hole trap that localizes charge at the nitrogen atom and limits proton availability, thereby being detrimental to photocatalytic activity (Figure 5). Similarly, NO_2_-functionalization, despite enhancing N_2_ chemisorption, withdraws electron density and localizes photogenerated holes, limiting proton availability and overall catalytic turnover.

**Figure 5:**
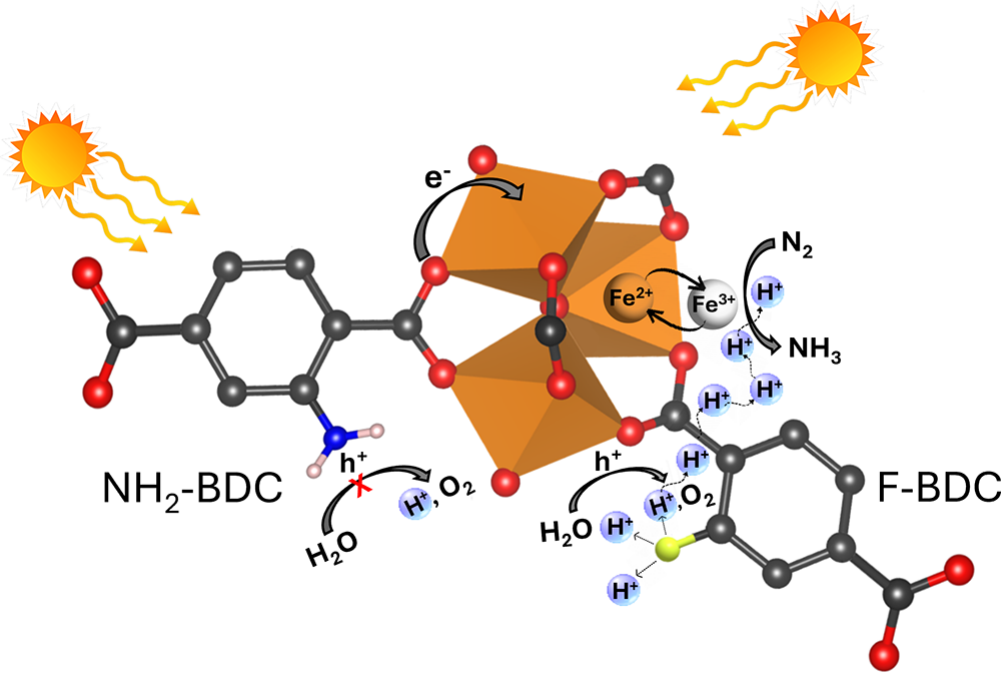
Schematic illustration of rate-determining steps in the MIL-101­(Fe) SBU with NH_2_-BDC and F-BDC ligands, showing hole trapping at the N atom of NH_2_BDC versus favorable proton delivery toward Fe centers with F-BDC.

## Supplementary Material



## Notes

Material synthesis; additional structural and spectroscopic characterization; details of photocatalytic experiments and detection; DFT calculation details; optimized geometries; atomic coordinates; data regarding catalyst stability (PDF)
