# Supplementary material for: Photocatalytic Ammonia Synthesis using Fe-Based MOFs: The Role of Ligand Functionalization
Source: J Am Chem Soc. 2026 May 19;148(21):21382–92. doi: 10.1021/jacs.5c22833 (PMC13244460; doi:10.1021/jacs.5c22833)
Supplement: Supplementary file 1 [file ja5c22833_si_001.pdf]

Supporting Information

Photocatalytic Ammonia Synthesis with Fe-based MOFs: The Role of Ligand Functionalization

Jana Bischoff[1], Cornelia von Baeckmann[1], Shaghayegh Naghdi[1], Adrian Ertl[1], Vasily Vorobyev[2], Anastasiia Naryshkina[3], Lakhanlal[4], Hanspeter Kählig[5], Laura Kronlachner[6], Robert T. Woodward[3], Freddy Kleitz[7], Andreas Limbeck[6], Maytal Caspary Toroker[4,8,9], Amanda J. Morris[2] and Dominik Eder[1*]

[1] Institute of Materials Chemistry, TU Vienna, Getreidemarkt 9, 1060 Vienna, Austria

[2] Inorganic and Energy Chemistry, Department of Chemistry, Virginia Tech, Blacksburg, VA, 24060, USA

[3] Institute of Materials Chemistry and Research, Faculty of Chemistry, University of Vienna, Währinger Straße 42, Vienna, 1090 Austria

[4] Department of Materials Science and Engineering, Technion - Israel Institute of Technology, Haifa 3200003, Israel

[5] Department of Organic Chemistry, University of Vienna, Währinger Straße 38, 1090 Vienna, Austria

[6] Institute of Chemical Technologies and Analytics, TU Vienna, Getreidemarkt 9, 1060 Vienna, Austria

[7] Department of Functional Materials and Catalysis, University of Vienna, Währinger Straße 42, 1090 Vienna, Austria

[8] The Nancy and Stephen Grand Technion Energy Program, Technion - Israel Institute of Technology, Haifa 3200003, Israel

[9] Resnick Sustainability Center for catalysis, Technion – Israel Institute of Technology, Haifa 3200003, Israel

Table of Contents:

S1. Materials and Methods

S2. Materials Characterization

S3. Photocatalytic Activity

S4. Catalyst Stability

S5. DFT Structure (with Energy)

S6. Supplementary Tables

S1. MATERIALS AND METHODS

All starting chemicals were commercial and used without any purification. 1,4 - benzene dicarboxylic acid ($C_8H_6O_4$, H_2BDC, ≥ 99%), 2-Bromoterephthalic acid ($C_8H_5BrO_4$, Br-BDC 95%), 2-Nitroterephthalic acid ($C_8H_5NO_6$, NO_2-BDC ≥99%), 2-Aminoterephthalic acid ($C_8H_9N_1O_4$, NH_2-BDC ≥ 99%), iron (III) chloride hexahydrate ($FeCl_3 \cdot 6H_2O$, ≥99%), 4-Dimethylaminobenzaldehyde (C9H11NO ≥99%) were purchased Sigma Aldrich. 2-Fluoroterephthalic acid ($C_8H_5FO_4$, 98%, F-BDC) was purchased from Angene Chemical. 2-(Trifluoromethyl)terephthalic acid was purchased from BLD Pharmatech GmbH. Hydrazine monohydrate ($H_4N_2 \cdot H_2O$, > 98%) was purchased from TCI Tokyo Chemical Industry (TCI). N,N-Dimethylformamide (DMF, 99.8%), deuterium oxide (D_2O); 99.8 atom % D, Sulfamic acid (HSO_3NH_2); 99% and N-(1-naphthyl)ethylenediamine dihydrochloride ($C_{10}H_7NHCH_2CH_2NH_2 \cdot 2HCl$); 98+ %, asulfonamide ($C_6H_8N_2O_2S$); 99 % and sodium benzoate ($C_7H_5NaO_2$); 99 % were purchased by Thermo Scientific Chemicals. $^{15}N_2$ isotope (98 atom %) was purchased by Sigma Aldrich. Deuterium chloride, 20 wt% in deuterium oxide; 99.5 atom %D and dimethyl-d_6 sulfoxide (DMSO-d6); 99.8 atom %D were purchased from abcr GmbH. Nessler's reagent was purchased from Merck. HPLC-grade water (PanReac AppliChem TWI Reagents, 1 MΩ·cm resistivity) was used in all experiments.

Synthesis of R-bdc-MIL101(Fe). Pristine MIL-101(Fe) was synthesized following the method reported in literature[1]. Briefly, 2.45 mmol of iron chloride hexahydrate ($FeCl_3 \cdot 6H_2O$) and 1.24 mmol of terephthalic acid (H_2BDC) were dissolved in 15 mL of dimethylformamide (DMF) and stirred for 15 minutes at 300 rpm to ensure homogenization. The resulting mixture was transferred to a 25 mL Teflon-lined stainless-steel autoclave and heated at 110 °C for 24 hours, followed by cooling at a rate of 10 °C min^{-1}. After cooling to room temperature, the solid product was recovered by centrifugation, washed twice with DMF, and then washed three times with methanol. Finally, the product was dried in a vacuum oven at 80 °C for 24 hours to remove any residual solvents. Bromo-, nitro-, fluoro-, amino- and trifluoro-functionalized MIL-101(Fe) derivatives (Br-MIL-101(Fe), NO_2-MIL-101(Fe), F-MIL-101(Fe), NH_2-MIL-101(Fe), and CF_3-MIL-101(Fe) respectively) were prepared using a same procedure. In each case, functionalized terephthalic acid (Br-BDC, NO_2-BDC, F-BDC, NH_2-BDC or CF_3-BDC) was mixed with $FeCl_3 \cdot 6H_2O$ in DMF in the same molar ratio (2:1 Fe to ligand) and then proceeded the same way. Mixed-ligand xNH_2-MIL-101(Fe) materials with varying ratios of NH_2-BDC to BDC were synthesized following the same procedure, with the addition of a specific amount of both ligands to achieve NH_2-BDC molar percentages of 2%, 5%, 17%, and 50%.

Synthesis of R-bdc-MIL-88B(Fe). MIL-88B(Fe) and F-MIL-88B(Fe) were synthesized according to a previously reported procedure[2] with minor modifications. $FeCl_3 \cdot 6H_2O$ (3.3 mmol) and H_2BDC or F-BDC (3.3 mmol) were dissolved in DMF (15 mL) and stirred at room temperature for 2 h. The solution was

to room temperature, the solid was isolated by centrifugation, washed with DMF (2 × 15 mL) and ethanol (3 × 15 mL), and dried under vacuum at 60 °C overnight.

Fe_3O-benzoate Complex Synthesis. The molecular MOF analogue, Fe3O-Bz cluster or complex $[Fe_3O(PhCOO)_6(H_2O)_3]ClO_4$ was synthesized based on the previous report3. Aqueous solution of $FeCl_3$•$6H_2O$ solution (13.5 g, 0.05 mol, 50 ml), was slowly added to an aqueous solution of PhCOONa (25g, 0.17 mol, 300 ml). The formation of tan-pink was observed immediately after adding the metal salt. The resulting powder was vacuum filtered and washed several times with deionized water and finally dried at 50 °C under vacuum overnight. A 2.0 g of solid was dispersed in 40 mL of anhydrous ethanol. 6 g of 20% $HClO_4$ was added dropwise to the suspension and stirred until dissolved. For approximately 10 minutes the solution was heated on the steam bath and gravity filtered. 30 ml of boiling water was added to the filtrate and the product started precipitating in a crystalline needle-like form. To finalize the crystallization, it was cooled in the fridge at 4 °C for 4 hours. Afterwards, the resulting complex was washed with small aliquots of water acetone mixture (1:1 v/v, 15 ml in total) and finally dried at 50 °C under vacuum overnight.

Electrodes Preparation. The catalyst ink was prepared by dispersing the photocatalyst (7 mg) in a mixture of isopropanol (0.5 mL) and 5 wt % Nafion® solution (16 μL). The suspension was sonicated in an ultrasonic bath for 60 min to ensure homogeneous dispersion. An aliquot (20 μL) of the resulting ink was drop-cast onto a fluorine-doped tin oxide (FTO) glass substrate and dried in air at room temperature. The casting was repeated 5 times to obtain desired thickness.

Powder X-ray Diffraction (PXRD). The samples were grounded and placed as powders on silicon single crystal sample holder. All PXRD measurements were carried out using PANalytical X'Pert Pro multi-purpose diffractometer (MPD) in Bragg Brentano geometry operating with a Cu anode at 45 kV, 40 mA, equipped with a BBHD Mirror and an X-Celerator multichannel detector. The detector records the signal intensity as a function of an incidence angle giving a diffractogram as a graph of intensity versus 2θ, where the samples were scanned between 2θ angle of 2° and 30°. Sample holders were rotated with 4 s per turn during the measurement. All measurements were conducted with Cu sealed tube K_α and K_β radiation (2:1 ratio) with a wavelength of λ=1.54060 Å at a scan rate of 0.5° min^{-1}.

Attenuated Total Reflectance-Fourier Transform Infrared Spectroscopy (ATR-FTIR). ATR-FTIR spectra were measured on a PerkinElmer FTIR Spectral UATR -TWO with a spectrum two Universal ATR (Single Reflection Diamond) instrument between the range of 4000–400 cm^{-1} with blank spectrum of air taken as a background.

Ultraviolet-visible (UV−Vis) Solution Absorbance/diffuse Reflectance Measurements (DRS). UV-Vis and DRS measurements were performed on a Jasco V-670 spectrophotometer. For DRS, the light was collected using an integrating sphere, and the incident light covered the range of 200–800 nm, with $MgSO_4$

as the background. High-purity water was used as the background for UV–vis measurements. For ammonium, hydrazine, nitrate, and nitrite detection, UV–vis measurements were performed using water as the background.

X-ray Photoelectron Spectroscopy (XPS). XPS measurements were performed using a custom-built instrument from SPECS with samples being measured as powders on double-sided carbon tape. The analysis utilized a monochromatized Al-Kα X-ray source (μ-Focus 350) with an excitation energy of 1486.71 eV. Emitted photoelectrons were detected with a hemispherical Phoibos WAL-150 analyzer where pass energies of 100 eV and 30 eV where used for survey and detailed spectra, respectively.

Gas Adsorption. N_2 physisorption measurements at 77 K were conducted using 60-70 mg of samples loaded in 12 mm Micrometrics BET adsorption cell Flex instrument (Micromeritics, Norcross, GA, USA). Prior to the measurement, the sample was outgassed under vacuum at 120 °C for 24 h. The specific surface area was obtained using the BET equation in the range of 0.008 – 0.08 0.008 – 0.08 p/p^0, and the corresponding pore size distributions were determined from the adsorption branch of the isotherm using the kernel of non-local density functional theory (NLDFT) with a 3Flex surface analyzer. The micropore volume was determined by t-plot analysis, considering the N_2 adsorption data in the thickness range of 0.4 - 1.9 nm.

Nuclear Magnetic Resonance (NMR). All qualitative ^1H NMR spectra were recorded using a 250.13 MHz spectrometer equipped with a 5 mm inverse-broad probe head and a z-gradient unit at 25 °C. Chemical shifts (δ) are reported in ppm and coupling constants (J) in Hz. The resonance of residual DMSO for DMSO-d_6 (2.5 ppm) is used as internal reference. The samples were digested with a 1 M NaOH solution and stirred overnight at 60 °C. The resulting $Fe(OH)_3$ precipitate was separated using a 0.22 μm PTFE syringe filter. The dissolved ligand was recrystallized by slow acidification with DCl. The recrystallized ligand was then dissolved in d_6-DMSO for NMR analysis.

Nuclear Magnetic Resonance (NMR) for NH_4^+ Detection. To detect the weak signals for NH_4^+, samples in 90% H_2O / 10% D_2O were measured on a Bruker Avance III HD 700 NMR spectrometer (Bruker BioSpin, Ettlingen, Germany) using a 5 mm helium cooled cryo probe (QCI-F) with z axis gradients and automatic tuning and matching accessory. The resonance frequency for 1H NMR was 700.40 MHz. Water suppression was achieved using WATERGATE with a double pulsed field gradient spin-echo together with a binominal hard-pulse sequence. In total 1024 transients were acquired resulting in an experiment time of 1.5 hours. Referencing was applied external using DSS.

Nitrogen Temperature-Programmed Desorption (N_2-TPD). Temperature-programmed desorption of N_2 was performed using 70 mg of each catalyst at atmospheric pressure, with a BelCat II adsorption apparatus from Microtrac. The catalyst was loaded in a continuous-flow fixed-bed quartz reactor. Before each run, the adsorbed gas and solvent molecules were removed by pretreatment at 120 °C for 3 hours in a He

atmosphere (50 mL min^{-1}). The samples were then cooled down to 50 °C in a He flow of the same flow rate, and at this temperature, N_2 was purged for 2 hours (50 mL min^{-1}), followed by He purging for 30 minutes to remove excess N_2. The samples were then heated in He atmosphere up to 230 °C (1 °C min^{-1}) and held at that temperature for 1 hour. respectively, were analyzed by an online quadrupole mass spectrometer (QMS, Prisma Plus QMG220, Pfeiffer Vacuum) equipped with a secondary electron multiplier (SEM) detector.

Inductively Coupled Plasma Mass Spectrometry (ICP-MS). The iron concentration in the samples was determined using an iCAP TQ ICP-MS (ThermoFisher Scientific, Bremen, Germany). For the sample introduction, an ESI 4DX autosampler equipped with an ESI FAST system (1 mL sample loop) (Elemental Scientific Inc., Omaha, NE, USA), a concentric PFA nebulizer, and a quartz cyclonic spray chamber was used.For the ICP-MS analysis, a plasma power of 1500 W, a nebulizer gas flow of 1.0 L/min argon (Ar 5.0), a cool gas flow of 14 L/min argon (Ar 5.0), and an auxiliary gas flow of 0.8 L/min argon (Ar 5.0) were applied, and the dwell time was set to 100 ms. Data acquisition was performed using Qtegra software provided by the instrument manufacturer. Signal quantification was based on external calibration with aqueous Fe standard solutions in a general concentration range of 1 to 100 µg/L. For the determination of Fe content, the isotopes ^{54}Fe and ^{56}Fe were monitored. ^{59}Co was used as an internal standard. The analysis was performed using kinetic energy discrimination (KED) mode, which utilizes a collision cell containing He 5.0 as the collision gas with a gas flow rate of 3.5 mL/min and a kinetic energy barrier of 3 V. All samples were diluted 1/50 and spiked with 1 ppb Cobalt as an internal standard to correct for possible instrumental drift and matrix effects. The Fe leaching (%) after photocatalysis was calculated from ICP-MS data obtained as Fe concentration in µg/L (ppb) in the supernatant solution. Divide the total Fe mass in the solution (µg) by the theoretical Fe mass in the photocatalyst sample used (µg), then multiply by 100:

$$\text{Fe leaching (\%)} = \frac{\text{Fe mass in solution (µg)}}{\text{Fe mass in sample (µg)}} \times 100$$

Thermogravimetric Analysis (TGA). TGA measurements were conducted on a PerkinElmer Thermogravimetric analyzer 8000 (Waltham, USA), using an aluminum oxide (Al_2O_3) crucible. Synthetic air was used as treatment gas with a ramp rate of 10 °C min^{-1}.

Scanning Electron Microscopy (SEM). SEM was carried out using a Zeiss Supra 55 VP microscope. All samples were sputtered with Au/Pd prior analysis. Images were analyzed using the associated software. The scale bars of the obtained micrographs were postprocessed for better visualization.

Water Contact Angle Measurements. The MOF samples were pressed into pellets on the SEM grids using a manual press, and as that activated at 120 °C under vacuum. Static water contact angles were measured using a drop shape analyzer (DSA30, Krüss GmbH, Hamburg, Germany) with an ADVANCE software-controlled dosing using a sessile drop method. A droplet of ultrapure water (2 µL) with dosing rate of 3 µls−1 and needle movement speed of 200 mm min-1 was deposited onto the sample surface, and contact

angles were recorded after time delays of 1, 10, 30, and 60 seconds. For each sample, measurements were performed on three different spots, and the average values are presented.

Dynamic Vapor Sorption (DVS). DVS isotherms were collected using Surface Measurement Systems DVS-Resolution. Samples were activated at 120 °C in a vacuum oven overnight before approximately 10 mg of sample was weighed into a quartz crystal pan for measurement. Measurements began at 0% RH to remove residual water before being increased to the desired RH using deionized water. Experiments were carried out at 25 °C. Isotherms were recorded up to 90% RH, using a step increment of 10% RH. Each step was equilibrated for 5 h, prior to measurement.

Zeta-potential Measurements. Zeta-potential measurements were performed using a Malvern Zetasizer Nano ZS. Stable aqueous suspensions of the material in water (pH 3.2; 1 - 5 mg mL^{-1}) were obtained by ultrasonic bath treatment. Prior to zeta-potential measurements, a standard solution with a zeta-potential of −42 ± 6 mV was measured to ensure correct calibration. Zeta-Potentials are reported as an average of six measurements, with each measurement consisting of 20-200 runs.

Photocatalytic Experiments. Photocatalytic nitrogen conversion experiments were conducted under continuous visible light irradiation (AM 1.5G, $\lambda \geq 420$ nm, 23 mW cm^{-2}) using a 150 W Xenon arc lamp. The reactions were carried out in a custom-made quartz reactor equipped with a circulating water jacket to maintain a temperature of 15 °C. Catalysts (5 mg) were pre-activated at 120 °C under vacuum for 12 hours to remove adsorbed gases. The catalyst was then suspended in 10 mL of ultrapure water, purged with Ar for 15 minutes, and saturated with N_2 at a flow rate of 80 mL min^{-1} for 30 minutes in the dark. After sealing the system, continuous light irradiation was applied for 1 hour. Following irradiation, the suspension was collected, and the catalyst was removed via syringe filtration. The NH_4^+ concentration in the liquid phase was quantified using Nessler's reagent colorimetric method. Standard deviations were calculated from triplicate measurements.

Spectrophotometric Ammonium (NH_4^+) Detection. Ammonia concentration was determined using Nessler's reagent method. In this method, Nessler's reagent, which consists of mercury (II) iodide in an alkaline solution, reacts with ammonia to form a colored complex. The reaction suspension was first filtered through a 0.22 μm PTFE filter to remove any particulates that could interfere with the analysis. Subsequently, 1 mL of Nessler's reagent was added to the sample. This reagent reacts with ammonia, resulting in a color change from yellow to orange to reddish-brown, depending on the ammonia concentration. The mixture was allowed to stand for 20 minutes to ensure complete color development. After this period, the absorbance of the solution was measured at 420 nm using a UV-Vis spectrometer. The obtained absorbance values were then converted into ammonia concentrations using a pre-established calibration curve, derived from triplicate standard preparations. The production rate was calculated using the following equation:

$$r_{NH_4^+} = \frac{n_{NH_4^+}}{m_{cat} \cdot t} \; [r_{NH_4^+}] = \frac{\mu mol}{g \cdot h}$$

Spectrophotometric Hydrazine (N_2H_4) Detection. Hydrazine (N_2H_4) was quantitatively determined using the Watt and Chrisp method. In this approach, 4-(dimethylamino) benzaldehyde was used as a color reagent, prepared by dissolving 5.99 g of the compound in a mixture of 30 mL of concentrated hydrochloric acid and 300 mL of ethanol. To detect hydrazine, 5 mL of the reaction solution was filtered and then mixed with 5 mL of the color reagent. The reaction mixture was allowed to develop color in the dark for 20 minutes, ensuring complete color formation. The resulting color intensity was measured using UV-Vis spectrophotometry at 455 nm. The hydrazine concentration was determined by comparing the absorbance with a calibration curve generated from standard $N_2H_4 \cdot H_2O$ solutions.

Spectrophotometric Nitrate (NO_3^-) Detection. For determining the NO_3^- concentration, 4 mL of standard or sample was mixed with 0.1 mL of 1M HCl and 0.1 mL of sulfamic acid (99.5%) aqueous solution (0.8 wt.%.). The solutions were shaken and left for standing for 10 minutes. After it, the absorbance was measured using UV-vis spectrophotometer. The NO_3^- concentration was estimated from absorbance value A ($A = A_{220nm} – 2 \times A_{275nm}$) and the corresponding calibration curve was derived.

Spectrophotometric Nitrite (NO_2^-) Detection. The colorimetric reagent was prepared by mixing 0.02 g of N-(1-naphthyl) ethylenediamine dihydrochloride (≥98%), 0.4 g of sulfonamide (≥99%) and 1 mL of phosphoric acid (85 wt. % in H_2O) with 5 mL of deionized water. Subsequently, the colorimetric reagent (0.1 mL) was added to sample solutions and stood for 20 min. The absorbance at ca. 540 nm was recorded using UV–vis spectrophotometry, and thus, the concentration of generated NO_2^- is determined. The corresponding standard curves were derived from measurements with known concentrations of NO_2^- solutions.

Hydrogen Evolution Reaction (HER) Test. Typical closed reactor HER test to exclude concurrent proton-consuming reactions. The test was conducted by reproducing N_2RR photocatalytic reaction parameters with 5 mg of catalyst powder suspended in 10 mL of HPLC water without any sacrificial agent. The suspension was purged with Ar at 50 mL min^{-1} for 15 minutes, then saturated with nitrogen gas for 30 minutes. The system was finally illuminated with a 150 W Xe lamp with a 420 nm cut-off filter (23 mW cm^{-2}) for 1 hour. A gas sample of 200 µL was taken from the headspace and analyzed using GC to quantify the total amount of H_2 produced. Quantification was done using a gas chromatograph calibrated with a six-point calibration ranging from 100 to 50 000 ppm.

$^{15}N_2$ Isotope Labeling Experiment. To verify the origin of generated ammonia, $^{15}N_2$ isotope labelling experiments were performed. The parameters were identical to the ones in standard photocatalytic experiments. After 15 minutes purging with the argon, the mixture was continuously stirred in the dark purging with the $^{15}N_2$ at the flow rate of 20 ml min^{-1} for 30 minutes to achieve the $^{15}N_2$ saturated solution

and subsequently irradiated for 1h. After the photoreaction, the 500 µL reaction solution was transferred by the $^{15}N^2$ purged syringe over a Teflon cannula into a screw-cap NMR tube with septum containing 50 µL 0.5 M H_2SO_4, followed by the measurement of ^{1}H-NMR spectroscopy.

Nanosecond Time-Resolved Infrared (TRIR) Measurements. A MOF suspension was prepared by dispersing the synthesized particles in acetonitrile (3% w/w). Prior to spin coating, the CaF_2 substrate (diameter = 25.4 mm, 1 mm thick, Crystran Ltd.) was cleaned with O_2 plasma for 15 min. The MOF film was prepared by spin-coating the suspensions (50 µL) of MOF onto the substrate at 1000 or 2000 rpm for 30 s. The spin-coating process was repeated for 1 more time to obtain thicker films. The films were dried at 80 °C for 30 minutes after the depositions in N_2 atmosphere.Time-resolved infrared (TRIR) absorption measurements for spin-coated samples on transparent substrate were conducted with a Magnitude Instruments inspIRe system. The probe beam was an IR Globar, while the pump source was an external diode-pumped solid-state Nd:YAG laser outputting the 2nd harmonic wavelength (532 nm, ~2 ns pulse length). Time-resolved spectral maps from 1000 to 1800 cm^{-1} were measured from samples exited at 7 kHz repetition rate (532 nm, 1200 mW) in the 131 µs time window. Readouts of a total of $\sim 5\cdot 10^5$ laser shots were averaged. The kinetic measurements at a signle wavenumber were performed on a samples excited at 800 Hz repetition rate (532 nm, 136 mW) in the 524 µs time window ($1.6\cdot 10^5$ laser shots averaged). The pump beam had an area of 0.19 cm^2 and the pulse energy was calculated to be 170 µJ based on measurements with Thorlab power meter PM100D equipped with thermal head S401C. Measurements were conducted in air

Cycling Experiments. For photocatalytic cycling tests, 30 mg of F-MIL-101(Fe) was dispersed in HPLC-grade water and irradiated under visible light ($\lambda \geq 420$ nm) for 1 hour in a quartz beaker. After each irradiation step, the catalyst was recovered by centrifugation and dried. A 5 mg portion of the dried material was weighed and used in a standard 1-hour photocatalysis test in the photoreactor to assess NH_3 production. This process was repeated over five cycles. After the final cycle, the remaining catalyst was collected and analyzed by PXRD to evaluate structural stability.

Density Functional Theory (DFT). A spin polarized DFT+U study was carried out on this system with U-J value of 6 eV[4] for Fe using Vienna ab-initio simulation package (VASP) 5.4.4[5,6,7]. Considering the size of the unit cell of this MOF, a cluster is formed and kept in a vacuum of 28 Å × 28 Å × 28 Å to minimize periodic interactions. Such a cluster model has been used previously in the literature[3,8] For this calculation, the energy cutoff of 600 eV is chosen after performing energy convergence tests with several energy cutoffs. Considering the large size of the system, a gamma-centered single k-point (1×1×1) is used. The electronic convergence criteria was set to 10^{-5} eV whereas structural relaxation was allowed until the force acting on each atom became less than 0.03 eV/Å. Nitrogen adsorption is studied on this MOF and nitrogen adsorption energy is calculated using the equation given below.

$$G_{ad}(N_2) = E_{N_2\ on\ MOF} - E_{MOF} - E_{N_2}$$

Also, the density of states, Bader charge, charge density difference, and electron localization function (ELF) were calculated.

Electrochemical measurements. Electrochemical measurements were performed in a custom-made single-compartment cell using a standard three-electrode configuration. A 150 W xenon lamp equipped with a $\lambda \geq 420$ nm cutoff filter was used as the light source under conditions identical to those employed for photocatalytic experiments (23 mW cm^{-2} power density). The catalyst-coated FTO substrate served as the working electrode, a Pt foil as the counter electrode, and an Ag/AgCl (3 M KCl) electrode as the reference. A 0.1 M Na_2SO_4 aqueous solution was used as an electrolyte. Prior to each measurement, the headspace of the sealed cell was purged with N_2 for 45 min. Linear sweep voltammetry (LSV) was performed at a scan rate of 0.05 mV s^{-1} over a potential range of 0.05–1.8 V to determine the onset potential. Chronoamperometry was conducted at 1.6 V vs Ag/AgCl for 6 min under continuous illumination. Subsequently, photocurrent measurements were carried out at a constant potential of 1.6 V vs Ag/AgCl for 300 s using pulsed illumination (30 s light on/off cycles), generated by periodically blocking the light source. The photocurrent was obtained by subtracting the dark current as a baseline.

Photocatalysis at Different pH Values. Photocatalytic experiments at different pH values were conducted under standard conditions. After dispersing the catalyst in ultrapure water, the pH was adjusted to 7, 8, and 10 using a 0.01 M NaOH solution added dropwise, while monitoring with a pH electrode. Following the reaction, the pH was readjusted to neutral prior to Nessler's analysis to shift the NH_3/NH_4^+ equilibrium toward NH_4^+, ensuring that ammonia is retained in the aqueous phase for reliable detection.

S2. MATERIALS CHARACTERIZATION

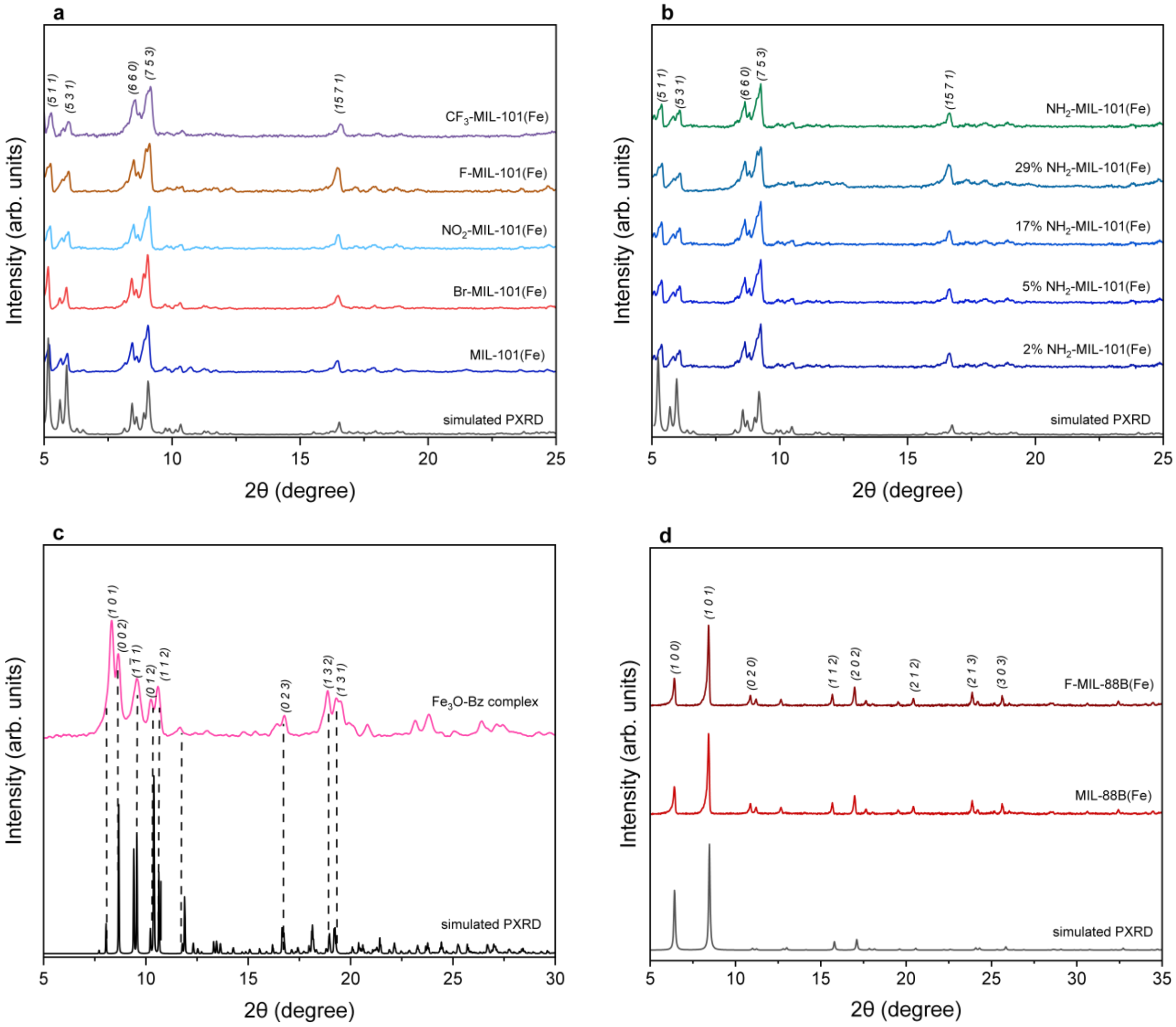

Figure S1. Powder X-ray diffraction (PXRD) patterns of (a) as-prepared single-ligand MOF samples (MIL-101(Fe), F-MIL-101(Fe), NO_2-MIL-101(Fe), Br-MIL-101(Fe)), and CF_3-MIL-101(Fe) (b) mixed-ligand xNH_2-MIL-101(Fe) samples and the single-ligand NH_2-MIL-101(Fe), (c) the Fe_3O-Bz molecular complex $[Fe_3O(PhCOO)_6(H_2O)_3]ClO_4$, and (d) MIL-88B(Fe) and F-MIL-88B(Fe). All patterns are presented in comparison to the simulated PXRD patterns of the corresponding MOFs and the complex $[Fe_3O(PhCOO)_6(MeOH)_3](NO_3)(MeOH)_2]$. The main Bragg diffraction peaks, obtained using Rietveld refinement with RIETAN FP, are highlighted.

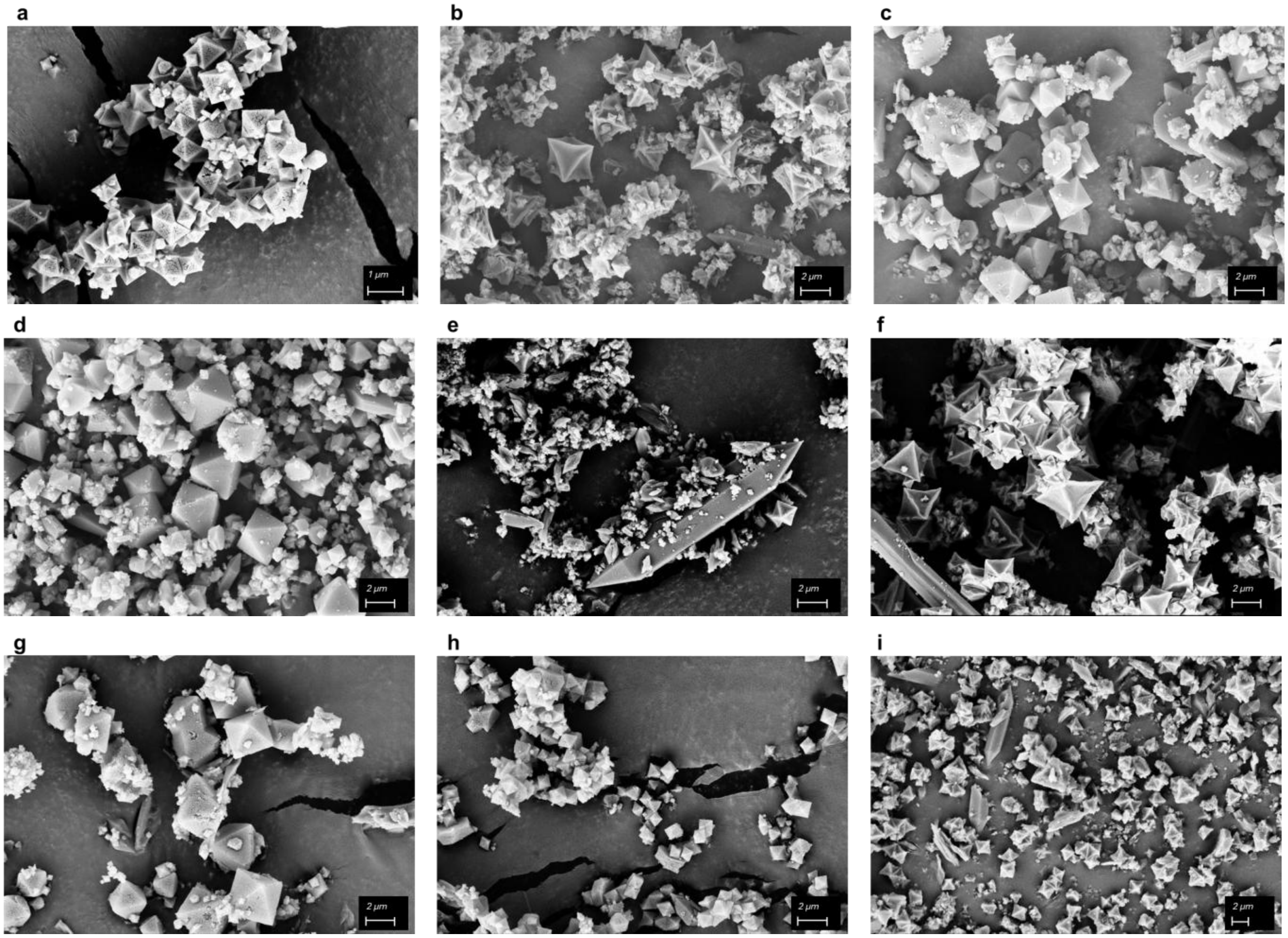

Figure S2. Low-magnification SEM images of all as-prepared MOF samples: (a) MIL-101(Fe), (b)F-MIL-101(Fe), (c) NO_2-MIL-101(Fe), (d) Br-MIL-101(Fe), (e) NH_2-MIL-101(Fe), (f) 2%NH_2-MIL-101(Fe), (g) 5%NH_2-MIL-101(Fe), (h) 17%NH_2-MIL-101(Fe), and (i) 29%NH_2-MIL-101(Fe). The images show particle size and morphological features. The single-ligand MOFs exhibit a characteristic octahedral morphology, which is largely retained in the mixed-ligand MOFs with lower NH_2-BDC content. The 29% NH_2-MIL-101(Fe) sample displays heterogeneous particle shapes, still dominated by octahedral crystals, along with fewer hexagonal micro-spindle particles. In contrast, the NH_2-MIL-101(Fe) sample with higher NH_2-BDC content shows irregular polyhedral crystals and predominantly very small particles, with only a few spindle-shaped crystals visible consistent with literature reports[9],[10]. Additional SEM images of (j) CF_3-MIL-101(Fe), (k) MIL-88B(Fe), and (l) F-MIL-88B(Fe) are included for comparison. The MIL-88B-based samples exhibit the previously reported rod-shaped morphology[11],[12].

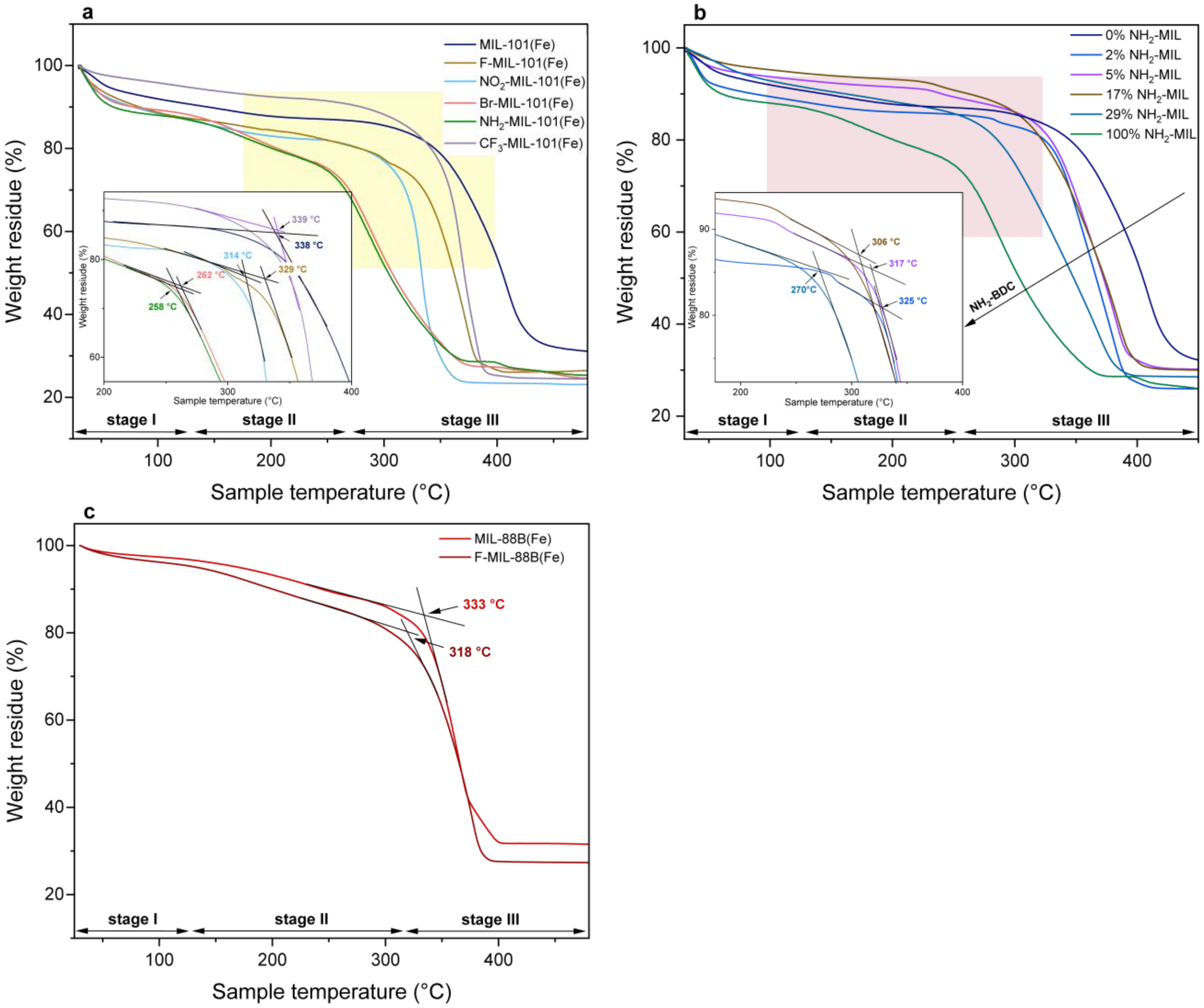

Figure S3. Thermogravimetric analysis (TGA) of MOFs in synthetic air at a constant heating rate of 5 °C min^{-1}: (a) For single-ligand MOFs, the initial weight loss below 120 °C corresponds to adsorbed water, while the loss between 120 and 200 °C arises from residual solvent and water trapped in the pores (MIL-101(Fe), NO_2-, F-, and CF_3-MIL-101(Fe)). In Br- and NH_2-MIL-101(Fe), this stage ends earlier, followed by the departure of terminal –OH/Cl groups, beginning at ~150 °C, compared with >200 °C for F-, CF_3-, NO_2-, and unfunctionalized MIL-101(Fe). All samples undergo thermal decomposition (inset), with Br- and NH_2-MIL-101(Fe) showing the lowest stability (onset at 258 and 262 °C), followed by NO_2-MIL-101(Fe) at 314 °C, F-MIL-101(Fe) at 329 °C, and MIL-101(Fe) and CF_3-MIL-101(Fe) as the most stable analogues (338 and 339 °C, respectively)[13]. (b) For mixed-ligand MOFs, the degradation onset temperature decreases with increasing NH_2-BDC content, with NH_2-MIL-101(Fe) showing a T_{onset} of 325 °C. The 2%, 5%, and 17% NH_2-MIL-101(Fe) samples exhibit distinct slope changes at 283 °C, 233 °C, and 227 °C, respectively, likely corresponding to the removal of the thermally less stable NH_2-BDC ligand. The 50% NH_2-MIL-101(Fe) sample lacks a sharp transition due to its higher content of thermolabile ligands, leading to a more gradual mass loss. Overall, the structural degradation onset temperature of mixed-ligand MOFs decreases progressively with increasing NH_2-BDC

content, reflecting the removal of the less thermally stable ligand. (c) TGA curves of MIL-88B(Fe) and F-MIL-88B(Fe) with decomposition onset temperatures of 333 and 318 °C, respectively.

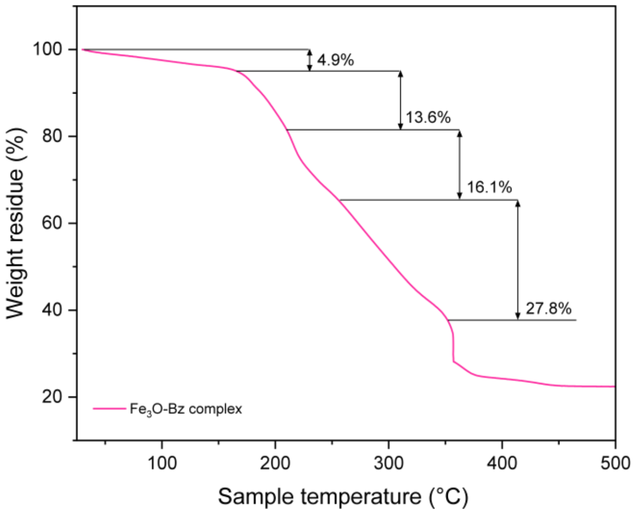

Figure S4. TGA analysis of the Fe_3O-Bz complex. Initial mass loss of ≈ 4.9% below ~150 °C, consistent with removal of three coordinated/lattice water molecules (theoretical 5.1%). Progressive mass losses between ~165 and 352 °C are assigned to stepwise oxidative decomposition of benzoate ligands and volatilization of organic fragments. A pronounced, abrupt mass loss occurs at ≈ 353 °C; this feature is attributed to decomposition of the perchlorate counter-ion and rapid perchlorate-driven oxidation of residual organic fragments, which completes ligand combustion. The residual mass (~22%) at high temperature corresponds to the expected iron oxide residue (theoretical ≈ 22.5%). The presence of ClO_4^- likely sharpens and shifts decomposition features relative to chloride or nitrate analogues and should be considered when comparing thermal behavior across different Fe_3O trinuclear salts. Similar decomposition patterns have been observed in Fe_3O–benzoate complexes described previously[14].

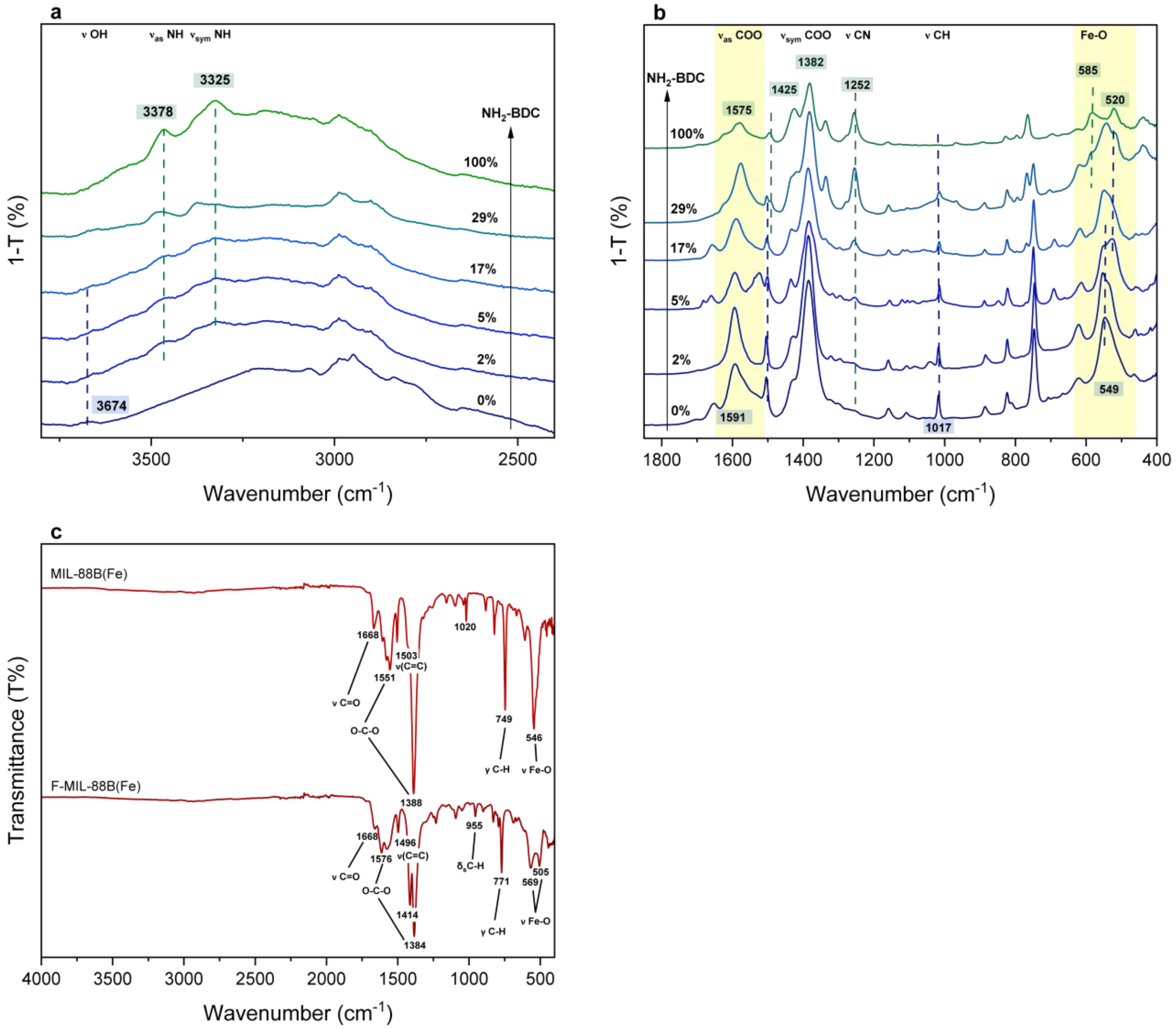

Figure S5. FTIR spectra of as-prepared: (a) Enlarged view of the high-frequency region of mixed-ligand xNH_2-MIL MOFs, highlighting the NH_2 asymmetric and symmetric stretches. (b) FTIR spectra of mixed-ligand xNH_2-MIL-101(Fe) MOFs, with shaded regions indicating key vibrational features. Bands in mixed-ligand MOFs are typically superpositions of those observed in single-ligand MOFs. (c) FTIR spectra of MIL-88B(Fe) and F-MIL-88B(Fe), showing main vibrational features. F-MIL-88B(Fe) exhibits asymmetric coordination, as indicated by Fe–O stretches at 569 and 505 cm^{-1}, and symmetric COO^- stretches at 1414 and 1284 cm^{-1}, similar to those observed in F-MIL-101(Fe).

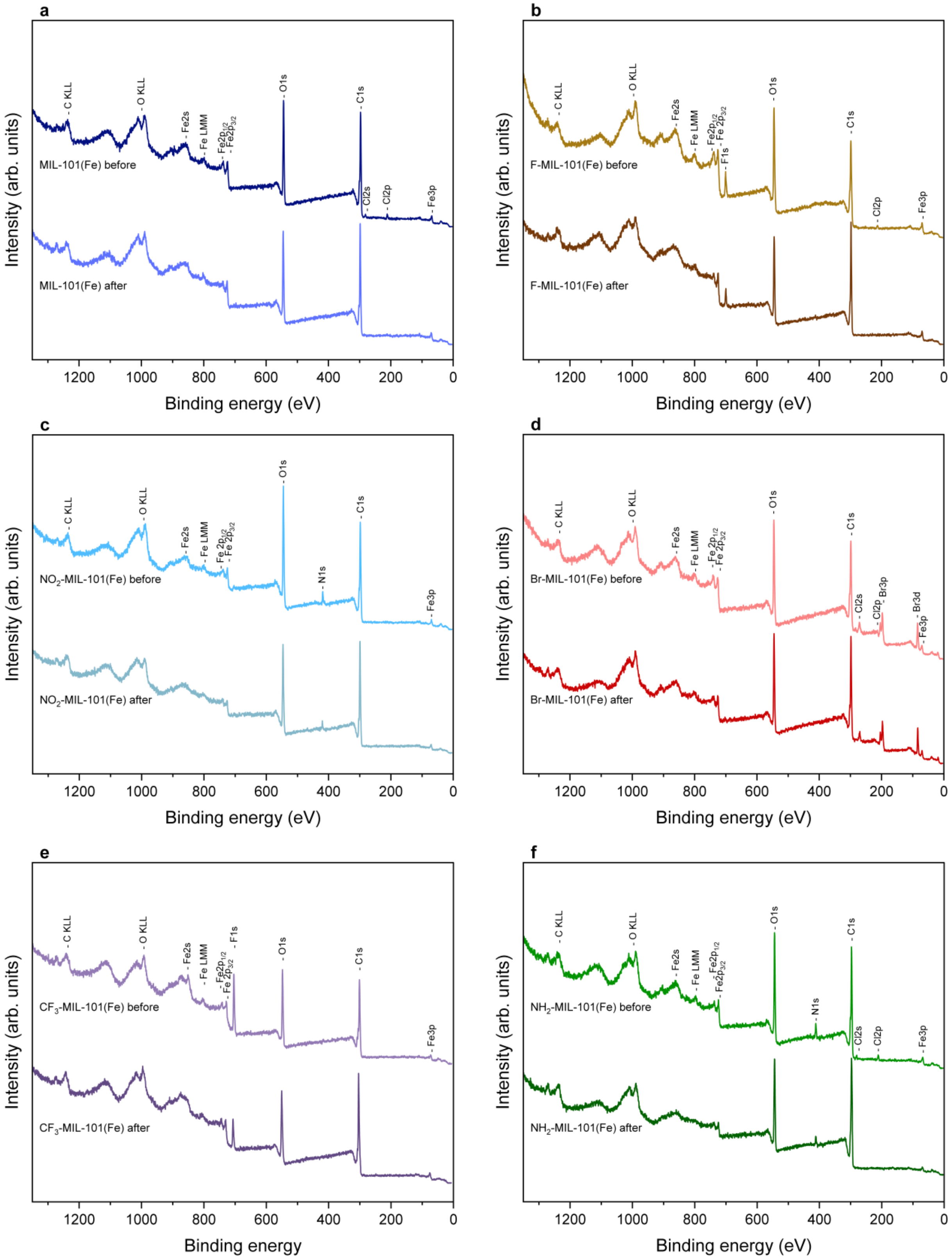
a
b
c
d
e
f
Intensity (arb. units)
Binding energy (eV)
Binding energy
MIL-101(Fe) before
MIL-101(Fe) after
F-MIL-101(Fe) before
F-MIL-101(Fe) after
NO$_2$-MIL-101(Fe) before
NO$_2$-MIL-101(Fe) after
Br-MIL-101(Fe) before
Br-MIL-101(Fe) after
CF$_3$-MIL-101(Fe) before
CF$_3$-MIL-101(Fe) after
NH$_2$-MIL-101(Fe) before
NH$_2$-MIL-101(Fe) after
C KLL
O KLL
Fe2s
Fe LMM
Fe2p$_{1/2}$
Fe2p$_{3/2}$
O1s
C1s
Cl2s
Cl2p
Fe3p
F1s
N1s
Br3p
Br3d
1200
1000
800
600
400
200
0

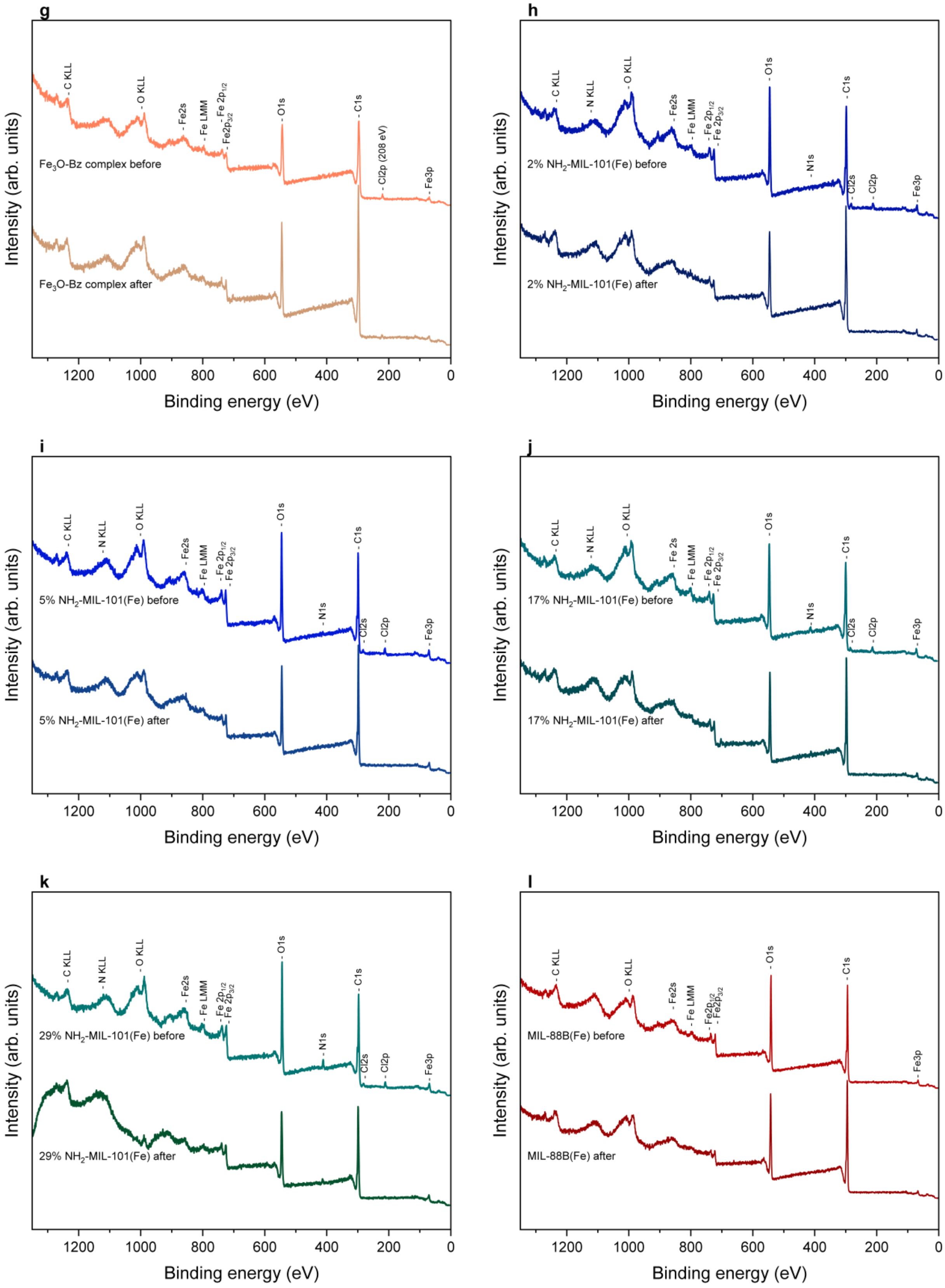
g
h
i
j
k
l
Intensity (arb. units)
Binding energy (eV)
1200
1000
800
600
400
200
0
C KLL
N KLL
O KLL
Fe2s
Fe 2s
Fe LMM
Fe 2p1/2
Fe 2p3/2
Fe2p3/2
Fe2p1/2
O1s
N1s
C1s
Cl2s
Cl2p
Cl2p (208 eV)
Fe3p
Fe3O-Bz complex before
Fe3O-Bz complex after
2% NH2-MIL-101(Fe) before
2% NH2-MIL-101(Fe) after
5% NH2-MIL-101(Fe) before
5% NH2-MIL-101(Fe) after
17% NH2-MIL-101(Fe) before
17% NH2-MIL-101(Fe) after
29% NH2-MIL-101(Fe) before
29% NH2-MIL-101(Fe) after
MIL-88B(Fe) before
MIL-88B(Fe) after

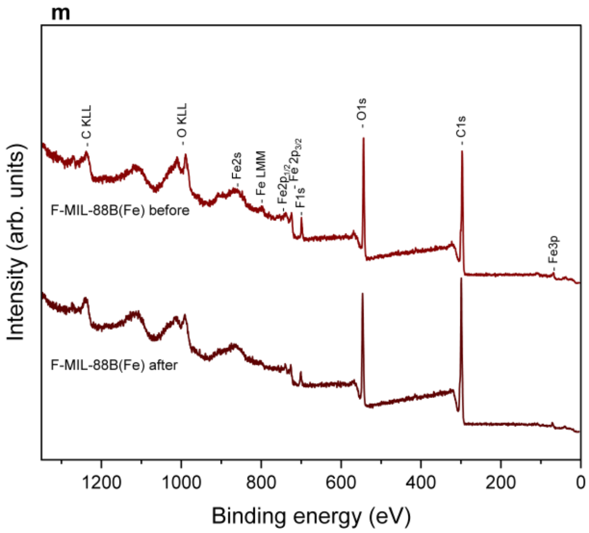

Figure S6. Full XPS spectra of MIL-101(Fe), MIL-88B(Fe), their functionalized derivatives, and a Fe_3O-benzoate complex before and after photocatalysis. (a) Pristine MIL-101(Fe), (b) F-MIL-101(Fe), (c) NO_2-MIL-101(Fe), (d) Br-MIL-101(Fe), (e) CF_3-MIL-101(Fe), (f) NH_2-MIL-101(Fe), (g) Fe_3O-benzoate complex, (h–k) mixed-ligand xNH_2-MIL-101(Fe) series, and (l) MIL-88B(Fe) and F-MIL-88B(Fe). In all cases except Br-MIL-101(Fe), the Cl 2p signal disappears after photocatalysis, consistent with ligand exchange from Cl^- to OH^-. The Cl 2p signal is not observed in CF_3-MIL-101(Fe). Functionalized MOFs exhibit additional peaks corresponding to their substituents. The Fe_3O-benzoate complex shows a higher Fe binding energy, attributed to the presence of a perchlorate counterion.

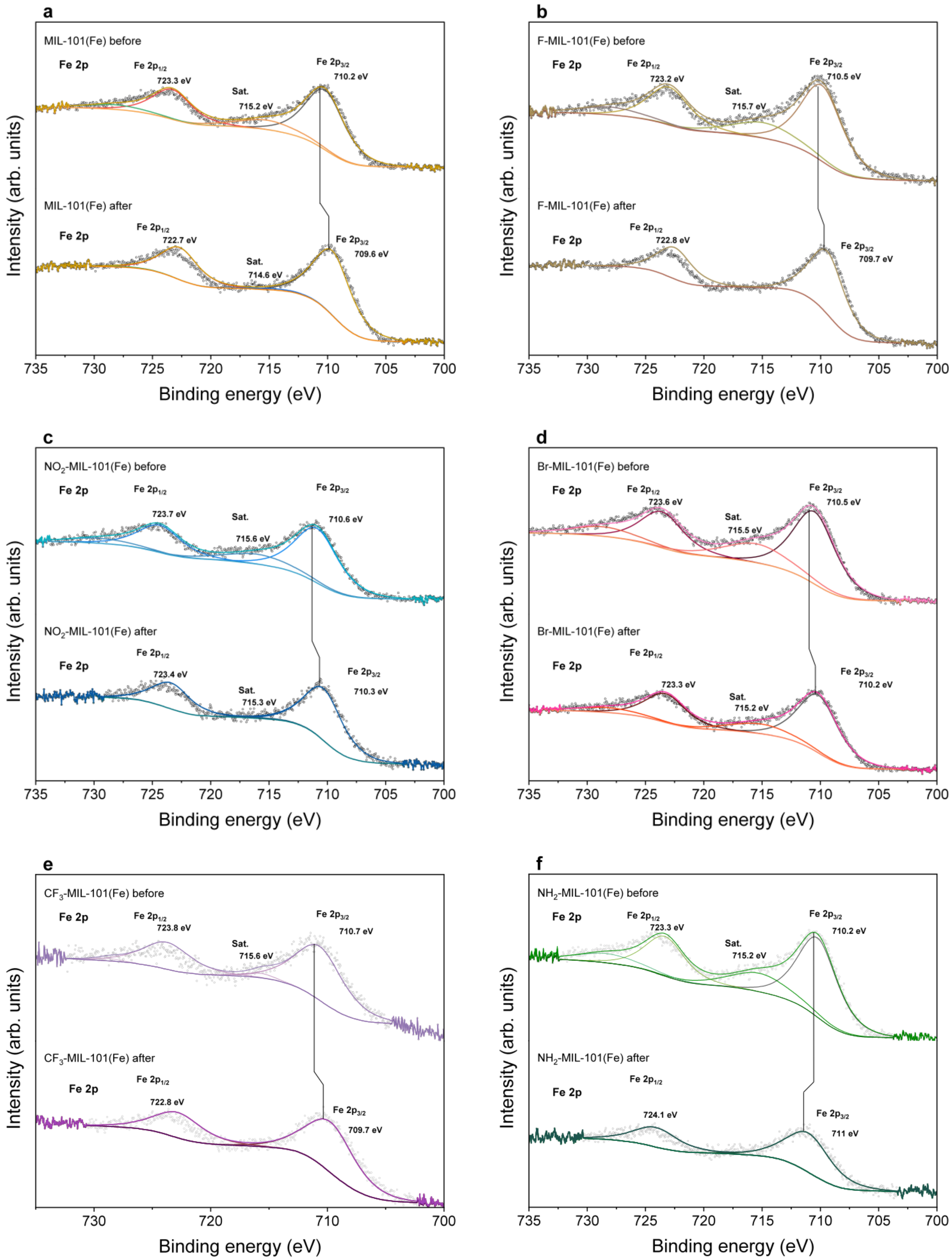
a
MIL-101(Fe) before
Fe 2p
Fe 2p1/2
723.3 eV
Sat.
715.2 eV
Fe 2p3/2
710.2 eV
MIL-101(Fe) after
Fe 2p
Fe 2p1/2
722.7 eV
Sat.
714.6 eV
Fe 2p3/2
709.6 eV
Intensity (arb. units)
Binding energy (eV)
b
F-MIL-101(Fe) before
Fe 2p
Fe 2p1/2
723.2 eV
Sat.
715.7 eV
Fe 2p3/2
710.5 eV
F-MIL-101(Fe) after
Fe 2p
Fe 2p1/2
722.8 eV
Fe 2p3/2
709.7 eV
c
NO2-MIL-101(Fe) before
Fe 2p
Fe 2p1/2
723.7 eV
Sat.
715.6 eV
Fe 2p3/2
710.6 eV
NO2-MIL-101(Fe) after
Fe 2p
Fe 2p1/2
723.4 eV
Sat.
715.3 eV
Fe 2p3/2
710.3 eV
d
Br-MIL-101(Fe) before
Fe 2p
Fe 2p1/2
723.6 eV
Sat.
715.5 eV
Fe 2p3/2
710.5 eV
Br-MIL-101(Fe) after
Fe 2p
Fe 2p1/2
723.3 eV
Sat.
715.2 eV
Fe 2p3/2
710.2 eV
e
CF3-MIL-101(Fe) before
Fe 2p
Fe 2p1/2
723.8 eV
Sat.
715.6 eV
Fe 2p3/2
710.7 eV
CF3-MIL-101(Fe) after
Fe 2p
Fe 2p1/2
722.8 eV
Fe 2p3/2
709.7 eV
f
NH2-MIL-101(Fe) before
Fe 2p
Fe 2p1/2
723.3 eV
Sat.
715.2 eV
Fe 2p3/2
710.2 eV
NH2-MIL-101(Fe) after
Fe 2p
Fe 2p1/2
724.1 eV
Fe 2p3/2
711 eV

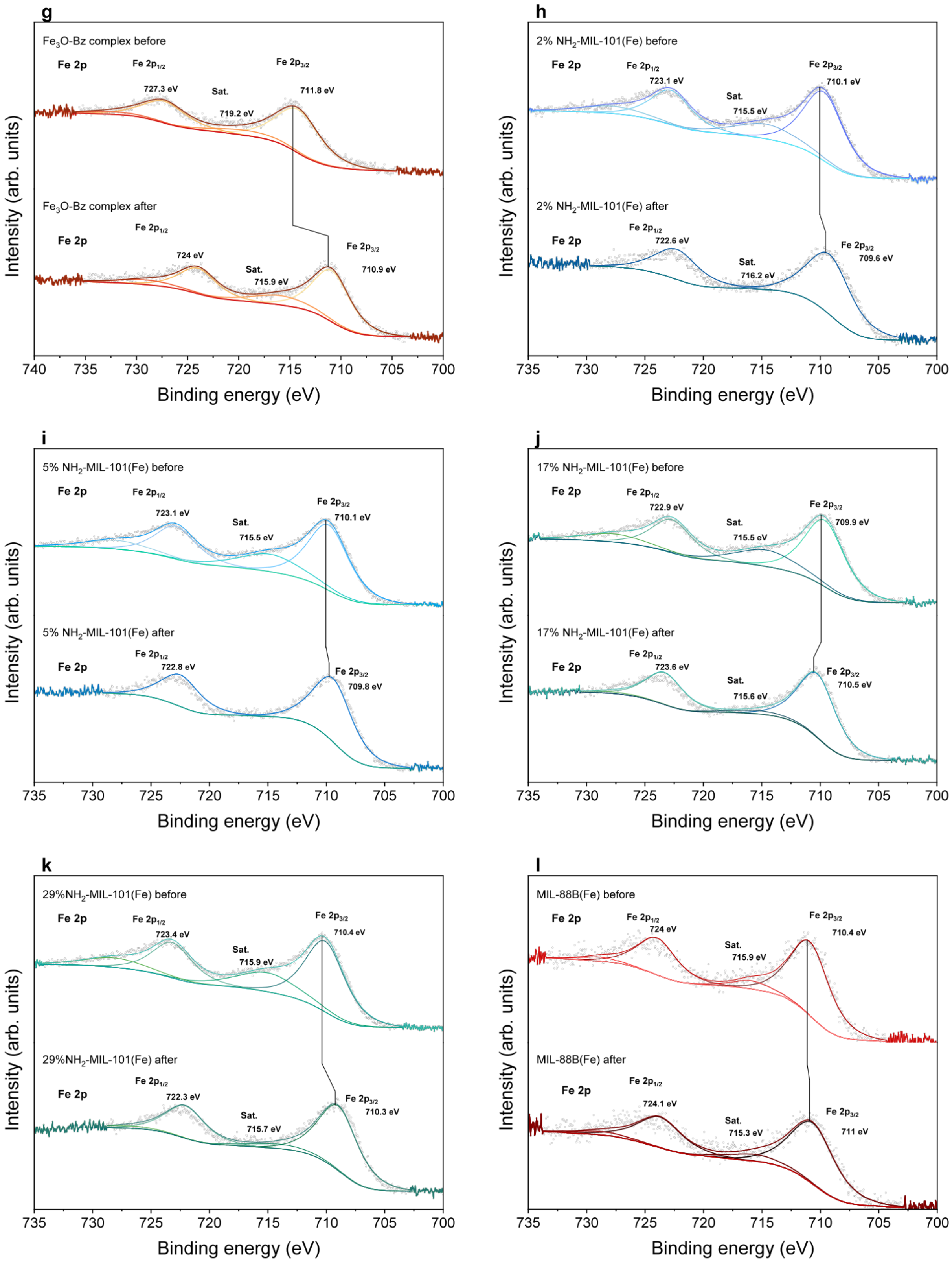
g
Fe3O-Bz complex before
Fe 2p
Fe 2p1/2
727.3 eV
Sat.
719.2 eV
Fe 2p3/2
711.8 eV
Fe3O-Bz complex after
Fe 2p
Fe 2p1/2
724 eV
Sat.
715.9 eV
Fe 2p3/2
710.9 eV
Intensity (arb. units)
Binding energy (eV)
h
2% NH2-MIL-101(Fe) before
Fe 2p
Fe 2p1/2
723.1 eV
Sat.
715.5 eV
Fe 2p3/2
710.1 eV
2% NH2-MIL-101(Fe) after
Fe 2p
Fe 2p1/2
722.6 eV
Sat.
716.2 eV
Fe 2p3/2
709.6 eV
Intensity (arb. units)
Binding energy (eV)
i
5% NH2-MIL-101(Fe) before
Fe 2p
Fe 2p1/2
723.1 eV
Sat.
715.5 eV
Fe 2p3/2
710.1 eV
5% NH2-MIL-101(Fe) after
Fe 2p
Fe 2p1/2
722.8 eV
Fe 2p3/2
709.8 eV
Intensity (arb. units)
Binding energy (eV)
j
17% NH2-MIL-101(Fe) before
Fe 2p
Fe 2p1/2
722.9 eV
Sat.
715.5 eV
Fe 2p3/2
709.9 eV
17% NH2-MIL-101(Fe) after
Fe 2p
Fe 2p1/2
723.6 eV
Sat.
715.6 eV
Fe 2p3/2
710.5 eV
Intensity (arb. units)
Binding energy (eV)
k
29%NH2-MIL-101(Fe) before
Fe 2p
Fe 2p1/2
723.4 eV
Sat.
715.9 eV
Fe 2p3/2
710.4 eV
29%NH2-MIL-101(Fe) after
Fe 2p
Fe 2p1/2
722.3 eV
Sat.
715.7 eV
Fe 2p3/2
710.3 eV
Intensity (arb. units)
Binding energy (eV)
l
MIL-88B(Fe) before
Fe 2p
Fe 2p1/2
724 eV
Sat.
715.9 eV
Fe 2p3/2
710.4 eV
MIL-88B(Fe) after
Fe 2p
Fe 2p1/2
724.1 eV
Sat.
715.3 eV
Fe 2p3/2
711 eV
Intensity (arb. units)
Binding energy (eV)

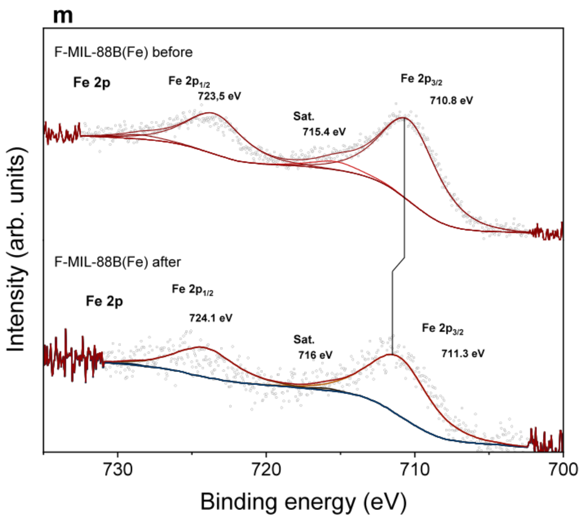

Figure S7. XPS spectra of the Fe 2p region of MIL-101(Fe), MIL-88B(Fe), their functionalized derivatives, and a Fe_3O-based complex before and after photocatalysis. Samples are shown in the same order as in Figure S6.

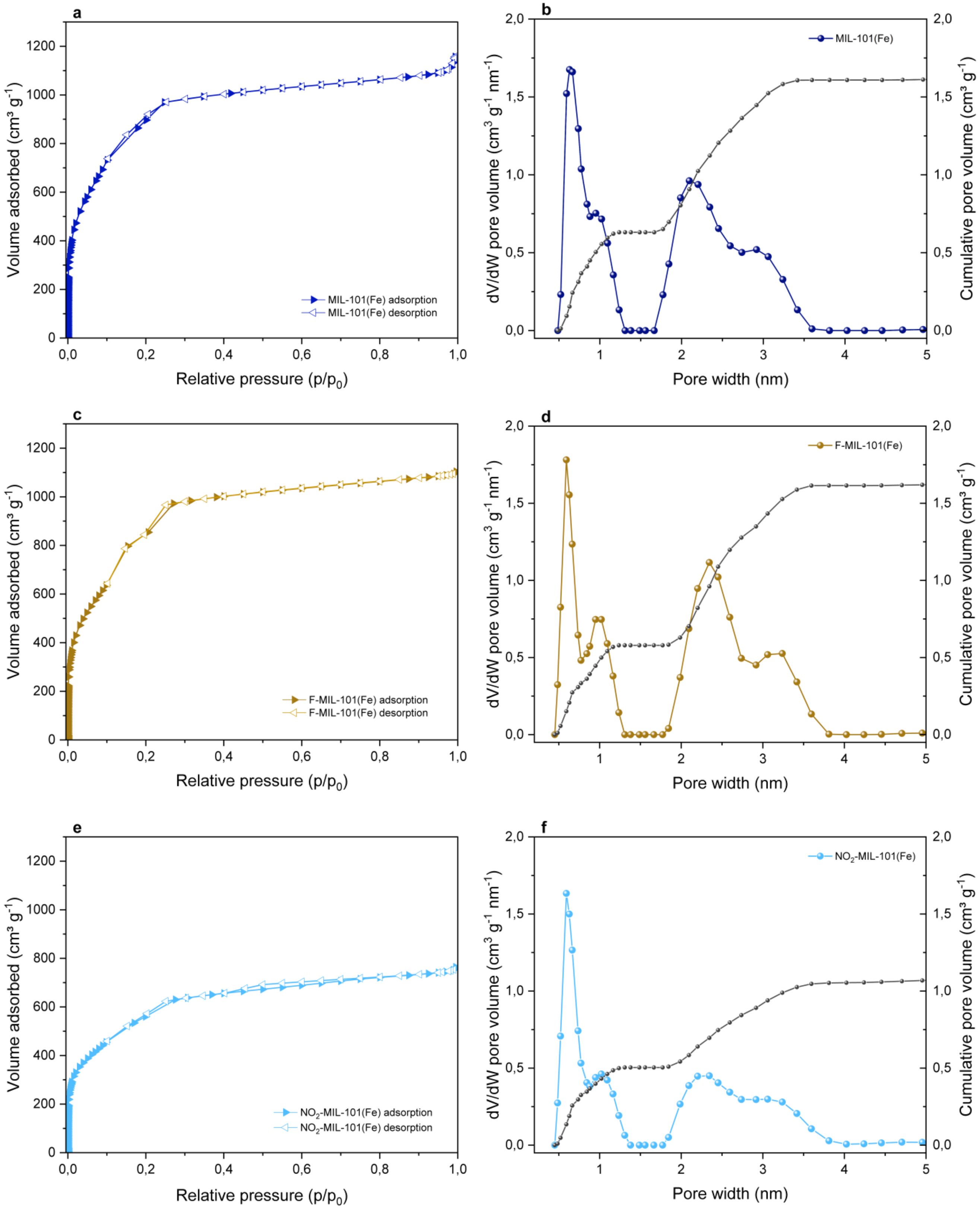
a
Volume adsorbed (cm³ g⁻¹)
Relative pressure (p/p0)
MIL-101(Fe) adsorption
MIL-101(Fe) desorption
b
dV/dW pore volume (cm³ g⁻¹ nm⁻¹)
Cumulative pore volume (cm³ g⁻¹)
Pore width (nm)
MIL-101(Fe)
c
Volume adsorbed (cm³ g⁻¹)
Relative pressure (p/p0)
F-MIL-101(Fe) adsorption
F-MIL-101(Fe) desorption
d
dV/dW pore volume (cm³ g⁻¹ nm⁻¹)
Cumulative pore volume (cm³ g⁻¹)
Pore width (nm)
F-MIL-101(Fe)
e
Volume adsorbed (cm³ g⁻¹)
Relative pressure (p/p0)
NO2-MIL-101(Fe) adsorption
NO2-MIL-101(Fe) desorption
f
dV/dW pore volume (cm³ g⁻¹ nm⁻¹)
Cumulative pore volume (cm³ g⁻¹)
Pore width (nm)
NO2-MIL-101(Fe)

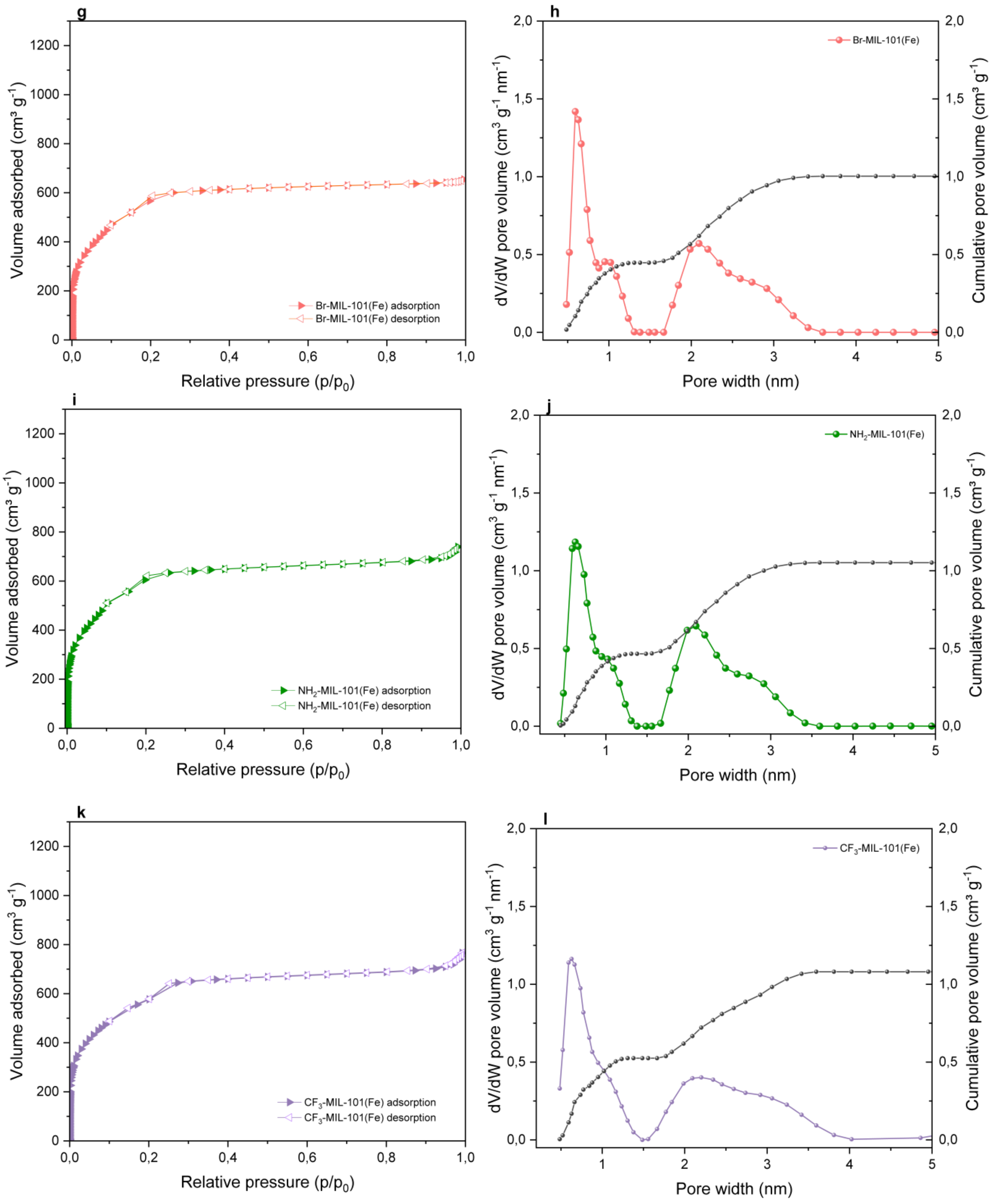
g
Volume adsorbed (cm³ g⁻¹)
Relative pressure (p/p0)
Br-MIL-101(Fe) adsorption
Br-MIL-101(Fe) desorption
h
dV/dW pore volume (cm³ g⁻¹ nm⁻¹)
Cumulative pore volume (cm³ g⁻¹)
Pore width (nm)
Br-MIL-101(Fe)
i
Volume adsorbed (cm³ g⁻¹)
Relative pressure (p/p0)
NH2-MIL-101(Fe) adsorption
NH2-MIL-101(Fe) desorption
j
dV/dW pore volume (cm³ g⁻¹ nm⁻¹)
Cumulative pore volume (cm³ g⁻¹)
Pore width (nm)
NH2-MIL-101(Fe)
k
Volume adsorbed (cm³ g⁻¹)
Relative pressure (p/p0)
CF3-MIL-101(Fe) adsorption
CF3-MIL-101(Fe) desorption
l
dV/dW pore volume (cm³ g⁻¹ nm⁻¹)
Cumulative pore volume (cm³ g⁻¹)
Pore width (nm)
CF3-MIL-101(Fe)

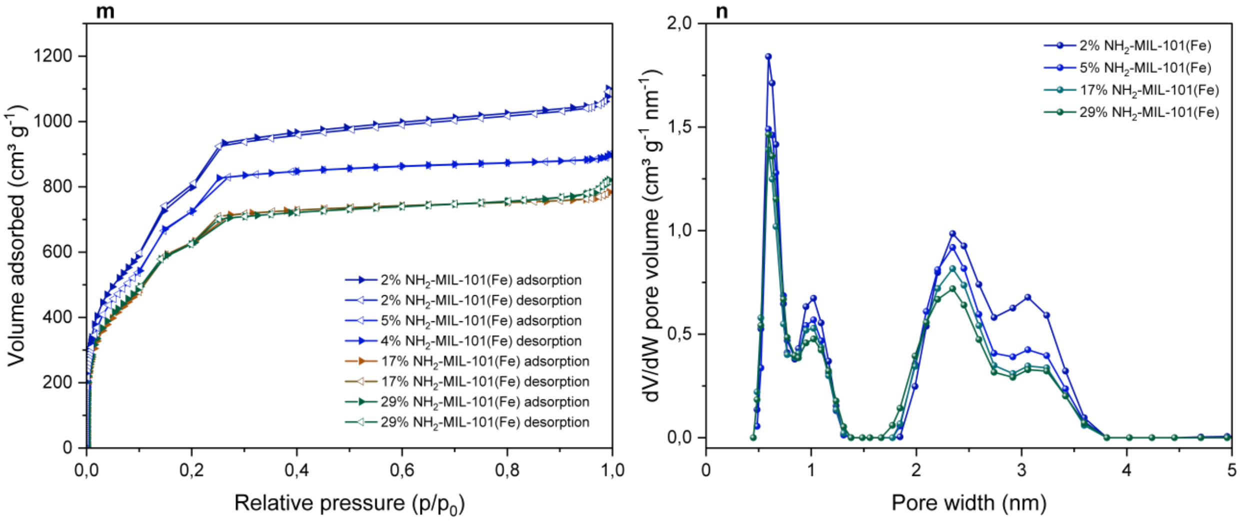

Figure S8. N_2 physisorption isotherms of MIL-101(Fe) and functionalized variants at 77 K are shown in panels (a), (c), (e), (g), (i), (k), and (m); the corresponding NLDFT pore size distributions (derived from the adsorption branch) and cumulative pore volumes are presented in panels (b), (d), (f), (h), (j), (l), and (n).

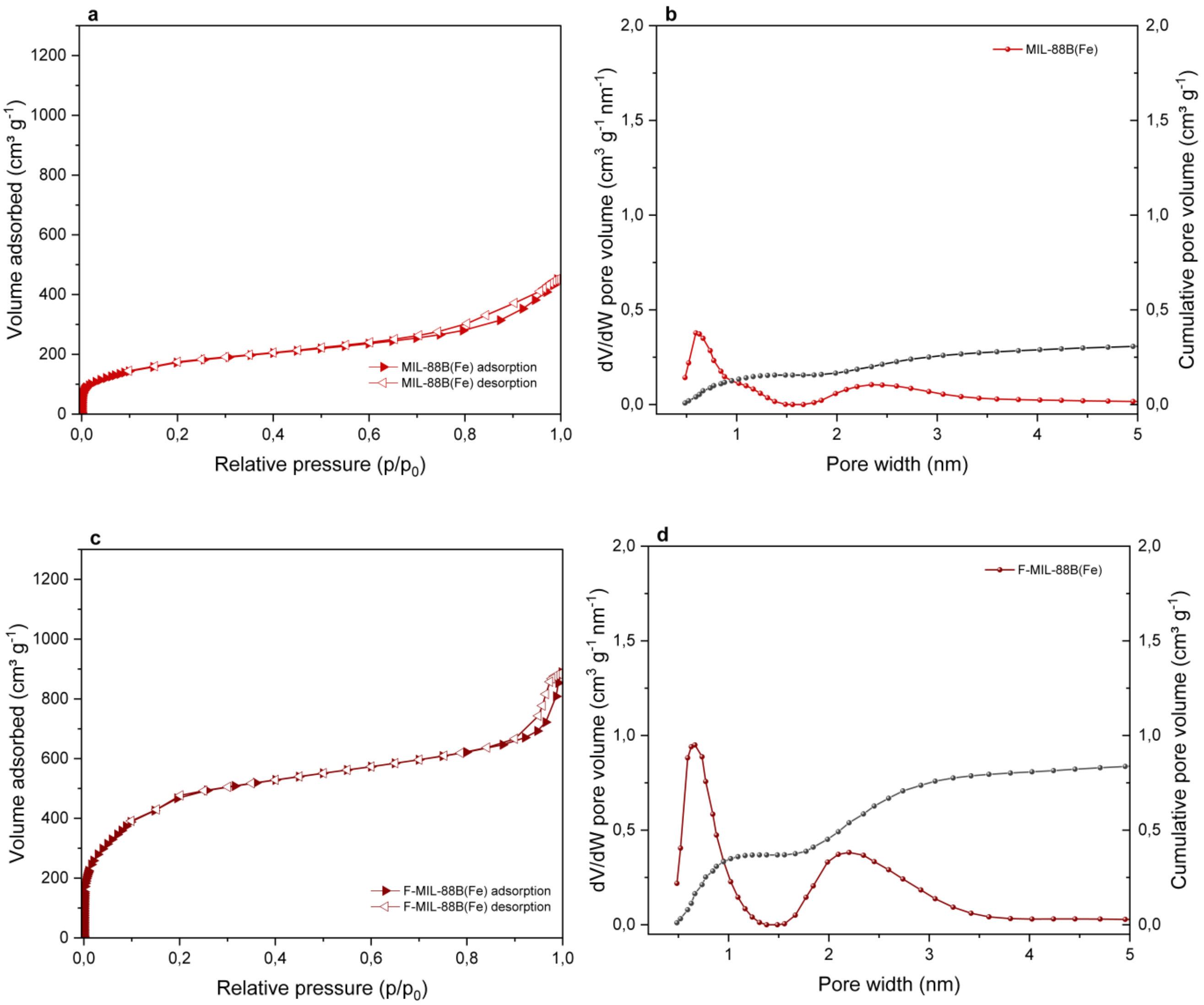

Figure S9. N_2 physisorption isotherms at 77 K for MIL-88B(Fe) and F-MIL-88B(Fe) are shown in panels (a) and (c), with the corresponding NLDFT pore size distributions (from the adsorption branch) and cumulative pore volumes in panels (b) and (d).

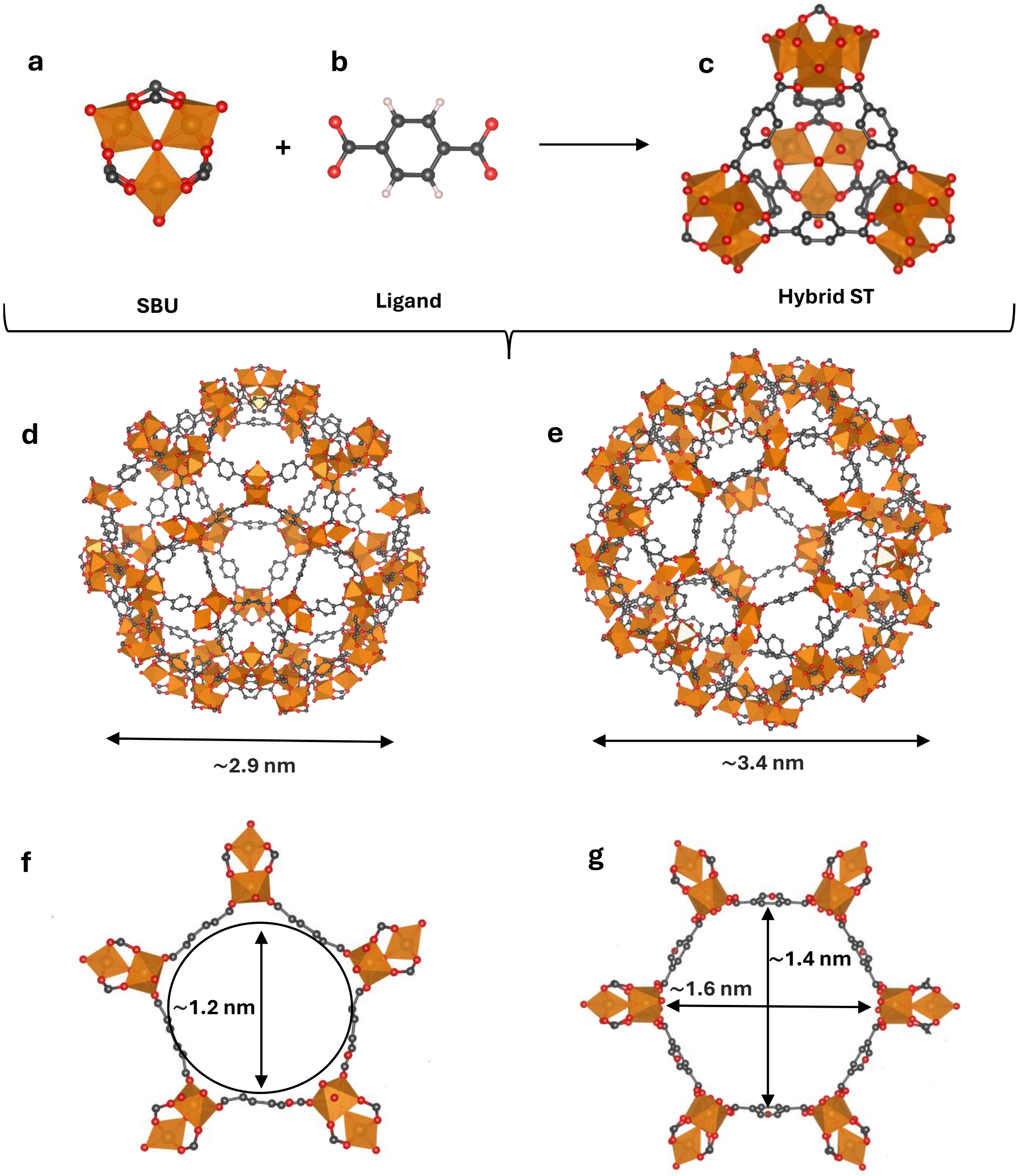

Figure S10. Schematic representation of MIL-101 (Fe) MOF: a) trinuclear secondary building unit bridged by six carboxylic functions; b) terephthalic acid ligand, which lies at c) edges of the super tetrahedron ST, d) smaller cage with f) pentagonal window, e) larger cage with g) window. All structures were drawn in VESTA Software.

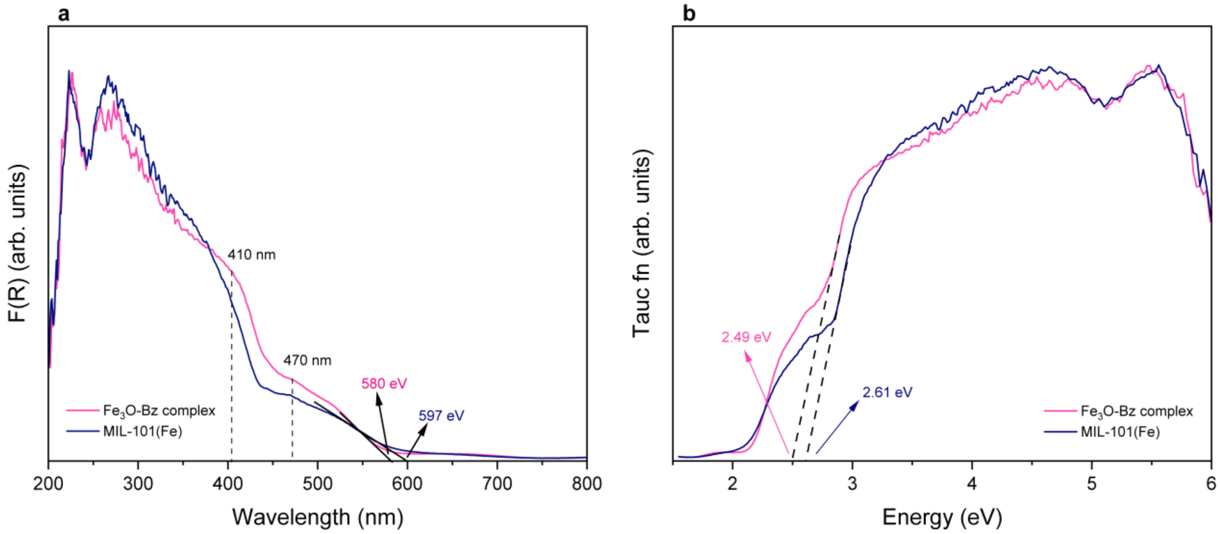

Figure S11. (a) DRS spectrum of the Fe_3O–Bz complex compared to MIL-101(Fe). Marked electronic transitions include a strong band at 410 nm, assigned to ligand-to-metal charge transfer (O^{2-} 2p → Fe^{3+} 3d), and a weaker band at 470 nm, corresponding to the nominally spin-forbidden d–d transition ($^6A_1g \rightarrow {}^4T_1g(G)$) of Fe^{3+} [15,16,17,18]. Although spin-forbidden by strict selection rules, this transition is observed due to spin–orbit coupling and vibronic relaxation within the slightly distorted Fe_3O cluster[19]. (b) Corresponding Tauc plots assuming a direct bandgap, from which the optical bandgap energies were estimated.

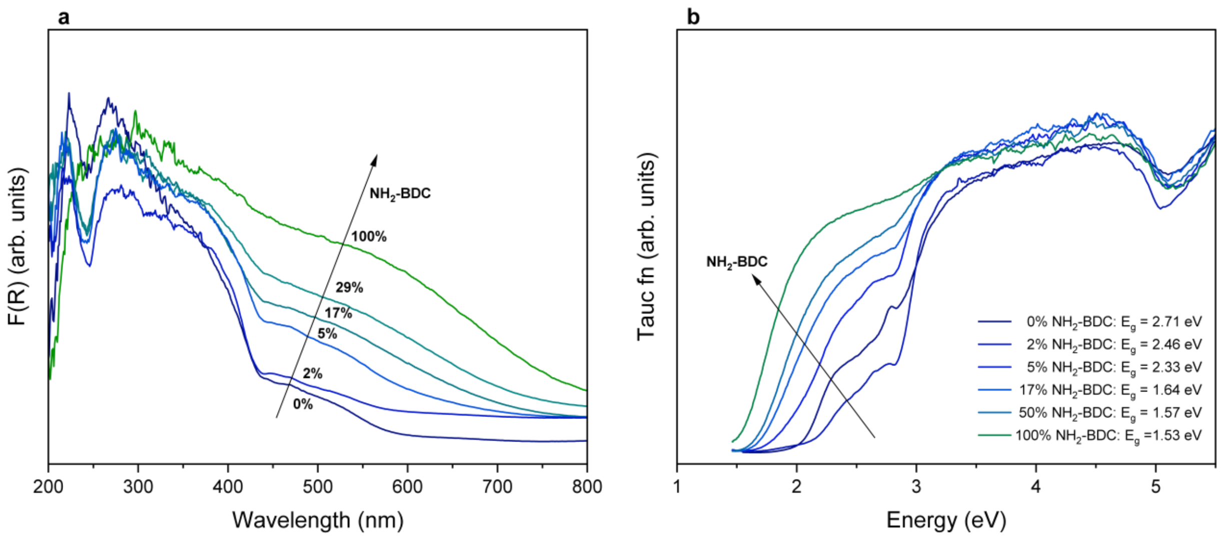

Figure S12. (a) DRS spectra of mixed-ligand xNH2-MIL-101(Fe) MOFs (b) Corresponding Tauc plots derived from DRS data with assumption of a direct bandgap, showing optical bandgap narrowing with increasing NH_2-BDC ligand content. The bandgap decreases from 2.74 eV for MIL-101(Fe) to 1.53 eV for NH_2-MIL-101(Fe) (values indicated in the inset). The progressive redshift and enhanced absorption are attributed to the interaction between the aromatic carbon of the BDC ligand and the nitrogen of the NH_2 group, forming π-bonding interactions, along with the N-H···C hydrogen bonding resulting with the mid-gap state[20]. Increasing NH_2-BDC content leads to progressive absorbance enhancement as more electronic states evolve.

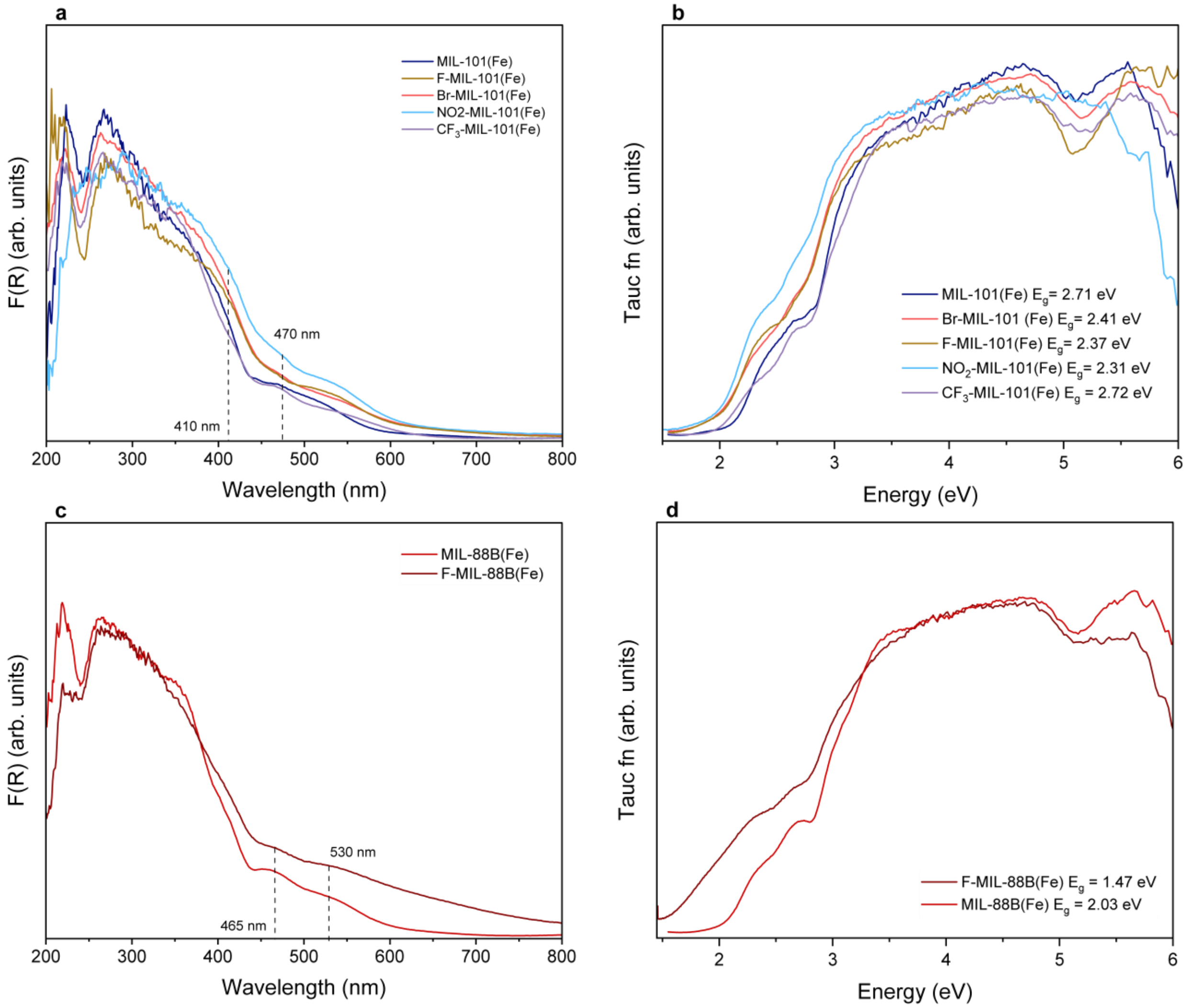

Figure S13. (a) Diffuse reflectance spectra (DRS) of single-ligand MIL-101(Fe) derivatives: pristine (unfunctionalized), NO_2-, Br-, F-, and CF_3-functionalized samples. (b) Corresponding Tauc plots used to determine optical bandgaps. All functionalized MOFs show the same electronic transitions as pristine MIL-101(Fe), while ligand substitution modulates the absorbance, as discussed in the main text. (c) DRS spectra of MIL-88B(Fe) and F-MIL-88B(Fe), showing electronic transitions red-shifted relative to MIL-101(Fe), consistent with literature[21], The F-functionalized MIL-88B(Fe) shows increased absorbance compared to the unfunctionalized sample. (d) Tauc plots of MIL-88B(Fe) and F-MIL-88B(Fe), reflecting significantly smaller optical bandgaps for MIL-88B(Fe) compared to MIL-101(Fe) due to the red shift, and an even lower optical bandgap for the F-functionalized variant due to its higher absorbance.

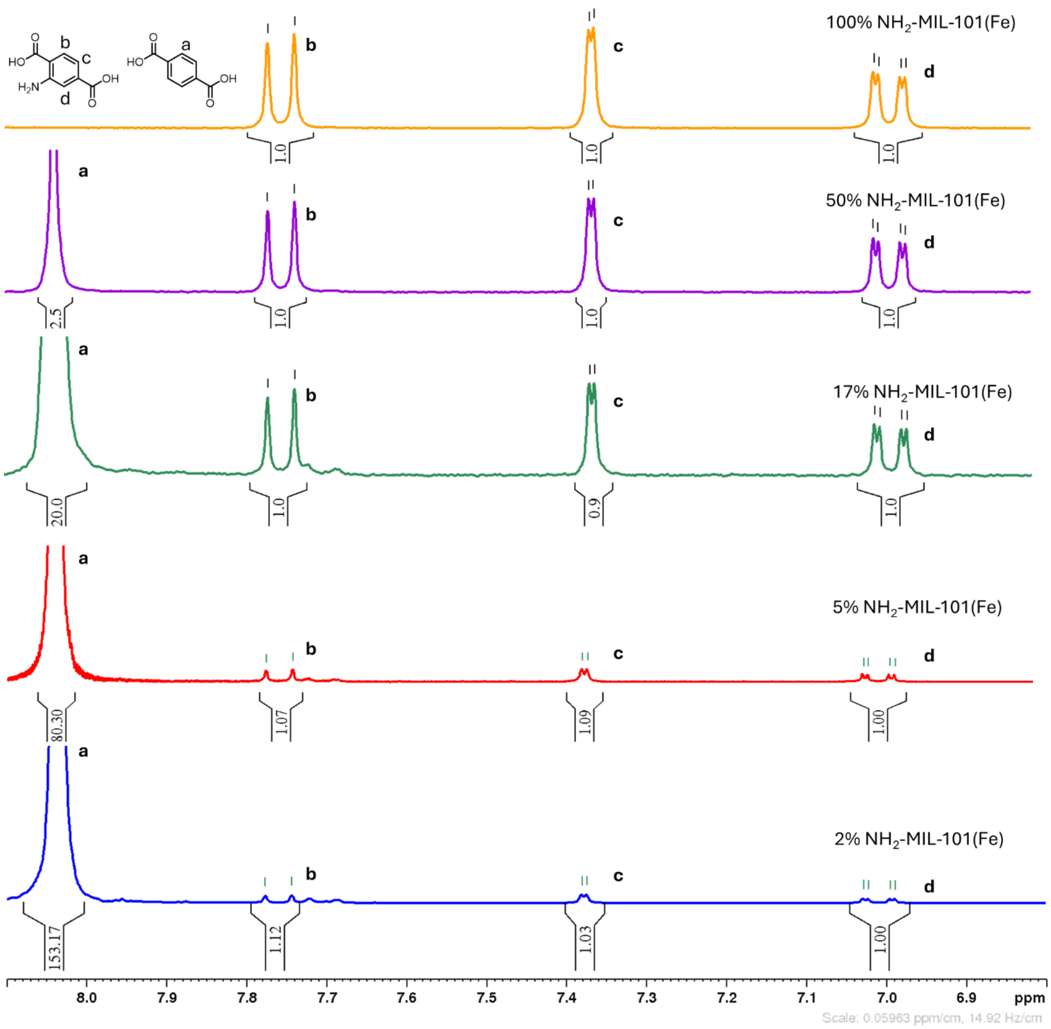

Figure S14. ^{1}H-NMR spectroscopic information for mixed-ligand MOFs. **2 mol.% NH_2-BDC:** *^{1}H-NMR (250 Hz, d_6-DMSO) δ 6.98 -7.02 ppm (dd, 1H J = 8.29 Hz, J = 1.26 Hz), ^{1}H-NMR (250 Hz, d_6-DMSO) δ 7.36 -7.38 ppm (d,1H J= 1.14); ^{1}H-NMR (250 Hz, d_6-DMSO) δ 7.72-7.74 ppm (d,1H, J=8.27 Hz), ^{1}H-NMR (250 Hz, d_6-DMSO) δ 8.03 ppm (s, 4H).* **5 mol.% NH_2-BDC**: *^{1}H-NMR (250 Hz, d_6-DMSO) δ 6.98 -7.02 ppm (dd, 1H, ^3J = 8.29 Hz, ^4J = 1.26Hz), ^{1}H-NMR (250 Hz, d_6-DMSO) δ 7.36 -7.38 ppm (d,1H, ^2J=1.14 Hz), ^{1}H-NMR (250 Hz, d_6-DMSO) δ 7.73 - 7.76 ppm (d, 1H, J= 8.27 Hz), 8.03 ppm (s,4H).* **17 mol.% NH_2-BDC**: *^{1}H-NMR (250 Hz, d_6-DMSO) δ 7.20 - 7.24 ppm (dd, 1H, J = 8.37 Hz, J = 1.82 Hz), ^{1}H-NMR (250 Hz, d_6-DMSO) δ 7.40 - 7.41, (d 1H ^2J=1.57 Hz), ^{1}H-NMR (250 Hz, d_6-DMSO) δ 7.82 - 7.85 ppm (d, 1H, J=8.32 Hz), ^{1}H-NMR (250 Hz, d_6-DMSO) δ 7.99 ppm (s,4H).* **50 mol.% NH_2-BDC**: *^{1}H-NMR (250 Hz, d_6-DMSO) δ 7.96 - 7. 39ppm (dd, 1H, J = 8.46 Hz, J = 1.10Hz), ^{1}H-NMR (250 Hz, d_6-DMSO) δ 7.38 - 7-39 ppm (d, 1H, J= 1.48 Hz), δ 7.81 - 7.84 ppm (d,1H, J= 8.23 Hz), ^{1}H-NMR (250 Hz, d6-DMSO) δ 7.98 ppm (s,4H).* **100 mol.% NH_2-BDC**: *^{1}H-NMR (250 Hz, d_6-DMSO) δ 6.98 - 7.02 ppm (dd, J = 8.29 Hz, J = 1.53 Hz); ^{1}H-NMR (250 Hz, d_6 -DMSO) δ 7.36 -7.38 ppm (d, 1H, J =1.56 Hz); ^{1}H-NMR (250 Hz, d_6-DMSO) δ 7.73 - 7-76 ppm (d, 1H, J = 8. 29 Hz).*

S3. PHOTOCATALYTIC ACTIVITY

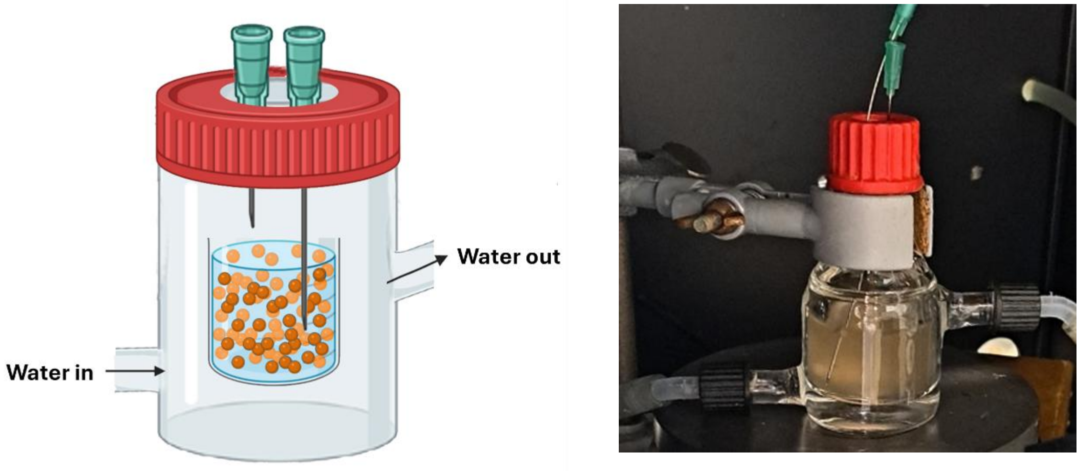

Figure S15. Custom-made water-jacked cooled photoreactor.

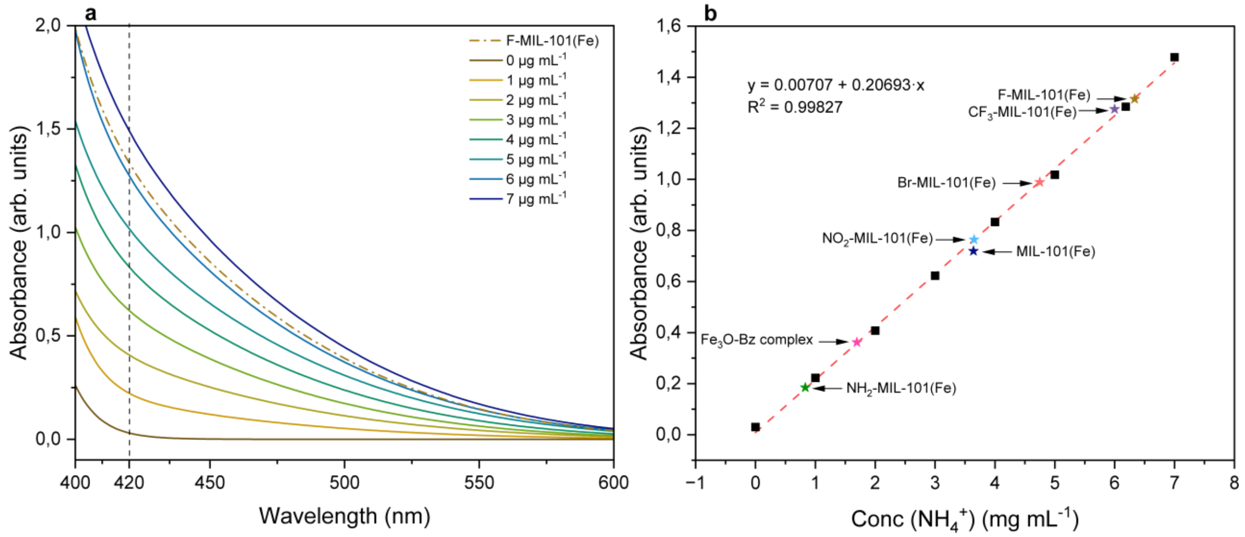

Figure S16. Ammonia detection via Nessler's reagent method: (a) UV-Vis absorption spectra for varying concentrations of NH_4^+ ions after 15 min of incubation with Nessler's reagent. (b) Calibration curve for NH_4^+ concentration estimation, based on the average values of triplicate measurements from separately prepared standards.

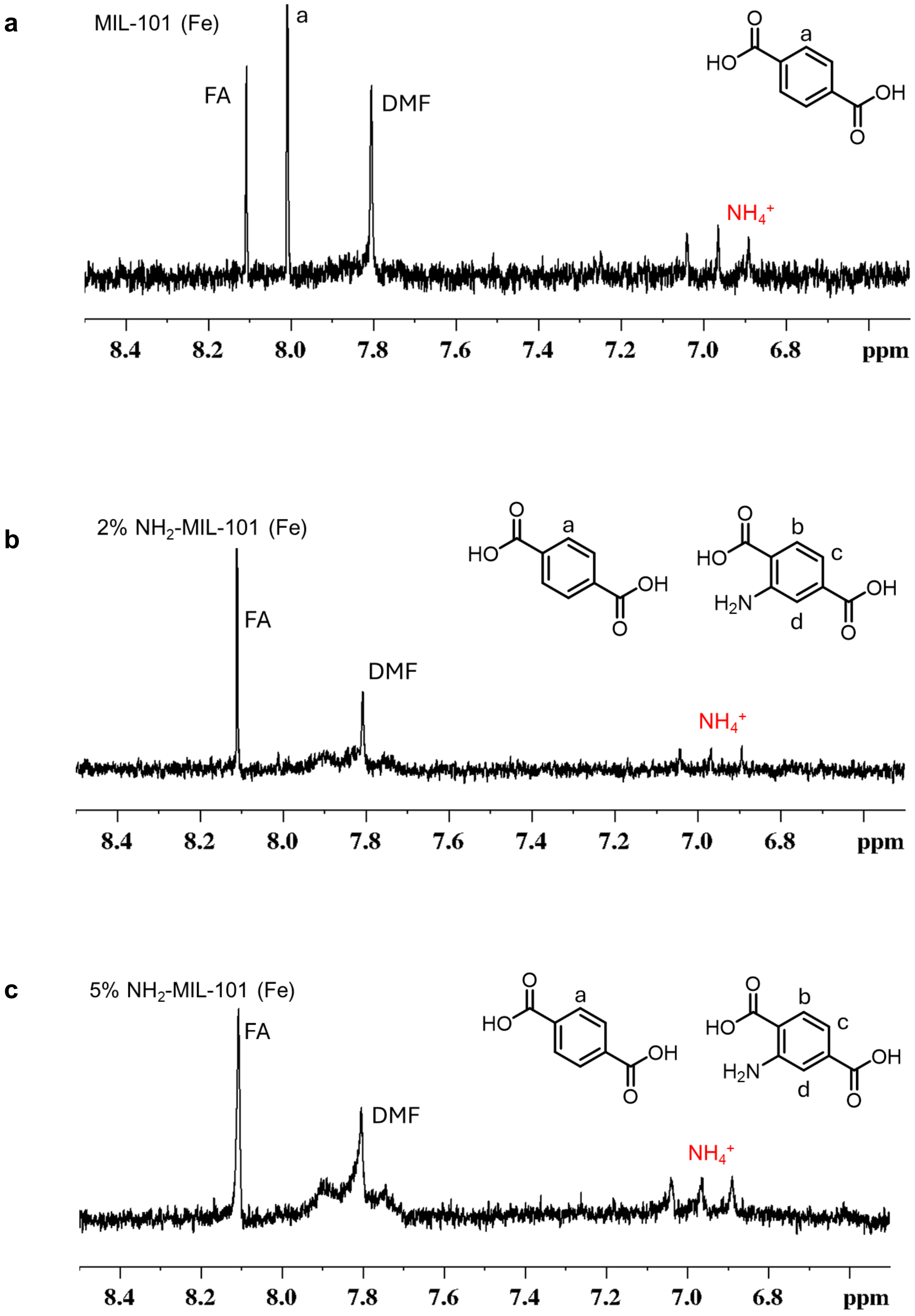
a
MIL-101 (Fe)
a
FA
DMF
NH4+
8.4
8.2
8.0
7.8
7.6
7.4
7.2
7.0
6.8
ppm
HO
OH
b
2% NH2-MIL-101 (Fe)
FA
DMF
NH4+
b
c
d
H2N
c
5% NH2-MIL-101 (Fe)
FA
DMF
NH4+

d

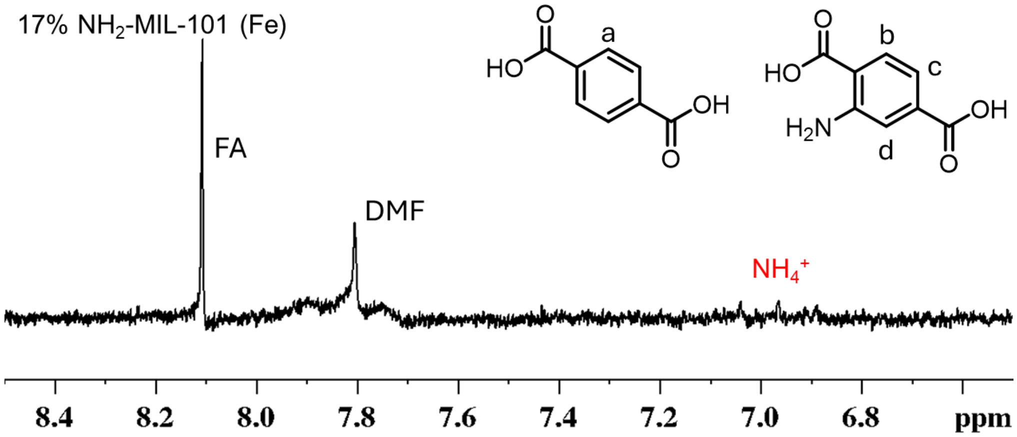
17% NH2-MIL-101 (Fe)
a
b
c
HO
OH
HO
H2N
d
OH
FA
DMF
NH4+
8.4
8.2
8.0
7.8
7.6
7.4
7.2
7.0
6.8
ppm

e

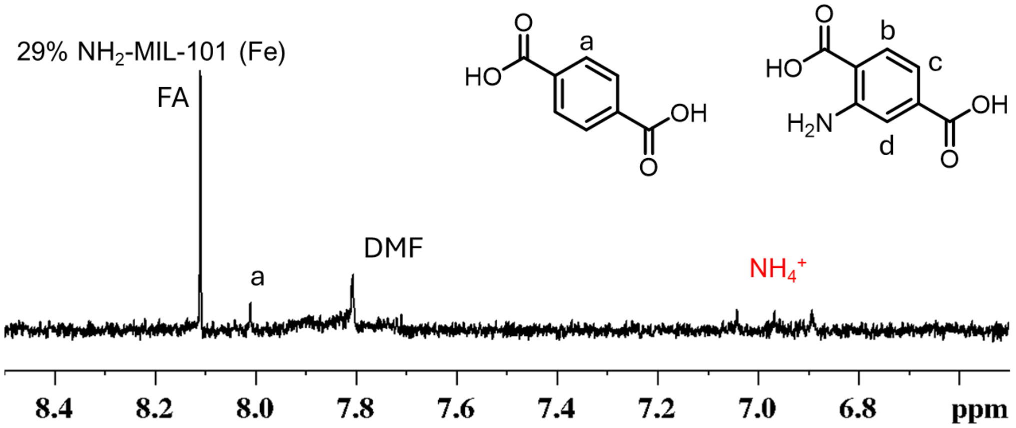
29% NH2-MIL-101 (Fe)
FA
a
b
c
HO
OH
H2N
d
DMF
a
NH4+
8.4
8.2
8.0
7.8
7.6
7.4
7.2
7.0
6.8
ppm

f

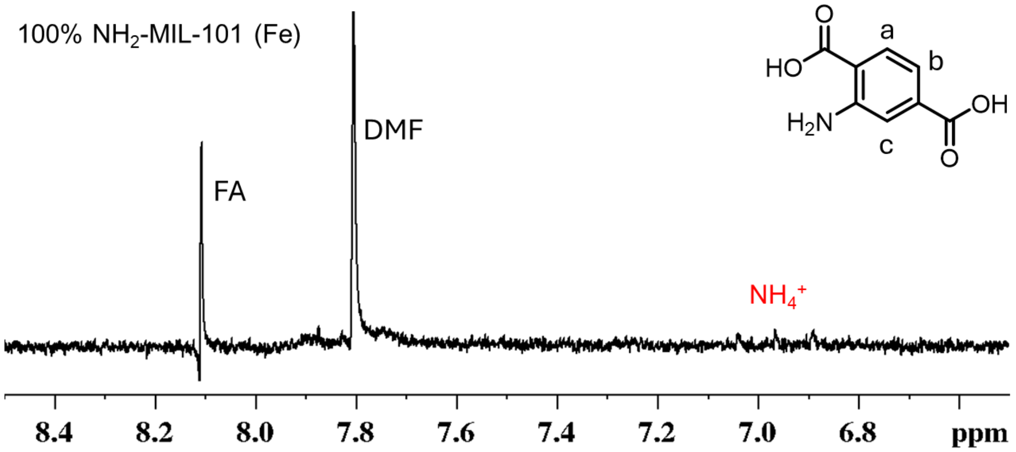
100% NH2-MIL-101 (Fe)
a
b
HO
H2N
OH
c
DMF
FA
NH4+
8.4
8.2
8.0
7.8
7.6
7.4
7.2
7.0
6.8
ppm

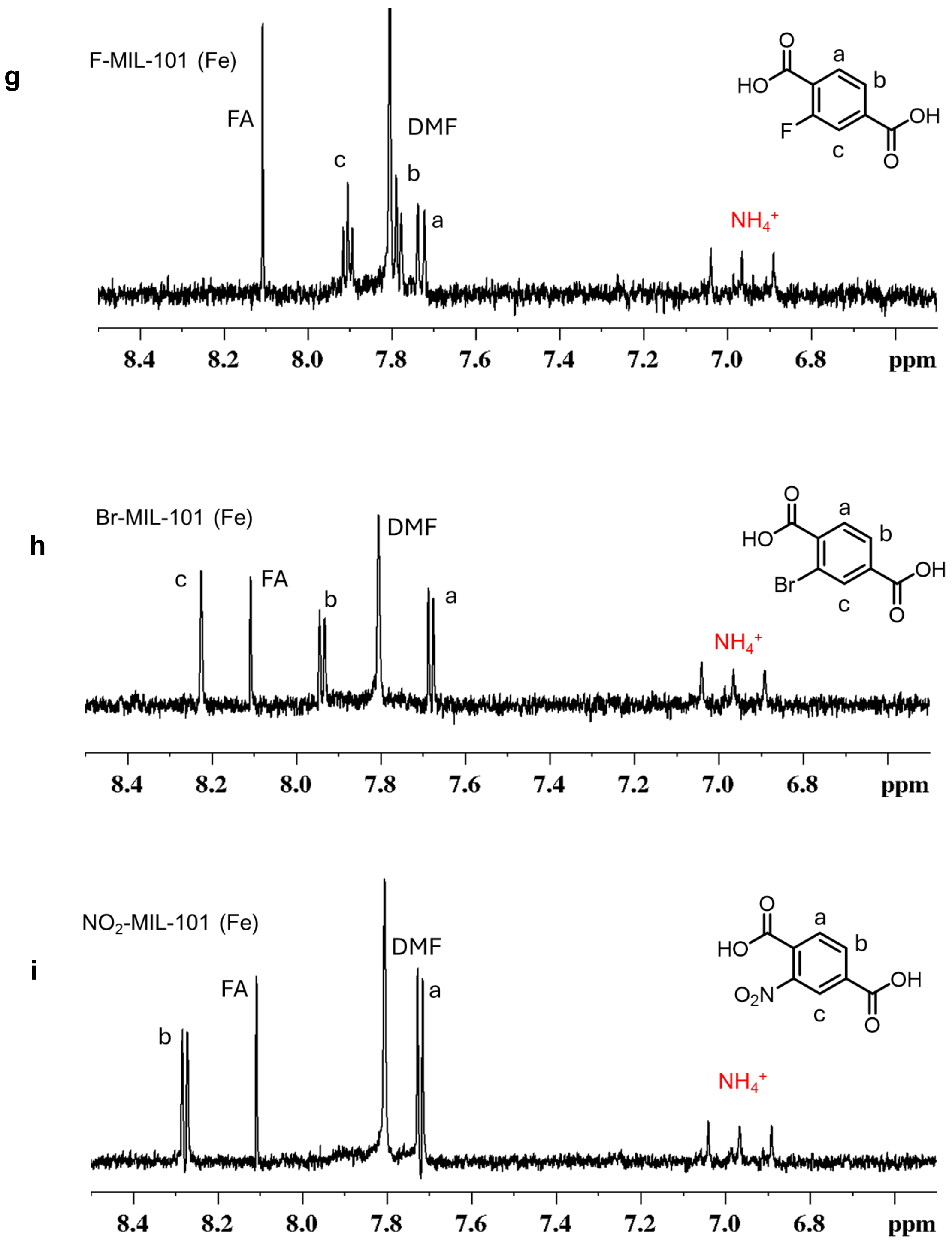

Figure S17. ^{1}H-NMR spectra of supernatant after photocatalytic NH_3 synthesis for 1h: (a) MIL-101(Fe). (b) 2%NH_2-MIL-101(Fe). (c) 5%NH_2-MIL-101(Fe). (d) 17%NH_2-MIL-101(Fe). (e) 29%NH_2-MIL-101(Fe), (f) NH_2-MIL-101(Fe), (g) F-MIL-101(Fe), (h) Br-MIL-101(Fe), (i) NO_2-MIL-101(Fe).

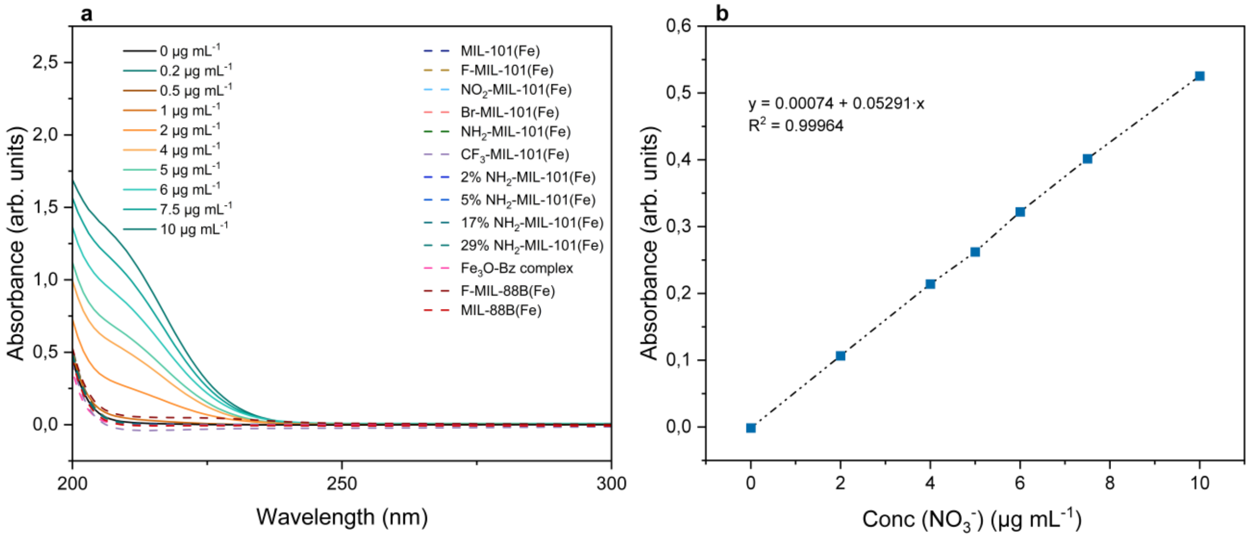

Figure S18. (a) UV-Vis absorption spectra of various NO_3^- concentrations after incubation for 10 min and (b) the corresponding standard curve for NO_3^- detection.

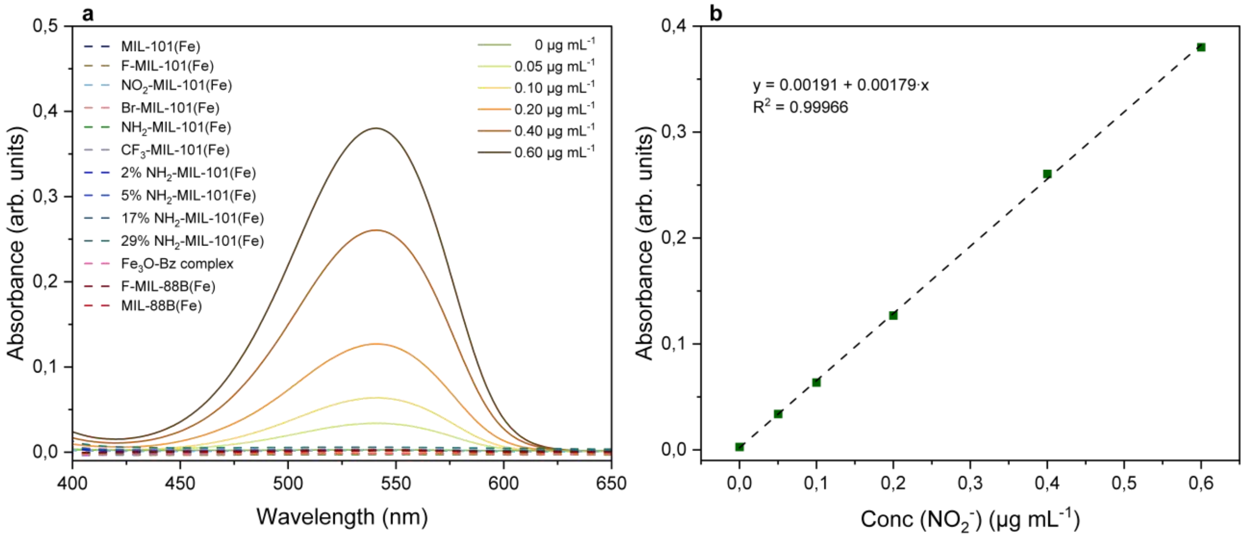

Figure S19. (a) UV-Vis absorption spectra of various NO_2^- concentrations after incubation for 20 min and (b) the corresponding standard curve for NO_2^- detection.

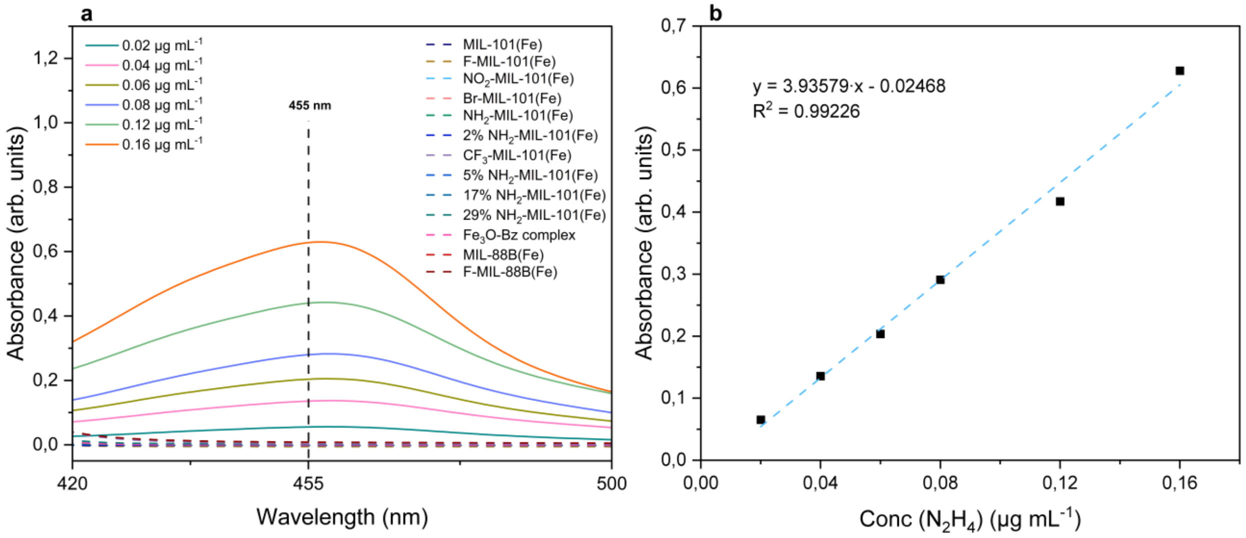

Figure S20. (a) UV–Vis spectra of N_2H_4 standards and reaction solutions from F-, Br-, NO_2-, and non-functionalized MIL-101(Fe) after 20 min incubation. (b) Calibration plot for N_2H_4 determination.

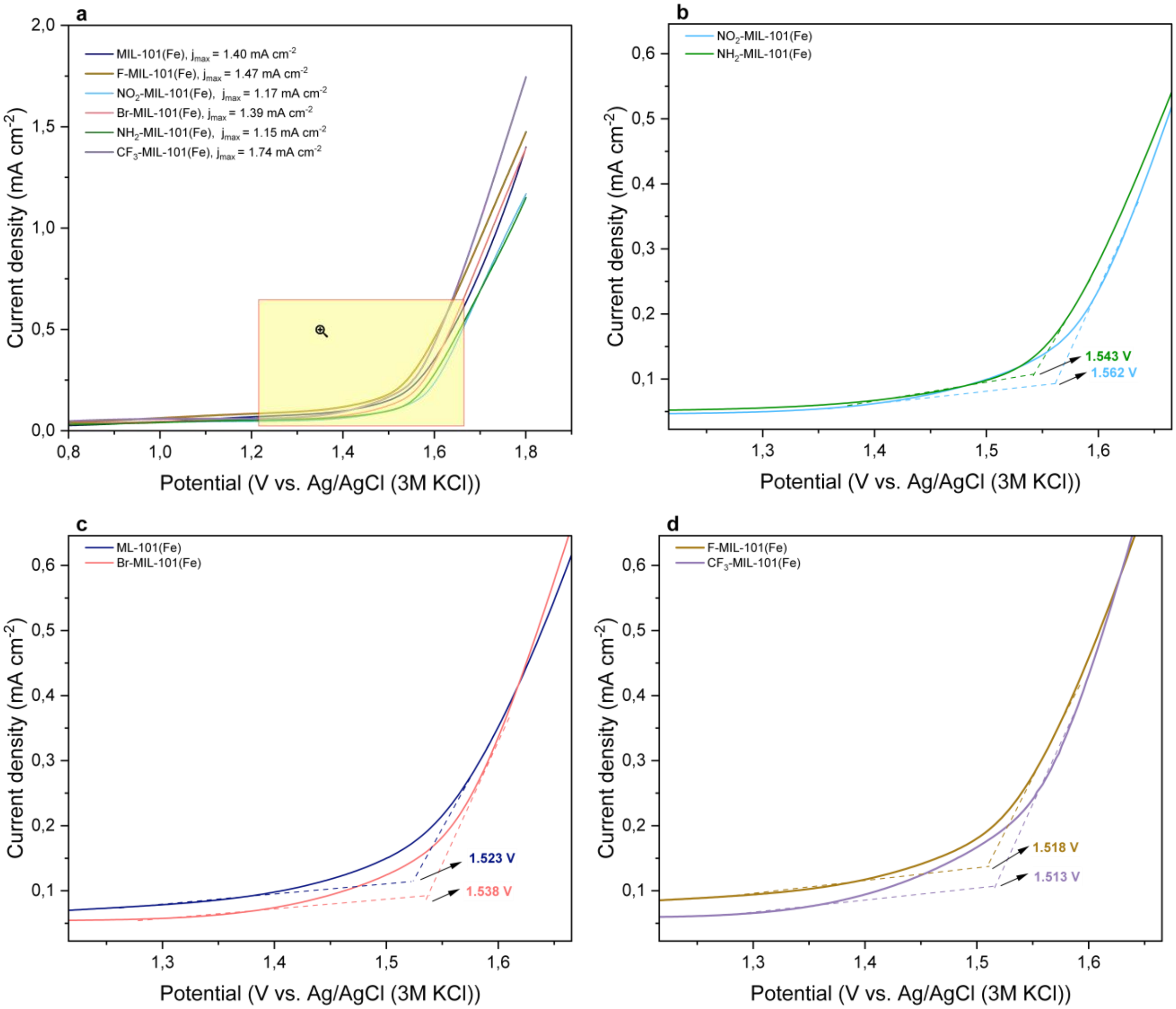

Figure S21. Linear sweep voltammograms (J–V curves) of all single-ligand MOFs measured in 0.1 M Na_2SO_4 electrolyte vs Ag/AgCl (3 M KCl): (a) full range showing the maximum current densities; (b) enlarged view of NO_2- and NH_2-functionalized samples; (c) enlarged view of Br- and unfunctionalized MIL-101(Fe); (d) enlarged view of F- and CF_3-MIL-101(Fe).

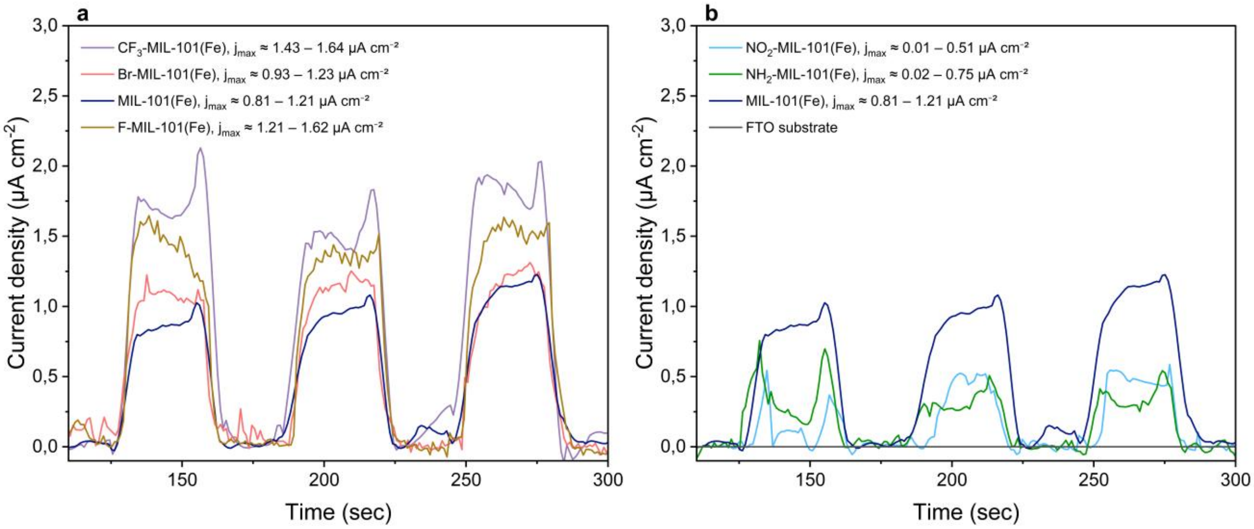

Figure S22. Transient photocurrent responses of single-ligand MOF electrodes under chopped visible light irradiaion ($\lambda \geq 420$ nm, Xe lamp, 23 mW cm^{-2}) at 1.6 V vs Ag/AgCl (3 M KCl) in 0.1 M Na_2SO_4 aqueous solution: (a) CF_3-, F-, Br-, and unfunctionalized MIL-101(Fe) samples, with current densities indicated; (b) samples exhibiting lower photocurrent relative to unfunctionalized MIL-101(Fe), namely NH_2- and NO_2-functionalized variants, along with the bare FTO substrate.

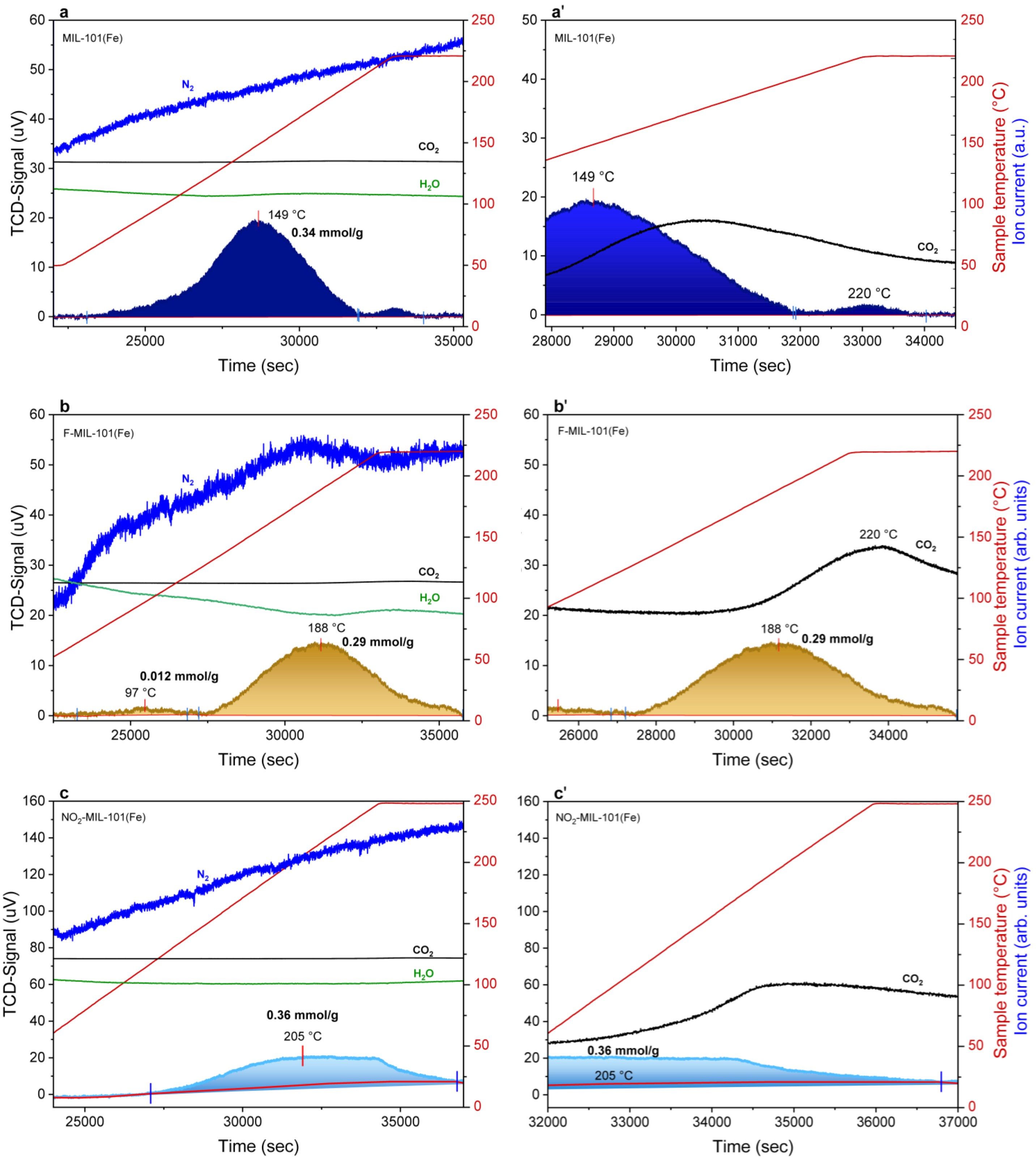
a
MIL-101(Fe)
N2
CO2
H2O
149 °C
0.34 mmol/g
TCD-Signal (uV)
Time (sec)
a'
MIL-101(Fe)
149 °C
220 °C
CO2
Sample temperature (°C)
Ion current (a.u.)
b
F-MIL-101(Fe)
N2
CO2
H2O
188 °C
0.29 mmol/g
0.012 mmol/g
97 °C
b'
F-MIL-101(Fe)
220 °C
CO2
188 °C
0.29 mmol/g
Ion current (arb. units)
c
NO2-MIL-101(Fe)
N2
CO2
H2O
0.36 mmol/g
205 °C
c'
NO2-MIL-101(Fe)
CO2
0.36 mmol/g
205 °C

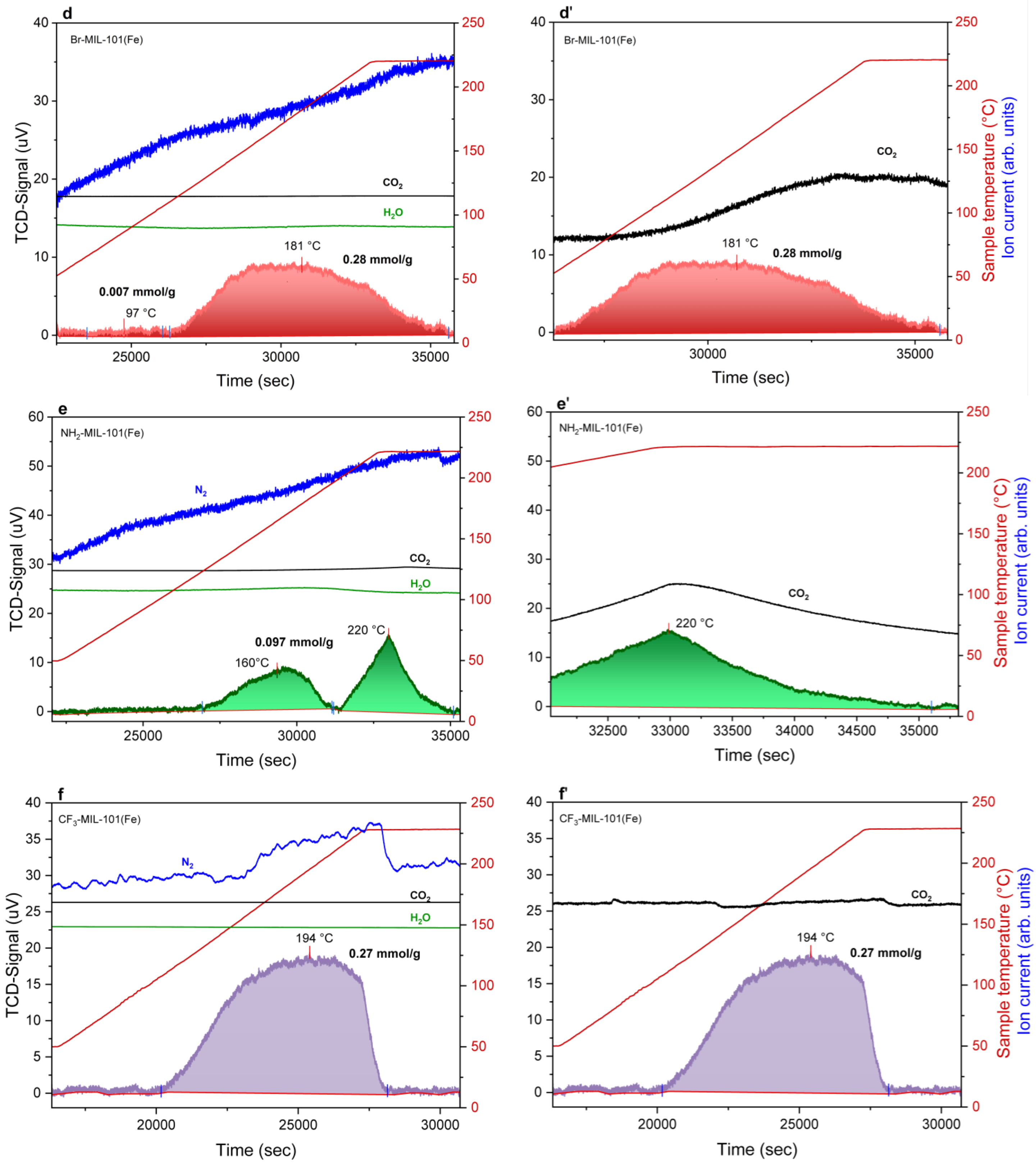
d
Br-MIL-101(Fe)
CO_2
H_2O
181 °C
0.28 mmol/g
0.007 mmol/g
97 °C
d'
Br-MIL-101(Fe)
CO_2
181 °C
0.28 mmol/g
e
NH_2-MIL-101(Fe)
N_2
CO_2
H_2O
220 °C
0.097 mmol/g
160°C
e'
NH_2-MIL-101(Fe)
CO_2
220 °C
f
CF_3-MIL-101(Fe)
N_2
CO_2
H_2O
194 °C
0.27 mmol/g
f'
CF_3-MIL-101(Fe)
CO_2
194 °C
0.27 mmol/g
TCD-Signal (uV)
Time (sec)
Sample temperature (°C)
Ion current (arb. units)

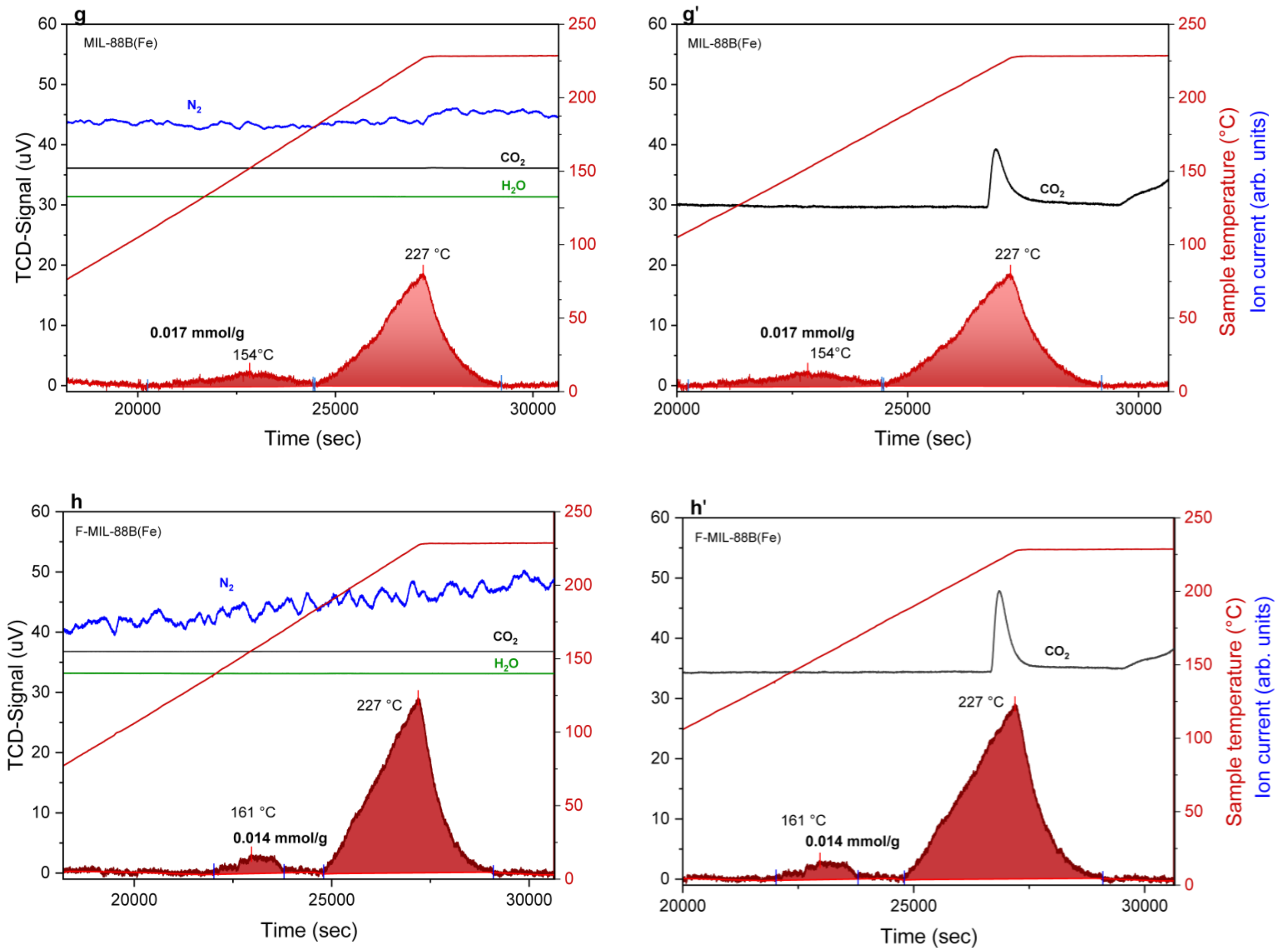

Figure S23. N_2-TPD profiles of as-prepared single-ligand MIL-101(Fe) and MIL-88B(Fe)-based MOFs, recorded at a constant heating rate of 1 °C min^{-1} from 50 to 230 °C, followed by a plateau at 230 °C for 1 h.

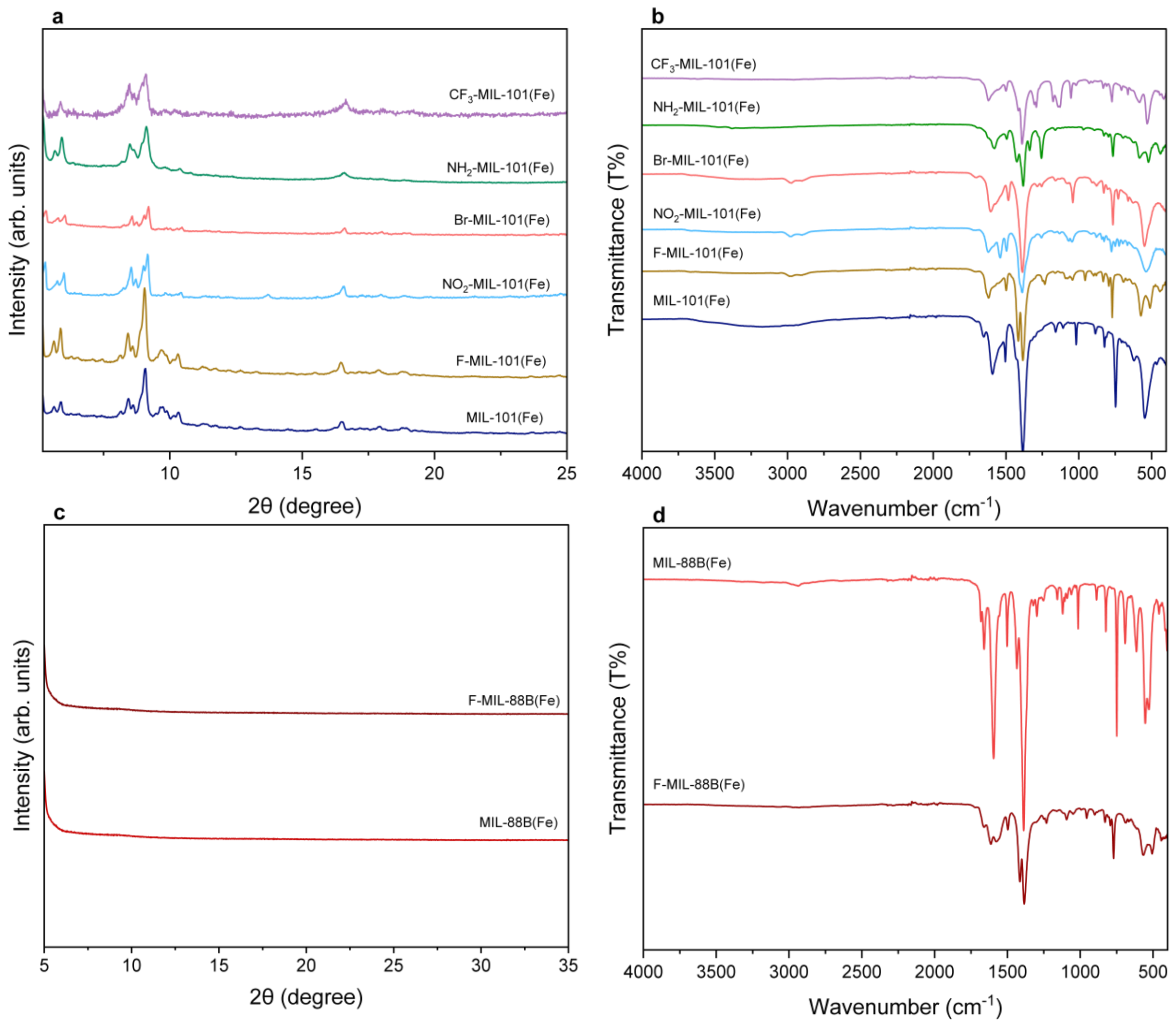

Figure S24. Characterization after N_2-TPD (a) and (c) PXRD patterns. (b and (d) FTIR spectra of all tested samples.

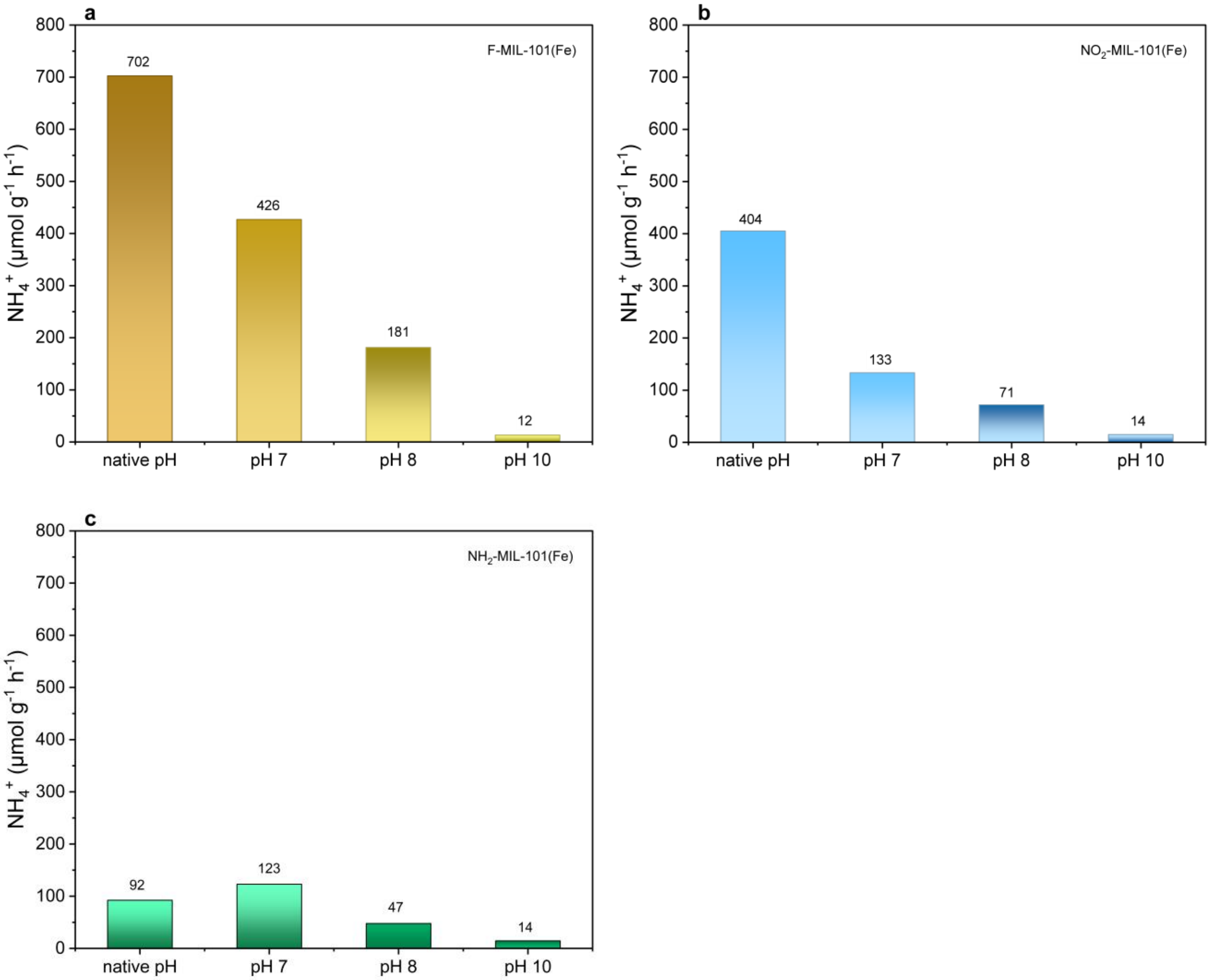

Figure S25. NH_4^+ production yields of MOFs at different initial dispersion pH values (7, 8, 10, and unadjusted pH): (a) F-MIL-101(Fe), (b) NO_2-MIL-101(Fe), and (c) NH_2-MIL-101(Fe).

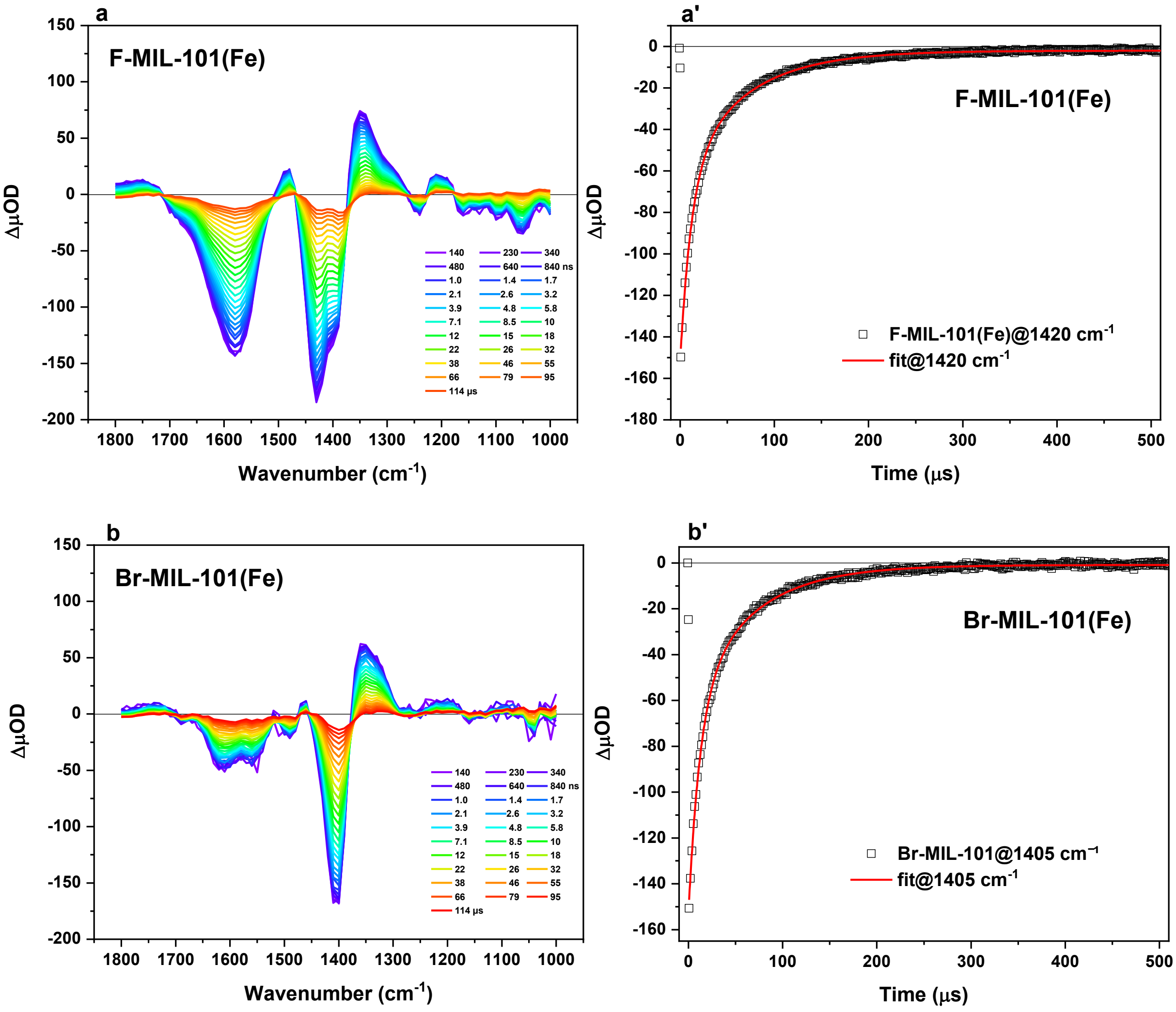

Figure S26. TRIR difference absorption spectra at varied time delays upon 532 nm excitation (left side) and kinetic traces of lifetimes at specific marked wavenumbers (right side): (a, a') F-MIL-101(Fe) at 1405 cm^{-1}; (b, b') Br-MIL-101(Fe) at 1400 cm^{-1} obtained by biexponential fit.

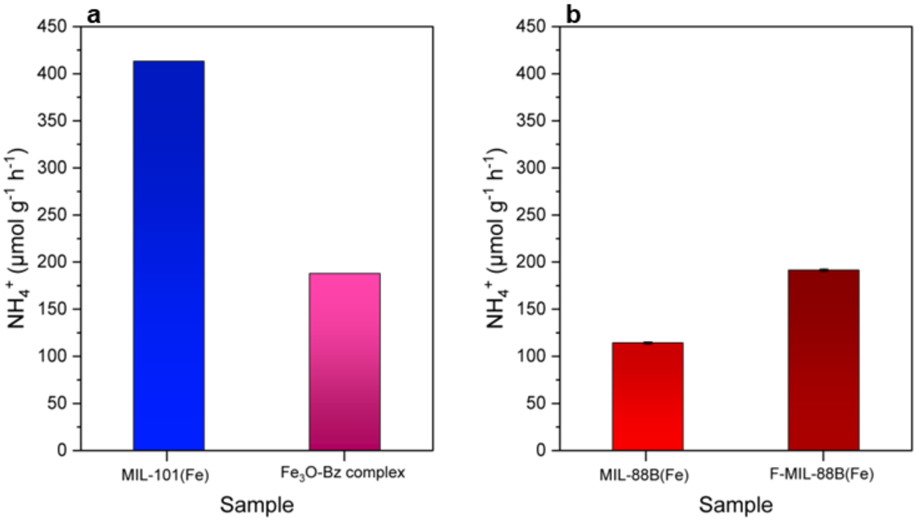

Figure S27. NH_4^+ production yields after 1 h of photocatalysis for (a) MIL-101(Fe) and the Fe_3O-benzoate complex and (b) MIL-88B(Fe) and F-MIL-88B(Fe). Values represent the average of triplicate measurements.

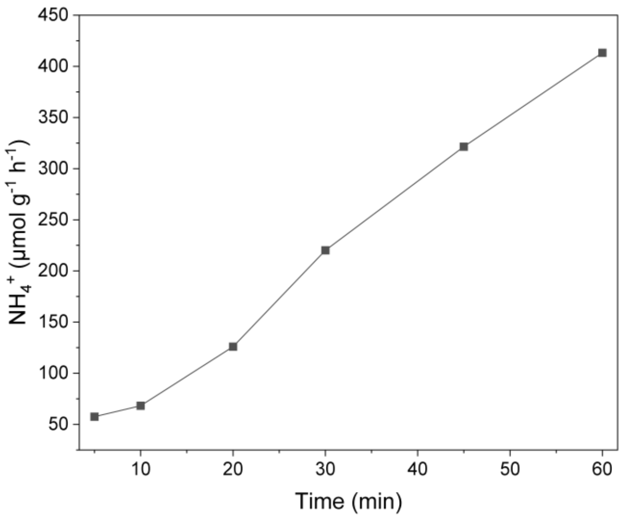

Figure S28. NH_4^+ production yields as a function of reaction time, determined using the Nessler's reagent method.

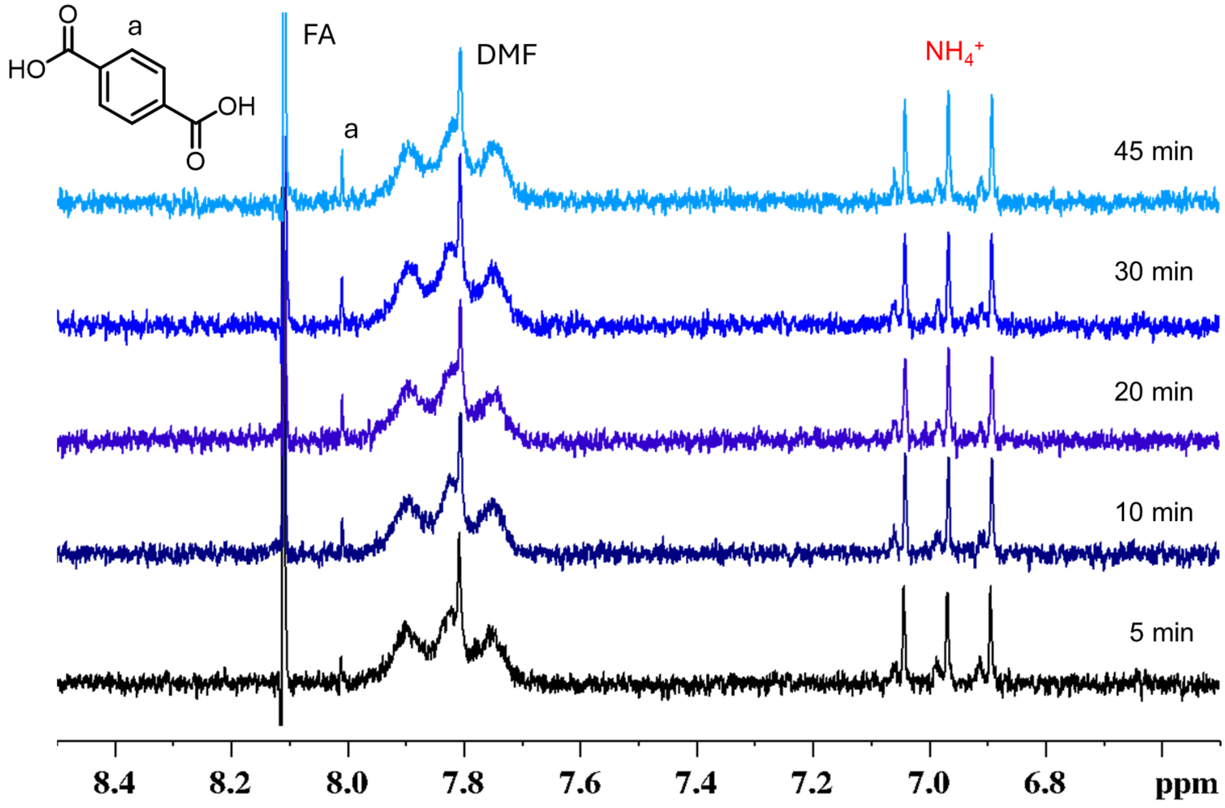

Figure S29. NH_4^+ production yields as a function of reaction time, determined by ^{1}H-NMR.

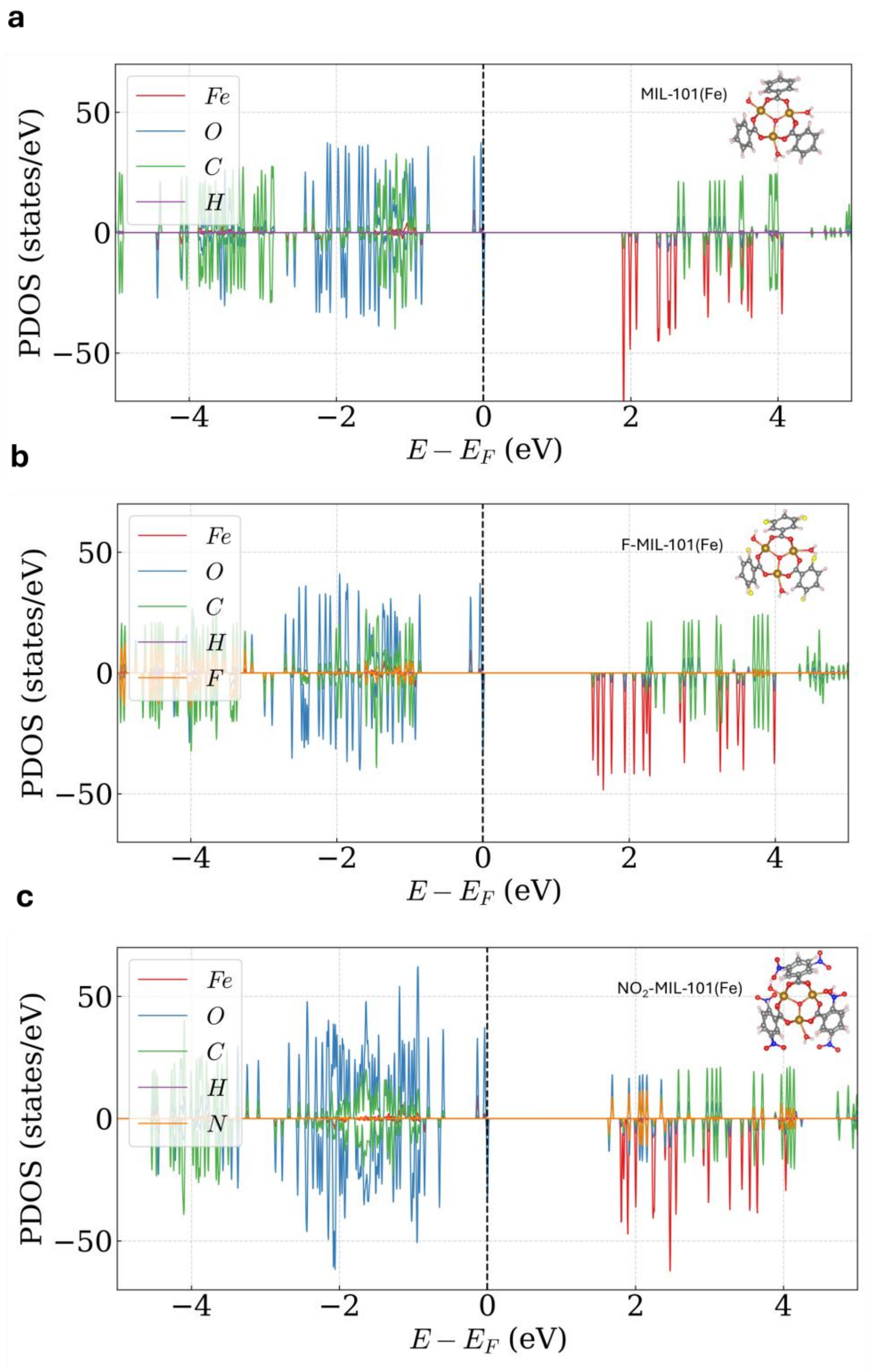
a
Fe
O
C
H
MIL-101(Fe)
PDOS (states/eV)
50
0
−50
−4
−2
0
2
4
$E - E_F$ (eV)
b
Fe
O
C
H
F
F-MIL-101(Fe)
PDOS (states/eV)
50
0
−50
−4
−2
0
2
4
$E - E_F$ (eV)
c
Fe
O
C
H
N
NO_2-MIL-101(Fe)
PDOS (states/eV)
50
0
−50
−4
−2
0
2
4
$E - E_F$ (eV)

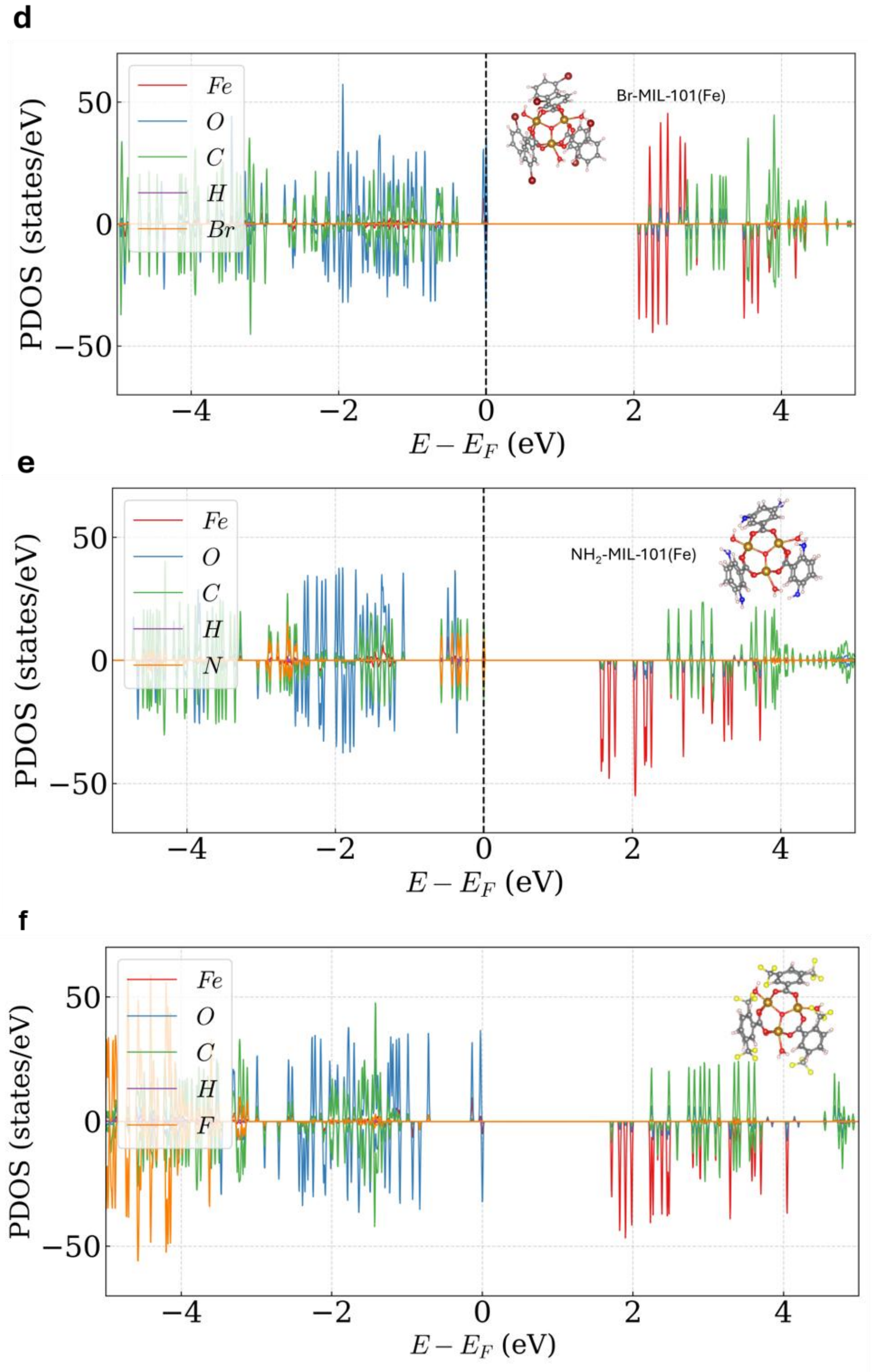

Figure S30. Projected density of states (**PDOS**) of single-ligand MOF samples with different functional groups. Each structure is modeled with a random distribution of functional groups, where three out of six ligands carry substituents at the ortho position to the SBU. Shown are (a) MIL-101(Fe), (b) F-MIL-101(Fe), (c) NO_2-MIL-101(Fe), (d) Br-MIL-101(Fe), (e) NH_2-MIL-101(Fe), and (f) CF_3-MIL-101(Fe).

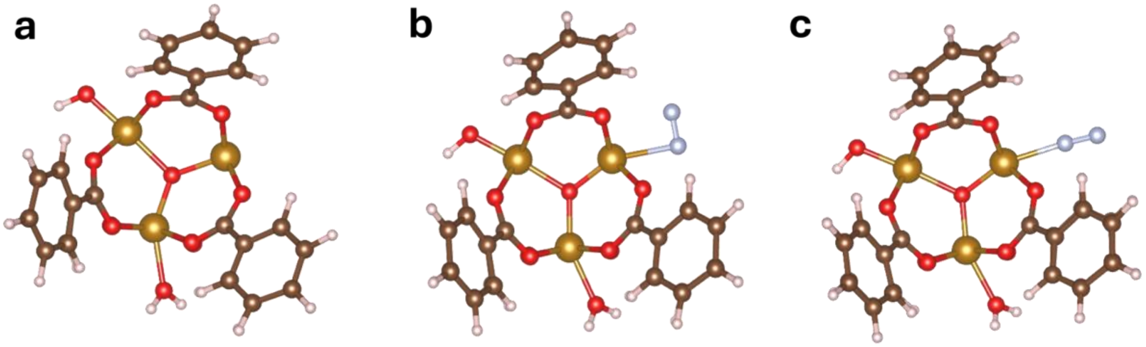

Figure S31. N_2 adsorption on MOF with iron exposed by removing terminal OH group (a) cluster with removed terminal OH group (b) N_2 adsorbed on catalyst side-on (-639.157 eV) (c) Nitrogen adsorbed on end-on (-639.97 eV). Because the nitrogen adsorption energy is lower in the end-on configuration (-639.97 eV) compared to the side-on configuration (-639.157 eV), the end-on adsorption is energetically more favorable.

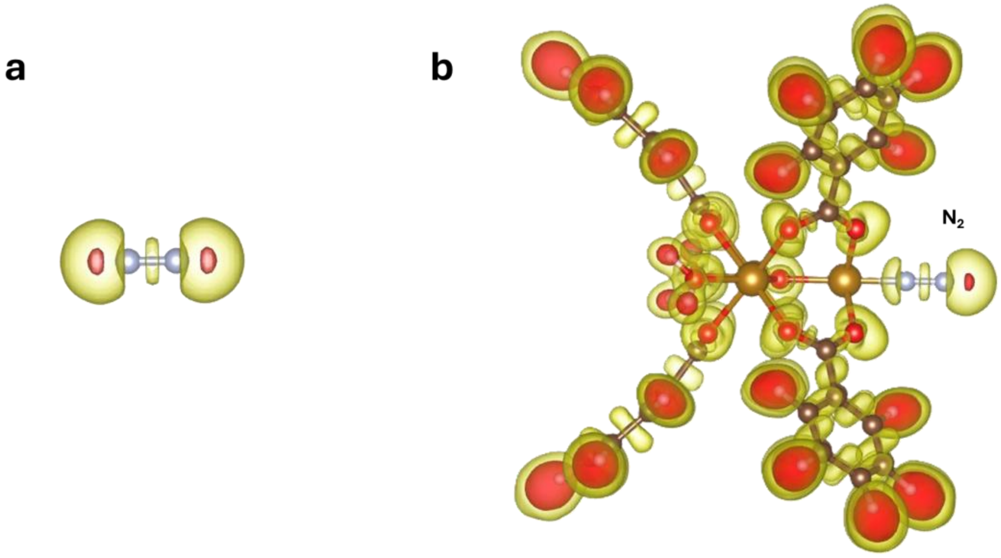

Figure S32. Electron localization function of (a) nitrogen and (b) Nitrogen adsorbed in end-on on the MIL-101(Fe) MOF, red color – highly localized electrons (ELF = 0.9), Yellow – ELF = 0.6, N-N triple bond can be visualized as well as the lone electron pair on nitrogen.

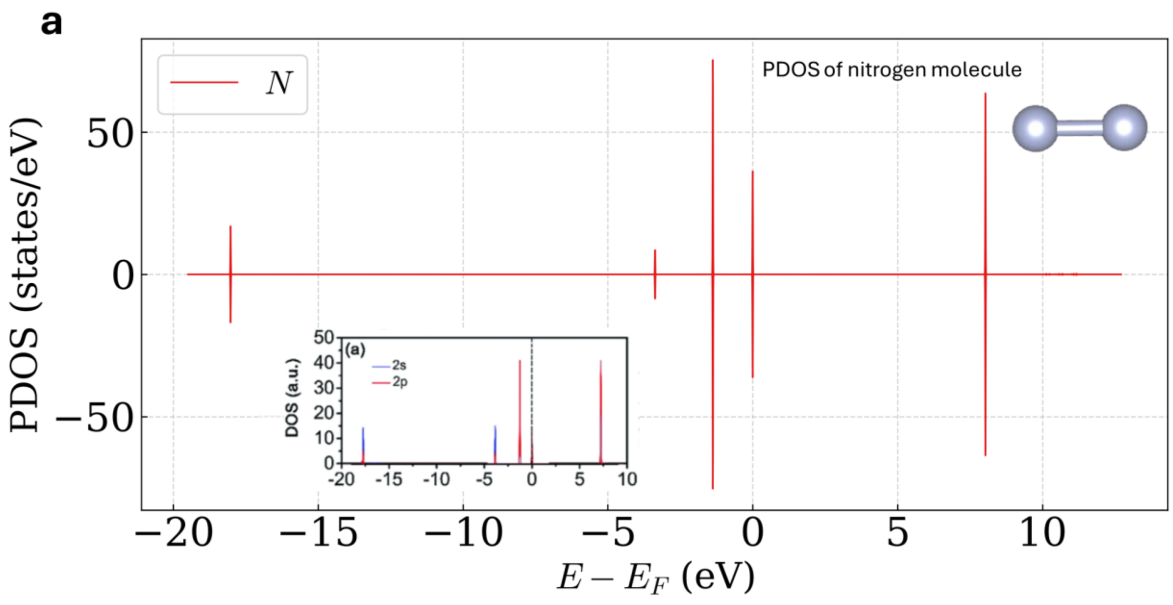

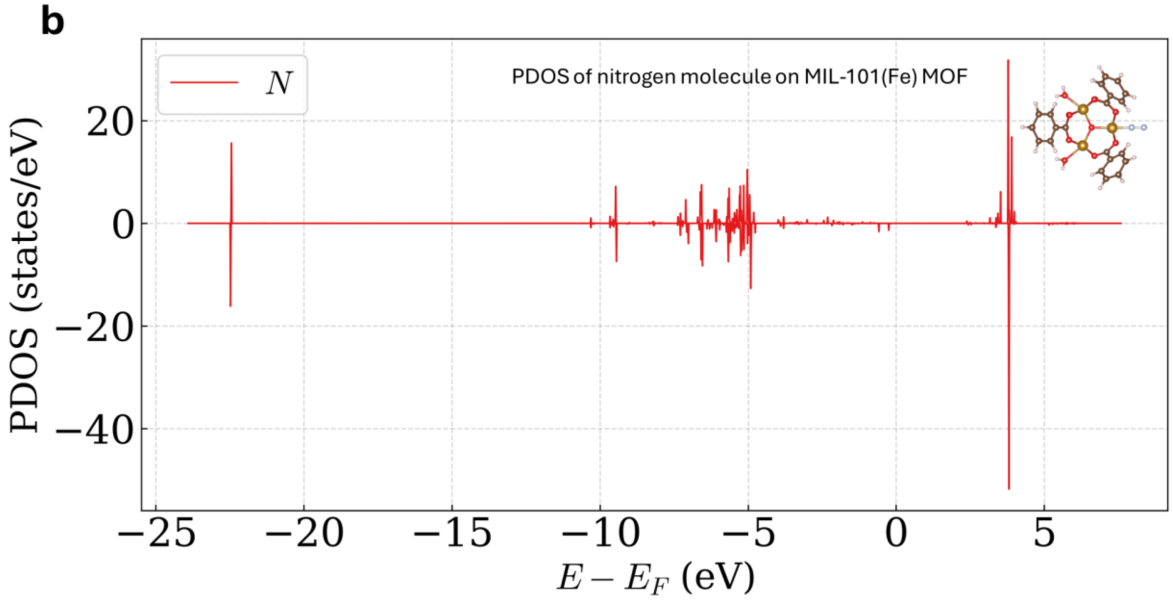

Figure S33. PDOS of MIL-101(Fe) cluster without terminal group: (a) before N_2 adsorption. (b) after N_2 adsorption.

S4. CATALYST STABILITY

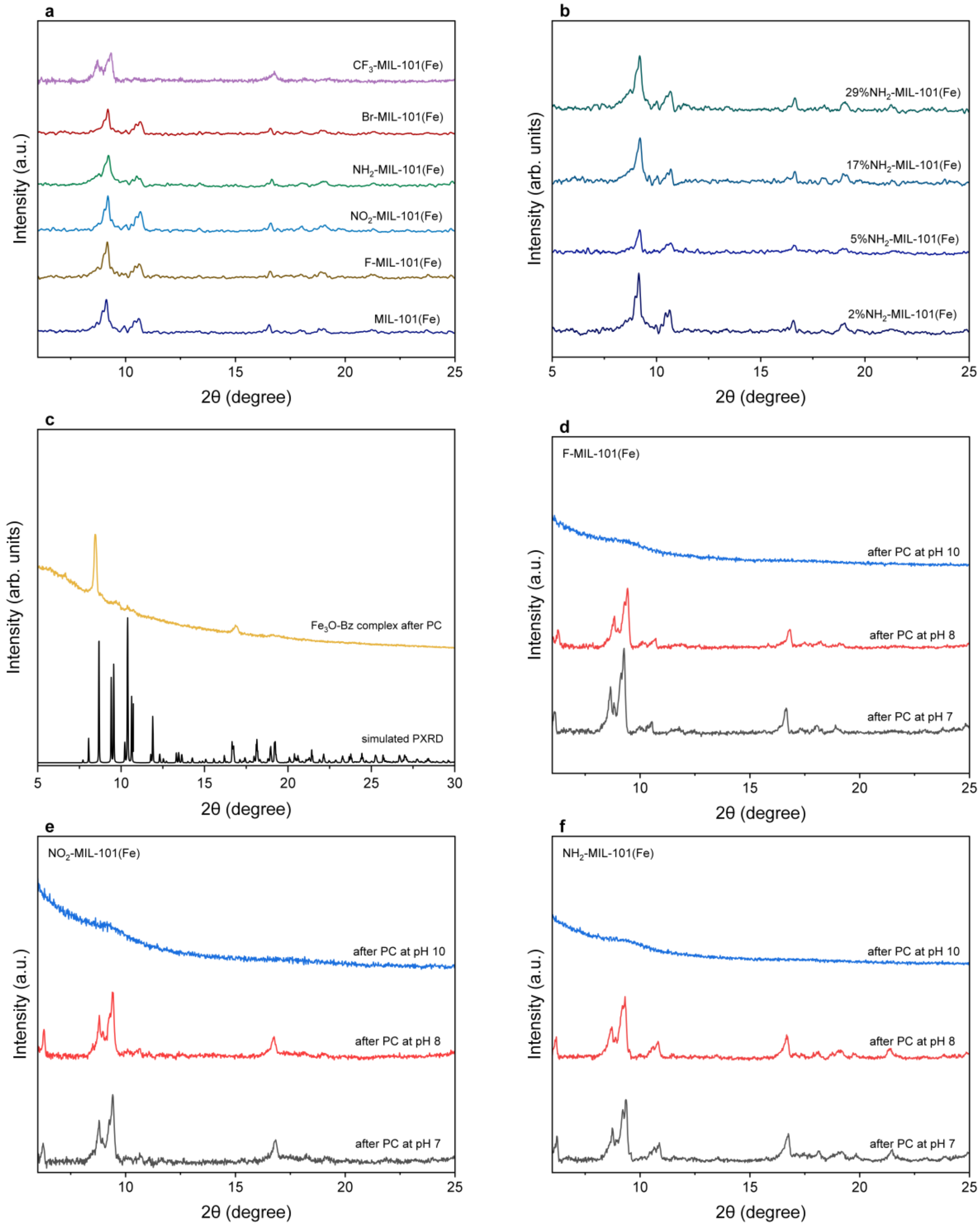

Figure S35. PXRD patterns after 1 h of photocatalysis: (a) single-ligand MOFs at their initial pH, (b) mixed-ligand MOFs, (c) Fe_3O–Bz complex, (d) F-MIL-101(Fe) at different initial pH values, (e) NO_2-MIL-101(Fe) at different initial pH values, and (f) NH_2-MIL-101(Fe) at different initial pH values.

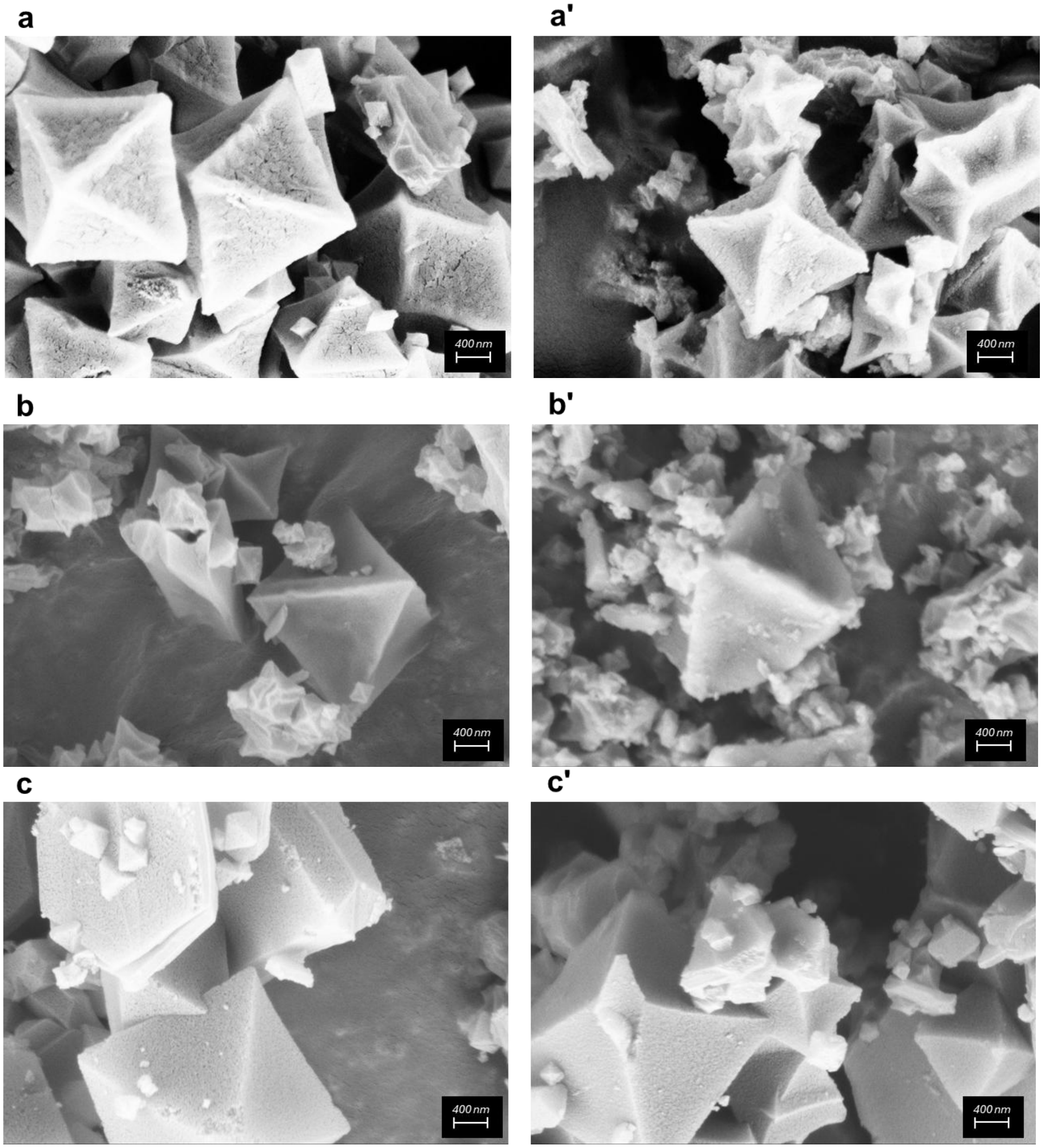

Figure S36. High-magnification SEM images of all MOF samples before (left) and after (right) photocatalysis: (a, a′) MIL-101(Fe), (b, b′) F-MIL-101(Fe), (c, c′) NO_2-MIL-101(Fe), (d, d′) Br-MIL-101(Fe), (e, e′) 2%NH_2-MIL-101(Fe), (f, f′) 5%NH_2-MIL-101(Fe), (g, g′) 17%NH_2-MIL-101(Fe), (h, h′) 29%NH_2-MIL-101(Fe), (i, i′) NH_2-MIL-101(Fe), (j, j′) CF3-MIL-101(Fe), (k, k′) MIL-88B(Fe) and (l, l′) F-MIL-88B(Fe).

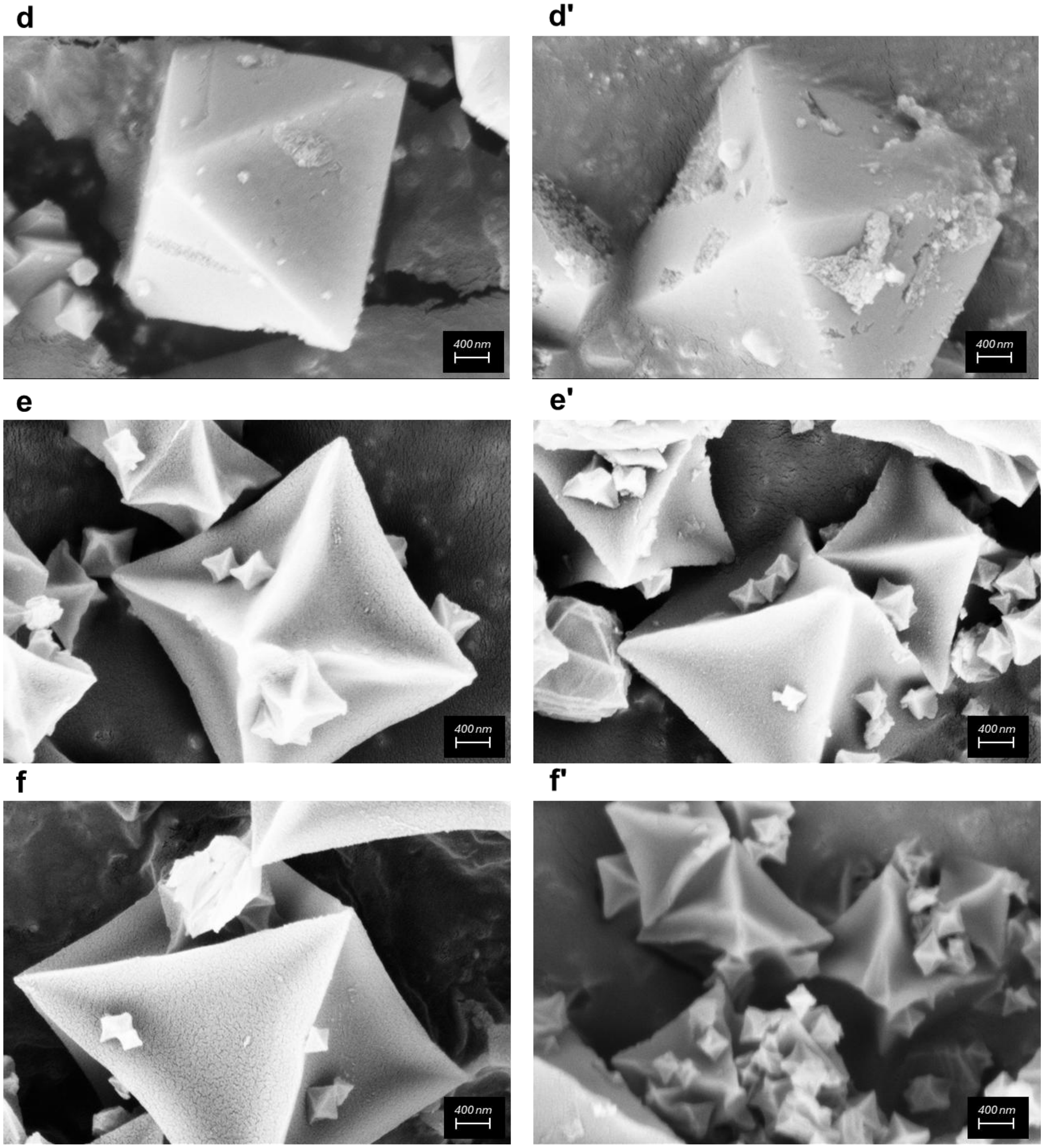

Figure S36. Panels (d, d′) Br-MIL-101(Fe), (e, e′) 2% NH_2-MIL-101(Fe), and (f, f′) 5% NH_2-MIL-101(Fe).

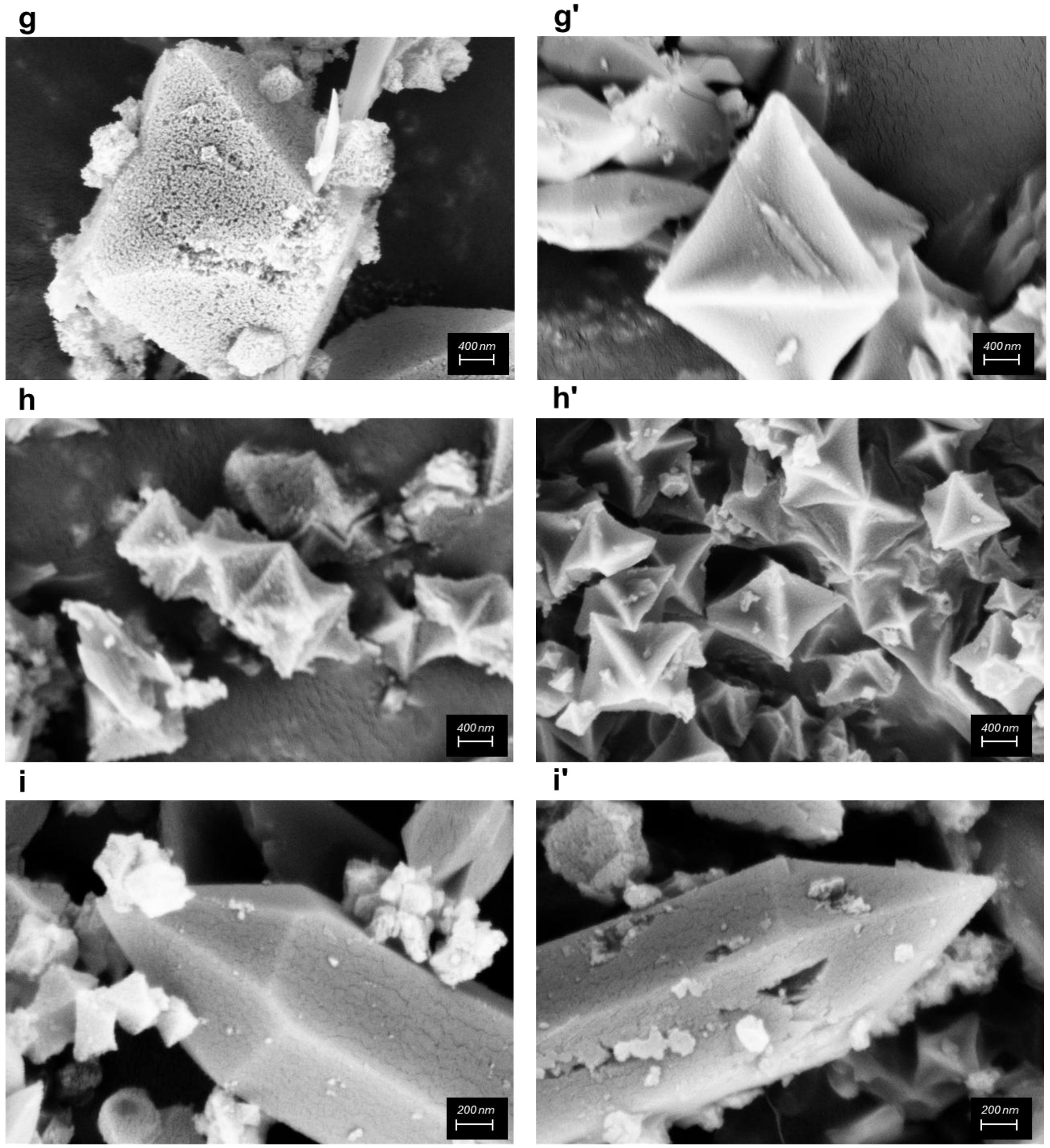

Figure S36. Panels (g, g′) 17%NH_2-MIL-101(Fe), (h, h′) 29%NH_2-MIL-101(Fe), (i, i′) NH_2-MIL-101(Fe).

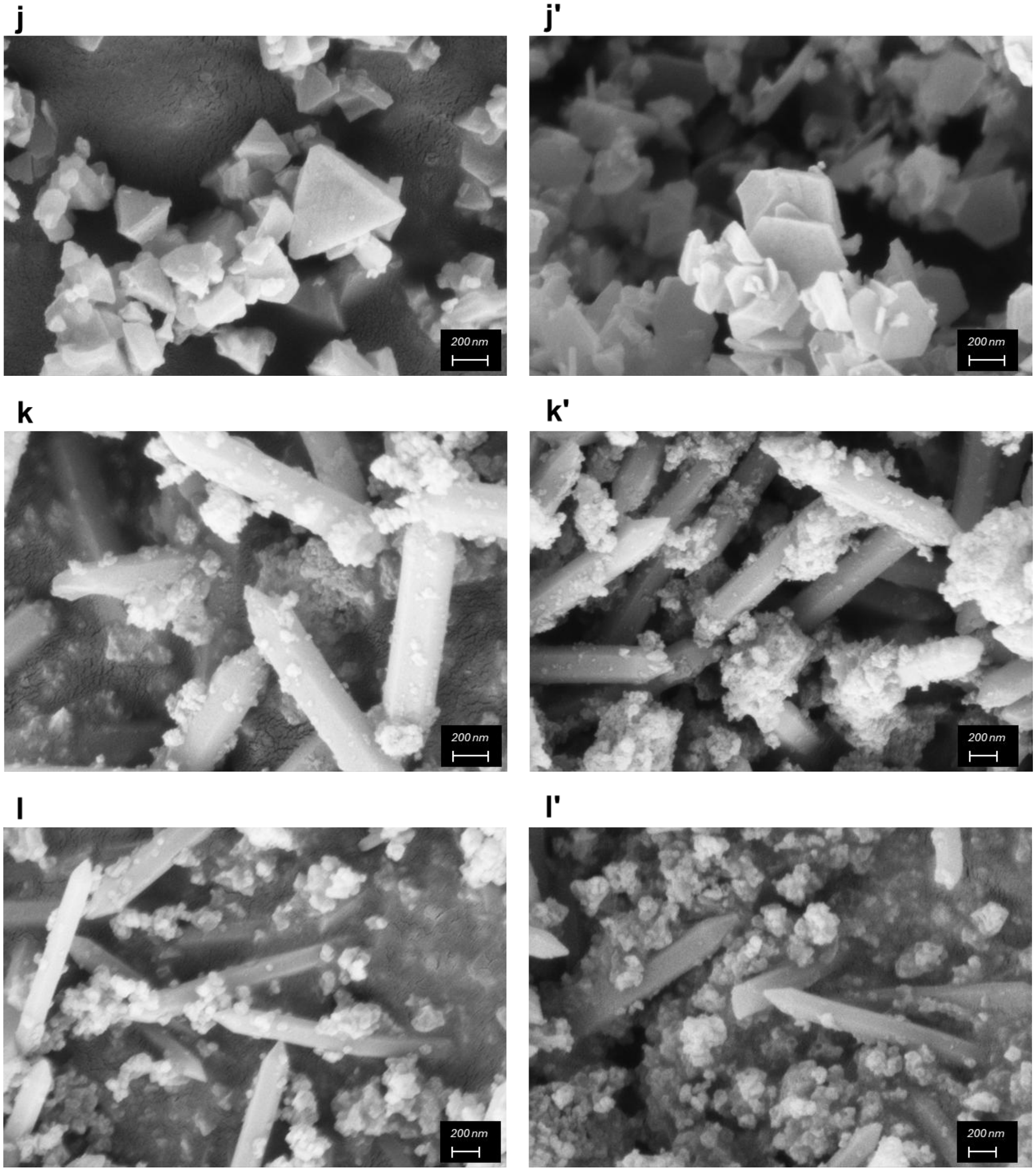

Figure S36. Panels (j, j′) CF_3-MIL-101(Fe), (k, k′) MIL-88B(Fe) and (l, l′) F-MIL-88B(Fe).

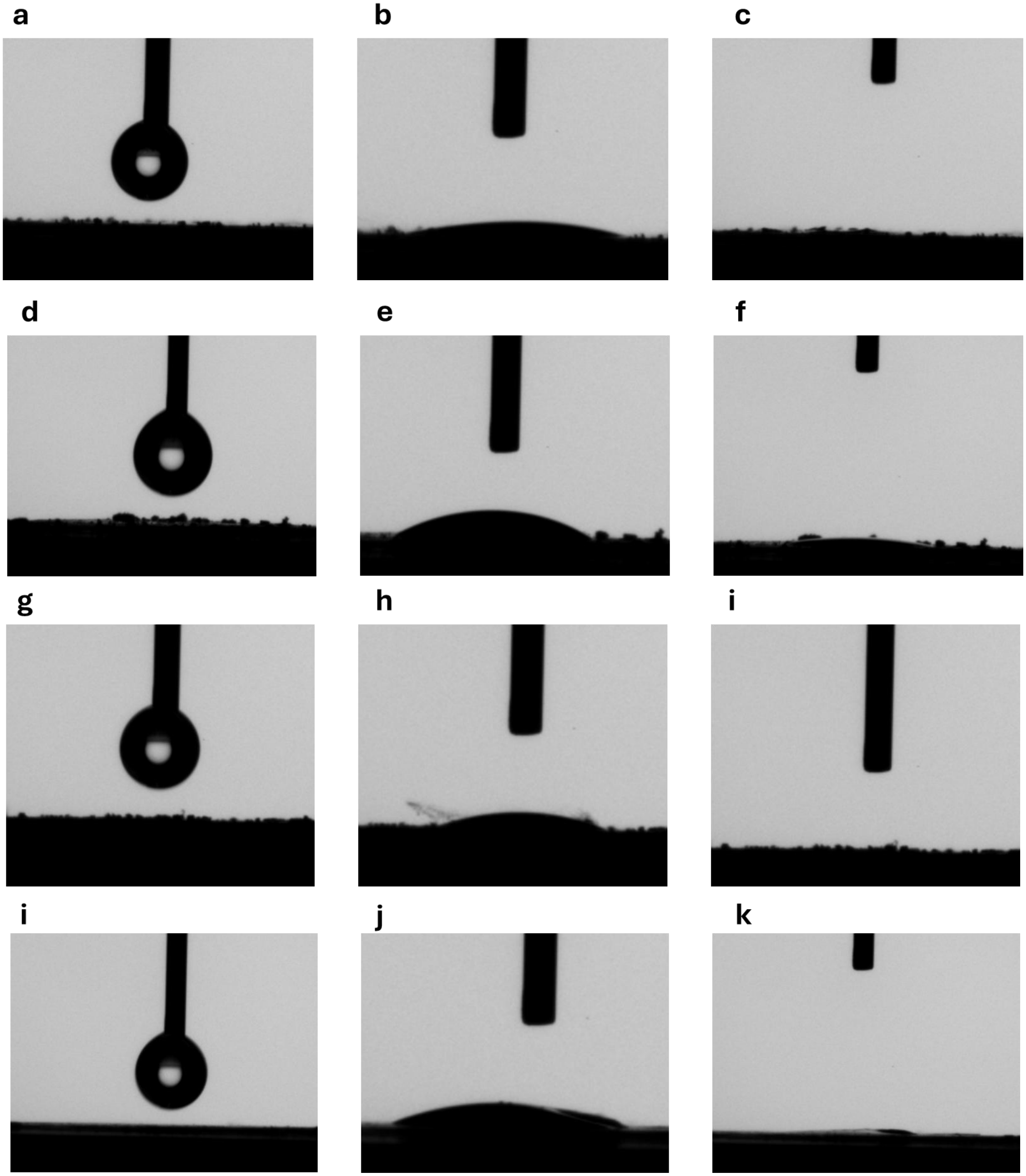
a
b
c
d
e
f
g
h
i
i
j
k

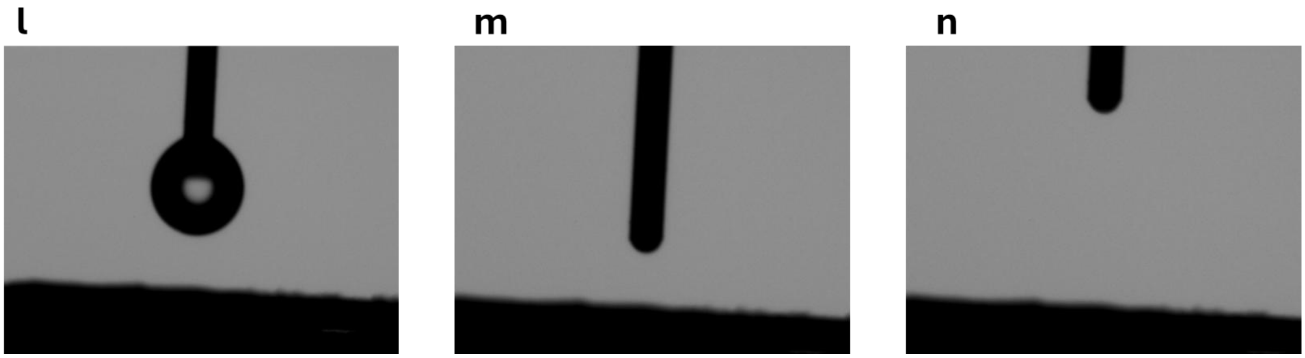

Figure S37. Water contact angle measurement: (left) before droplet deposition, (middle) immediately after droplet contact, (right) after 6 seconds: (a-c) MIL-101(Fe). (d-f) F-MIL-101(Fe). (g-i) NO_2-MIL-101(Fe). (i-k) Br-MIL-101(Fe). (l-n) NH_2-MIL-101(Fe).

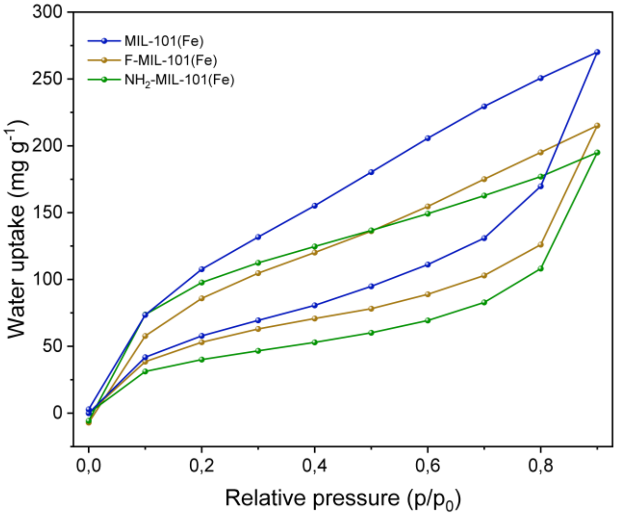

Figure S38. Water adsorption isotherms at 25 °C comparing unmodified MIL-101(Fe), NH2-MIL-101(Fe), and F-MIL-101(Fe).

S5. DFT STRUCTURE (WITH ENERGY)

Relaxed DFT structures with their energy are reported below in the VASP (POSCAR) format.

1. MIL-101(Fe) (-634.94147568 eV)

MIL-101(Fe)

1.00000000000000

27.9719696044999999 0.0000000000000000 0.0000000000000000

0.0000000000000000 27.9719696044999999 0.0000000000000000

0.0000000000000000 0.0000000000000000 27.9719696044999999

Fe O C H

3 16 42 35

Direct

0.5786921830953773 0.4989457605460075 0.4988849410185523

0.4752202680384983 0.4579527250278872 0.4582403616653536

0.4767186586540433 0.5413059010817562 0.5423439576171987

0.5048014443956603 0.4999508415827520 0.4999541735152278

0.5826492840750106 0.5738734083100852 0.4976073392930829

0.5825293759879742 0.4976596396911930 0.5738971677352254

0.5824871158621931 0.4260797705759174 0.5022433113970450

0.5824745267309552 0.5022350220948368 0.4260814176421448

0.5153596926613488 0.6005319114877068 0.5291446483742064

0.5131781302511129 0.5269533080937947 0.6026428230777796

0.5133940069627414 0.3973607789119598 0.4716823423985090

0.5133847469594102 0.4716757882841023 0.3973656878431839

0.4306910782137692 0.4309059477168944 0.5114238140195226

0.4307712564978203 0.5114796083834179 0.4308668519383900

0.4307370619063633 0.5691268509363283 0.4885484638568087

0.4331504181004391 0.4864029013990958 0.5715231237900511

0.4167412212035657	0.4239890867534015	0.4241053640007522
0.4173498895943339	0.5738948955214269	0.5738944386197744
0.6431141820420336	0.4998323384643300	0.4998665420660302
0.6142939196268884	0.7121028480724902	0.4440098558853549
0.6142721187349949	0.4439851301060145	0.7121378016535047
0.6142434289371721	0.2876069711264648	0.5558682907186991
0.6142934677845560	0.5558580726949245	0.2876871437043249
0.5893658521156979	0.7503816168701434	0.4639804174697204
0.5893779737619909	0.4641203105187230	0.7502833466854142
0.5915514140579319	0.2482090400610275	0.5338170499632611
0.5915605887485711	0.5337818277364619	0.2482387822501693
0.6025939543270269	0.6652242043441134	0.4555937822557752
0.6025774554745951	0.4556335052506810	0.6653064536706594
0.6025329126848433	0.3346217807250085	0.5443214492505248
0.6025078969254096	0.5443266647261851	0.3347030957799291
0.5537628329608637	0.6073340674165735	0.5061673095788564
0.5534417436871593	0.5046082202491959	0.6073278763633638
0.5534018493046133	0.3925741412505701	0.4938187503588622
0.5534046606984262	0.4938076920692822	0.3925805881727555
0.5648498955070664	0.6579562436196440	0.4887516496225643
0.5647363819663269	0.4886472050014561	0.6580216951724438
0.5646936804384950	0.3420153389111178	0.5112214371125674
0.5646775060423792	0.5112038376222898	0.3420108740216889
0.5491700869694043	0.7432526870166285	0.4918078253444307
0.5490760940714452	0.4919577576273966	0.7431375906346886
0.5511284393365230	0.2566938277303947	0.5062861077966119
0.5511256382030325	0.5062838893126553	0.2567040366111897
0.5375823297718014	0.6965144829500716	0.5046271519273944
0.5358854702515856	0.5044544003342324	0.6963954468301523
0.5359842721379380	0.3035169666996111	0.4955213095589883

0.5359803921881507	0.4955122045881382	0.3035264765563710
0.3108413395163723	0.3682233193584921	0.6318085466171297
0.3108341676214934	0.6317767378536061	0.3682324656134170
0.4197453948425363	0.4464475440235489	0.5535926119442180
0.4197085845220911	0.5534359993642823	0.4465381299686229
0.3881929143255647	0.4150122851847513	0.5847674716620759
0.3882569672824445	0.5847946895629903	0.4150903144963252
0.3327354614077578	0.3464842326398099	0.5914389316340021
0.3327219101000054	0.5914296526786273	0.3464961540058269
0.3327800774810115	0.6534875631771229	0.4083720969607612
0.3326931132967630	0.4084386068522647	0.6535141656767820
0.3704780633183375	0.3706150673747430	0.5671053899508181
0.3704773843527818	0.5671089002785337	0.3706417206476900
0.3705982318229886	0.6294128192164692	0.4313084477130573
0.3704622718704798	0.4313118839891743	0.6292521444161210
0.6562873170514365	0.5246629630954516	0.5201872177083828
0.4106638560291458	0.4218111987191193	0.3904552028444073
0.3912926987054703	0.4107174318473596	0.4435834730672426
0.4128530823178664	0.5759400514269686	0.6085413343929318
0.3914773558134073	0.5889908458033801	0.5558488024562536
0.6017459096643520	0.4570559742693447	0.7871618285038551
0.6428782833677431	0.4182030563773296	0.7203427250884147
0.5289991380765997	0.5044953595422754	0.7741230088624178
0.5066704143872585	0.5287098453013783	0.6878424175421856
0.6241531578286015	0.4435307299979527	0.6354848353673432
0.6233744910428882	0.6347312856178036	0.4435370309028102
0.5087527543471353	0.6896456709461702	0.5291355526119688
0.5289839717413116	0.7740940530107991	0.5045053911013682
0.6004979485453319	0.7871824006334052	0.4553627264985067
0.6428774594847297	0.7203205606230796	0.4182019009973459

0.6228762274493533 0.5561195114991477 0.3657669190831072
0.6428673678596510 0.5818344546667049 0.2797107620286923
0.6045263580628699 0.5402085152814564 0.2119878022190207
0.5314100526700258 0.4931504178840953 0.2257958156949513
0.5061738565235103 0.4713190195234560 0.3107423572936838
0.3212448262142260 0.5781333280386391 0.3111850628308375
0.6241401359132723 0.3644898822278861 0.5564914865144033
0.6428571425514278 0.2796928721873755 0.5818423185671264
0.6045370445551370 0.2119786986525867 0.5402362236984501
0.5314151598907486 0.2257872925558075 0.4931562596336150
0.5061704439629224 0.3107332853834279 0.4713432836191416
0.3861704728927293 0.3570830373477563 0.5339532274945427
0.3212430594755915 0.3111755935338465 0.5781477907967627
0.2798066449393701 0.3523504605717989 0.6495004293616802
0.3212748624835697 0.4218841096849744 0.6888171051453895
0.3840247280554863 0.4658498438870282 0.6413034349901210
0.3235168499642853 0.6894626835934048 0.4199842151026303
0.2798183044879750 0.6495077560090152 0.3523804197266784
0.3862057158397647 0.6448896618748989 0.4638760006459606
0.3861671860966283 0.5339423773360394 0.3571356394818466

2. NH_2-MIL-101(Fe) (-707.56111113 eV)

NH2-MIL-101 (Fe)
1.00000000000000
27.9719696044999999 0.0000000000000000 0.0000000000000000
0.0000000000000000 27.9719696044999999 0.0000000000000000
0.0000000000000000 0.0000000000000000 27.9719696044999999

Fe O C H N

3 16 42 41 6

Direct

0.5783766454838073	0.4987001092705015	0.4986316510881394
0.4756023372666931	0.4577172318917135	0.4584028128091191
0.4767392049416017	0.5409261245446828	0.5425228382590319
0.5060421491240277	0.4999741660125636	0.4999807757692452
0.5826737959665707	0.5737714752214558	0.4975197203911037
0.5824480524489957	0.4976518680594140	0.5738593689428981
0.5825246514503419	0.4260964467870920	0.5022580437188253
0.5824575731775852	0.5022348296456585	0.4260864409963361
0.5153640185955268	0.6005670006788932	0.5290154393094610
0.5134362000943824	0.5282816449591508	0.6026683074221637
0.5131637647633909	0.3974208834282535	0.4730612477899285
0.5133886181468910	0.4716692445442163	0.3973544569734528
0.4306843071424566	0.4309258041072610	0.5114078949152940
0.4331336624605413	0.5132287703500751	0.4287039084162885
0.4308040486497831	0.5689545510259109	0.4886269025182486
0.4331594894823638	0.4864014630875388	0.5715001400412731
0.4167267217489865	0.4239446884720621	0.4240261304399908
0.4173464000820530	0.5738945073606629	0.5738904584987736
0.6431266746464743	0.4998594600903559	0.4999306478511443
0.6126049352934544	0.7130887537741444	0.4410524083750929
0.6142211809250924	0.4438967467623911	0.7120981402419204
0.6117854970582357	0.2858447841126548	0.5579097173789123
0.6143002205637629	0.5557768488498951	0.2877351398842478
0.5914888781511536	0.7519232001229597	0.4660491112214942
0.5895804904936668	0.4638577781268722	0.7502988876805290
0.5909396947066412	0.2479217224205641	0.5336214326322164
0.5917280446639026	0.5338625139890496	0.2481779341182104

0.6024616411074817	0.6655571744965414	0.4556995979588692
0.6026162881391102	0.4555160678115442	0.6652056838346638
0.6027627645201505	0.3345496046093785	0.5443144577270900
0.6025573737245935	0.5444789988890406	0.3347580471992373
0.5535738135302140	0.6072189424435095	0.5046297238000790
0.5533930144717090	0.5047221693790220	0.6073491963121427
0.5532989943367141	0.3923762480521304	0.4952906610163268
0.5533987038144588	0.4937608708964234	0.3925573321201554
0.5647521203287837	0.6580351687087145	0.4886708928590053
0.5648816565429371	0.4886282818919909	0.6579867988402057
0.5646810807933065	0.3420505343854714	0.5112776317367604
0.5650340423731066	0.5111507732177074	0.3420213783953443
0.5512003049351435	0.7432408811601690	0.4937661260998425
0.5492573110411669	0.4913159345654563	0.7428516795410758
0.5494268124843913	0.2563658444273216	0.5047386813384591
0.5514195449343120	0.5062620171836727	0.2567103626322620
0.5359065977134634	0.6964248448785924	0.5044771858528279
0.5376019014556519	0.5046091642405486	0.6963039706484935
0.5358758832479609	0.3035105496290029	0.4954967271315454
0.5400243400718097	0.4929273453414140	0.3035043416730261
0.3108624182513537	0.3680539912588046	0.6316417754371386
0.3128315823844261	0.6321783725436347	0.3661671555747290
0.4197198714564294	0.4464342190425299	0.5535682258058188
0.4197019072537600	0.5534865127866979	0.4462812213730274
0.3882257355378798	0.4151116095928558	0.5846494144447902
0.3883147860673475	0.5847735007736716	0.4152319453010591
0.3347959811152421	0.3459887534833825	0.5928536202337256
0.3330099113128853	0.5912698415800790	0.3460112443904464
0.3347352461339241	0.6518778600356612	0.4062821195752662
0.3347290342075979	0.4064696912014440	0.6518776855044806

0.3705693249177173	0.3706997907254248	0.5670908337852296
0.3705119122676663	0.5685778171264602	0.3706461948330642
0.3706428070602712	0.6293257680984539	0.4329769709943960
0.3711286067095116	0.4321896625680779	0.6286461644656001
0.6523132862254570	0.5277718921105858	0.5188664018656013
0.4083595541096940	0.4241219868878403	0.3904919768114183
0.3912866566545716	0.4107460621908530	0.4436197980521754
0.4128156988368019	0.5759125969131276	0.6085355832959465
0.3914235939225250	0.5890056710212122	0.5558582351827752
0.6017538953044195	0.4570008569211552	0.7872696650911664
0.6428565842321348	0.4181660571875057	0.7203286781973119
0.5086688515337414	0.5290843963018261	0.6896838154422014
0.6234806491305633	0.4431980881936610	0.6349869231081158
0.6240797660042858	0.6355391918942672	0.4434565273414535
0.5066358261579893	0.6878541374222138	0.5286928231168702
0.5314378327463203	0.7742101408198891	0.5068663449255837
0.6044968593138336	0.7880455103784172	0.4597238893604541
0.6233324109379836	0.5565150925298497	0.3652464851680293
0.6435807278469028	0.5809634333132649	0.2811700862526010
0.6048669068668104	0.5385282961443352	0.2116005126885838
0.5109595898803647	0.4686794306703561	0.3103410525692851
0.3213818144539076	0.5780098944976473	0.3110450026187621
0.6411441430528413	0.2789761989149468	0.5830108423965115
0.6043875253949054	0.2119467733505402	0.5401716555890417
0.5277756949527799	0.2260440208325605	0.4925981195630840
0.5065917023784721	0.3123410314605408	0.4714196075299881
0.3858888174337949	0.3565155566410709	0.5339645653048422
0.2795656336218002	0.3504592729571385	0.6473560153193603
0.3229087142822067	0.4182869610073823	0.6866740151625237
0.3839612739264311	0.4665918003324876	0.6411885395956034

0.3227344470528664 0.6864037543486603 0.4184788276630869
0.2816841872563387 0.6496228213770934 0.3503627588475027
0.3857348348177325 0.5348345691782868 0.3571445051729327
0.6496448713174985 0.6901009502543545 0.3841455235592548
0.6278782036707256 0.7447044245822383 0.3778324710758483
0.5360982807212125 0.5055893031022336 0.1864740671414467
0.5088998634962394 0.4619120397417120 0.2166979083043188
0.4174562692987749 0.6281538409749032 0.4867606323576510
0.3925887978892462 0.6844429614515590 0.4755095998901382
0.5354013924470280 0.4934250117541836 0.8139015977938939
0.5089919236007461 0.5380672571050624 0.7830540114422959
0.6635017536392311 0.3556627032988402 0.5741333366817898
0.6399421760742428 0.3973725778475270 0.5342033936838035
0.3452178139738464 0.2856932191145560 0.5557323506536065
0.3232242296799299 0.2770399593812698 0.6094485526518199
0.5222515505721574 0.5044733313338980 0.7814070269262601
0.6428993472171101 0.7214411830344929 0.4017469969115979
0.5222702751972506 0.4954926126171415 0.2186552078094977
0.6339578407435553 0.3704425548897206 0.5581017541021822
0.3214607412659092 0.2990836834335013 0.5803180052940462
0.3884105760960068 0.6480134005632365 0.4753552793682516

3. Br-MIL-101(Fe) (-621.98520059 eV)

Br-MIL-101(Fe)

1.00000000000000

27.9719696044999999 0.0000000000000000 0.0000000000000000

0.0000000000000000 27.9719696044999999 0.0000000000000000

0.0000000000000000 0.0000000000000000 27.9719696044999999

Fe O C H Br

3 16 42 29 6

Direct

0.5811871454366653	0.4987584812271919	0.4986800190600036
0.4773989325323100	0.4584939606414622	0.4585422761960913
0.4766921397630526	0.5413519573795043	0.5401779611381556
0.5088183814198288	0.4998442466267079	0.4998306578466511
0.5826952288749112	0.5737101865448864	0.4975126682025035
0.5806185867533102	0.4953490831681364	0.5715898344060264
0.5804217241345668	0.4270552472389184	0.5044936241459865
0.5825196644970987	0.5023133820366468	0.4261013533442863
0.5153728446860839	0.6005801874693901	0.5290058561489914
0.5153653733474641	0.5290555012798919	0.6005867775534881
0.5153569530868083	0.3952612749361890	0.4708900293818061
0.5131662277422322	0.4730338909517471	0.3973852223839245
0.4306653708406714	0.4307974507173000	0.5114108997976317
0.4351925728678978	0.5158487926542605	0.4262730262929750
0.4329451856088227	0.5671910244445542	0.4867872779061742
0.4330331250622947	0.4865852972859699	0.5685251995848475
0.4151789725368147	0.4284950997641275	0.4269888448798014
0.4158351386799950	0.5720762794923786	0.5721660366468129
0.6445001231952219	0.4999708939876868	0.4999525559036400
0.6132488372386717	0.7138583182950384	0.4463811287281416
0.6137976828168163	0.4437735982038760	0.7120183630339056
0.6116617564378046	0.2856862917737644	0.5581180937735368
0.6134322536624310	0.5558832185803055	0.2879497527879806
0.5870459107490689	0.7517546401778432	0.4663924713057312
0.5891698420475109	0.4641309888690728	0.7501633903514886
0.5866116587658823	0.2479708328908643	0.5378729501079889
0.5891568661214635	0.5355916546452448	0.2497991542172358

0.6026285074744067	0.6653064364336814	0.4553896710959577
0.6026018035752969	0.4553618234236012	0.6651356670005200
0.6027273326163254	0.3329232683793251	0.5444387784127187
0.6025809754025815	0.5428574616995192	0.3347090905950623
0.5535109185413702	0.6070470392998715	0.5045214157098385
0.5534832546128925	0.5044905656096219	0.6067355951166320
0.5535731996489091	0.3927143250284644	0.4954377897151687
0.5533734901304896	0.4953027563104015	0.3928305064152013
0.5643285494375760	0.6563144956519622	0.4868125350096975
0.5647625446869569	0.4872965377182013	0.6562100729152505
0.5665095009716268	0.3436213632362382	0.5112472094889924
0.5654249929890014	0.5106155745044489	0.3434124869179200
0.5474211836089466	0.7411806486236046	0.4953753091655386
0.5490455483269443	0.4914159365486128	0.7408230858985121
0.5490634769583309	0.2587686771423208	0.5062648965092436
0.5509140234268131	0.5045507958413964	0.2590105239065466
0.5374744501380917	0.6940185081010171	0.5066493458998949
0.5380201121452188	0.5062117403258029	0.6942558473771854
0.5383360535364687	0.3056058447541048	0.4933640066662761
0.5380980787413918	0.4929134489311053	0.3055687903464914
0.3125805793783343	0.3661393055434914	0.6339011886422981
0.3126525497518813	0.6338304901626231	0.3660127262315100
0.4197172887608929	0.4463452818770932	0.5533972586270011
0.4219176625254377	0.5552438449285049	0.4442551646748853
0.3882492867666230	0.4150889934495439	0.5847633473740927
0.3883383704932086	0.5849261154297025	0.4150713583555117
0.3345777703783241	0.3479200722759686	0.5932753835017976
0.3346510610898221	0.5933475278622353	0.3458961996743639
0.3338813093101578	0.6539522300544505	0.4061704854341457
0.3347066657976328	0.4064219425414706	0.6537115774284672

0.3705925366775418	0.3707348453798502	0.5671848673859472
0.3705875821058342	0.5687407044269932	0.3704753765156994
0.3705635464708408	0.6295562193182676	0.4288807552530329
0.3706970230358095	0.4311333108969748	0.6292824014237581
0.6543569618899525	0.5264634326407673	0.5201512472817953
0.4126263385948903	0.4217707584455468	0.3931158129836589
0.3910574448561874	0.4107636220964537	0.4443091862563193
0.4126131609159529	0.5737081445847778	0.6067161365749740
0.3929868849966098	0.5917688146035474	0.5555903351283789
0.5981940242873733	0.4574338350627372	0.7875217222835360
0.6408637040083960	0.4172642121336381	0.7187728251358720
0.5109977295022148	0.5330250508064296	0.6874437340616169
0.6224038964488017	0.4392730844062385	0.6355983657024282
0.6231852088692094	0.6356876384028212	0.4397124141723481
0.5073586352101174	0.6847499401892634	0.5302460860139391
0.5249490558681842	0.7701244703888079	0.5088304672100463
0.5978229996848370	0.7880192606888059	0.4576926592553150
0.6228561784047670	0.5579480604222411	0.3640278906546754
0.6407647114651809	0.5826581433558289	0.2812083609196563
0.5998193988660816	0.5411508476450706	0.2126132533802547
0.5109240419802532	0.4664074737543444	0.3124831062658657
0.3235431662217465	0.5803541833020205	0.3107063811831452
0.6381424291262334	0.2789630400893017	0.5862175325323733
0.5957297513025566	0.2112907606370911	0.5469898950033638
0.5268763610114959	0.2297570043400867	0.4930313155162906
0.5086473109452641	0.3128551791641883	0.4685438205916768
0.3860164819068288	0.3548928933762454	0.5354291945783061
0.2797737401379266	0.3503386553917025	0.6476509117335070
0.3234845513177618	0.4196173074052751	0.6889263071842109
0.3857527677583121	0.4636434846366626	0.6446906411775259

0.3225379173058798 0.6884858843997392 0.4200210135217120
0.2813615010336079 0.6497427161329057 0.3501189860850928
0.3859339461202183 0.5360680144230159 0.3550283534518783
0.5106310940711651 0.5147661809516393 0.7922626138723246
0.6637051773065465 0.7280695017634073 0.4026825559275338
0.6467252538707129 0.3780113678919577 0.5691742150056669
0.3085878133956399 0.2897611759838910 0.5667528101572614
0.3916052350566872 0.6632690329101081 0.4851533975762976
0.5155553808447166 0.4776448625800285 0.2070293318101832

4. F-MIL-101(Fe) (-635.81509859 eV)

F-MIL-101(Fe)
1.00000000000000
27.9719696044999999 0.0000000000000000 0.0000000000000000
0.0000000000000000 27.9719696044999999 0.0000000000000000
0.0000000000000000 0.0000000000000000 27.9719696044999999
Fe O C H F
3 16 42 29 6
Direct
0.5783238827449395 0.4984261332191977 0.4987157765799282
0.4737168791313309 0.4573647135687011 0.4582601363154453
0.4758068074494446 0.5409334700599118 0.5413859012555022
0.5060781616091390 0.4999868032189525 0.4999906578710451
0.5827269186941280 0.5738122259481173 0.4976033620498441
0.5825367053868078 0.4976410374918530 0.5738901691953657
0.5824143116873941 0.4261612703175075 0.5022203174211413
0.5823580383493407 0.5022039445900077 0.4262742838103080
0.5153610986296968 0.6005337933893955 0.5291301822016408

0.5131606290042612	0.5269256183118571	0.6026243547001542
0.5134367421470429	0.3972938073426491	0.4717037254614240
0.5133940270516675	0.4716773915492851	0.3973455516928581
0.4306977596623369	0.4308387834771565	0.5114612487740686
0.4307354652895157	0.5115008003165258	0.4308477890716205
0.4307853670080632	0.5690413819858762	0.4885650348132344
0.4307707065884827	0.4885003411174438	0.5691462002931473
0.4167340116939329	0.4239681993203561	0.4240886765829757
0.4173451424460453	0.5739124082893667	0.5738887041115461
0.6431107753743817	0.4998699884619313	0.4999041977843248
0.6116074434114083	0.7124946850731462	0.4422254588482630
0.6141789382634144	0.4442212203572993	0.7124951555007186
0.6142988355192216	0.2876241077240849	0.5558963831104506
0.6142479925156437	0.5556824083902043	0.2876469203828194
0.5915174881292558	0.7520370864366441	0.4659815538751459
0.5913665619349260	0.4662396251522978	0.7517376704933554
0.5915870870444309	0.2481433066310856	0.5339670284468809
0.5912426848620100	0.5338500036368146	0.2481603847362592
0.6024607437832401	0.6654036017236322	0.4555771312658692
0.6024224935480476	0.4556962618574261	0.6654785845291826
0.6027076277948922	0.3346629001804899	0.5444470985379013
0.6025550349196536	0.5442629533918577	0.3347013907882825
0.5536810714628544	0.6072635541620244	0.5062035009624211
0.5533800044171286	0.5046472206002974	0.6073085347542317
0.5528810294181454	0.3927510384994335	0.4941912838781874
0.5534984108048775	0.4929216393274558	0.3928037822388788
0.5648759916084387	0.6579793322601759	0.4888085573847434
0.5647750609028606	0.4887056685038118	0.6579934943322456
0.5643655865942279	0.3436803320918997	0.5131613845424212
0.5669238369083018	0.5090420524308783	0.3436556672390410

0.5513717134265192	0.7433500535601496	0.4937896973764140
0.5492621106517888	0.4910667255770562	0.7413391402168088
0.5513590053906370	0.2566536501456369	0.5062434292884674
0.5492531542936732	0.5089449705316795	0.2591571272741362
0.5375760360092698	0.6965493613305469	0.5046533719135127
0.5358196037161562	0.5045015736864187	0.6962203434508538
0.5384646255091781	0.3036430525391225	0.4956475794403161
0.5383443796309635	0.4932648181735431	0.3055273487531025
0.3108163631661682	0.3681948415472718	0.6318474873908642
0.3129366467538759	0.6337576661272735	0.3663664625579273
0.4197383135798205	0.4466139267558802	0.5534212092266557
0.4197256135549736	0.5533250379058856	0.4465383251664790
0.3884285992598748	0.4148625872596341	0.5842929645498884
0.3883480130770423	0.5848652732489228	0.4152146389109177
0.3349124790326883	0.3479799270362562	0.5935450204460579
0.3327347525976947	0.5916622349591094	0.3464879993922949
0.3329713895616493	0.6537762551283706	0.4084254996773993
0.3347561768306946	0.4067265094842440	0.6518933545703050
0.3705549007487505	0.3705948260316063	0.5670060725973514
0.3703593003350178	0.5686902791306068	0.3707700334160293
0.3697581505369030	0.6286975234917449	0.4322946529028329
0.3724257794747103	0.4307576241891979	0.6277473184733324
0.6540717922152979	0.5264039541682521	0.5198714475807407
0.4106558393051927	0.4217572135692649	0.3904573385877157
0.3912782280047864	0.4107094364929225	0.4435652361882632
0.4128480077161996	0.5759406391176327	0.6085416865660420
0.3915017779428851	0.5891287843995627	0.5558068098592486
0.6026691205880681	0.4606974956818419	0.7882846354462671
0.6429092523369775	0.4183916997138226	0.7203854065013573
0.5058975773974694	0.5287112768745246	0.6898120628973317

0.6228986716943794	0.4419235325657169	0.6359666709176892
0.6229309369660783	0.6359598376307076	0.4418957018867999
0.5065796091809602	0.6896554477565999	0.5268566798340970
0.5314311226745971	0.7741851828253559	0.5068664250399095
0.6043834812376829	0.7878264803454584	0.4580727152568969
0.6229278584305860	0.5581269183365123	0.3640722311890698
0.6428630474822299	0.5816679605915525	0.2796791673099932
0.6007890711724002	0.5427783561073767	0.2115237824456742
0.5093563349289596	0.4673046623020340	0.3104457569161383
0.3191701838372509	0.5778359882825939	0.3128187443415342
0.6440135407237619	0.2816105812870759	0.5811605159951796
0.6047791753245519	0.2120700315582269	0.5405043074094777
0.5313779959128766	0.2258627252331351	0.4931557266657833
0.5091130275040783	0.3104343782907151	0.4712726860447205
0.3839441021285808	0.3548490319993078	0.5339625689056788
0.2798459200618808	0.3504219142748539	0.6475504461559609
0.3215025285450750	0.4219802311581944	0.6853861004819564
0.3863487481343668	0.4641242294133434	0.6410444390140029
0.3214227825782245	0.6888612318144141	0.4219853314898145
0.2821297637113460	0.6503016179930796	0.3497955057088191
0.3839141186152446	0.5339098729918419	0.3592685074048418
0.5202304240349633	0.5026384226571423	0.7790002320950506
0.6410229228140167	0.7209612661740863	0.4041993145908478
0.6331570511395341	0.3697753217594837	0.5588203622487171
0.3236264745709647	0.3015334533645415	0.5822069722375289
0.3858472283420085	0.6453362752159109	0.4752977209815796
0.5156478048175117	0.5020126678056513	0.2250594709804830

5. NO_2-MIL-101(Fe) (-725.54852021 eV)

NO_2-MIL-101(Fe)

1.00000000000000

27.9719696044999999	0.0000000000000000	0.0000000000000000
0.0000000000000000	27.9719696044999999	0.0000000000000000
0.0000000000000000	0.0000000000000000	27.9719696044999999

Fe	O	C	H	N
3	28	42	29	6

Direct

0.5789769753921590	0.4989994938855276	0.4989440435907611
0.4755191766732381	0.4574231310338064	0.4583352672237808
0.4764356699877652	0.5409342797464944	0.5425613558410518
0.5047844334247316	0.4998871903123501	0.4999090137034727
0.5826557527272200	0.5738593237337284	0.4975894102101179
0.5824870489567999	0.4977010164085058	0.5738877928218500
0.5825838383463093	0.4260799878487589	0.4976998049809751
0.5824843226830367	0.5022406528026266	0.4260654014282395
0.5153548186171122	0.6005095207686466	0.5291272281516513
0.5131617603373897	0.5269235241720764	0.6026346247131542
0.5130748775232234	0.3972371664992380	0.4730476445076519
0.5133900000998821	0.4716648210594698	0.3973573765446403
0.4306525338752536	0.4308503101021230	0.5114104754510862
0.4308134069974656	0.5114036217214704	0.4265343703087581
0.4307116931118813	0.5645060654325533	0.4886137926091862
0.4324564717414248	0.4859598398125939	0.5708178278630243
0.4167946091186323	0.4237079718163628	0.4241006195904262
0.4173547023578124	0.5738584350054765	0.5739129550478168

0.6431113315990160	0.4998276418141359	0.4998617243383663
0.6451732259504368	0.6898870010707583	0.3685076377573040
0.6673001285078186	0.7593881211459816	0.3992037068719725
0.5358602045217324	0.5090295535702793	0.8216294767943353
0.4738889189530724	0.5039326115043181	0.7729115389085308
0.6765316322986905	0.3592127224225692	0.5647308610622588
0.6181466238228097	0.4086411749387437	0.5738629733406384
0.3546737590320035	0.2745059870611968	0.5646619463782940
0.2809511041901374	0.2855360587805862	0.5893250465629976
0.5358257646586395	0.4909686266195905	0.1783276649250496
0.4739481117732183	0.4949022981975659	0.2270886044038178
0.4307909656556674	0.6497469189941398	0.4841340116375008
0.3569686768739331	0.6697580695820733	0.5001631467066474
0.6119562880906955	0.7124316313525156	0.4420477661479083
0.6141786033788819	0.4441688853002645	0.7124774129794318
0.6139457452909056	0.2857485469237417	0.5569349211377101
0.6142208988999442	0.5557720866001645	0.2875862721457452
0.5915438220915377	0.7520235008192273	0.4659724943235020
0.5914088949288328	0.4663708105783044	0.7516889727374192
0.5914726777126944	0.2478069178332376	0.5339757309512336
0.5916234320866351	0.5338152506811369	0.2481313590882692
0.6024886816963217	0.6653024125295204	0.4554566314013755
0.6025799481503000	0.4556205965783846	0.6654087964584079
0.6015302552211779	0.3324117970645730	0.5430909682748961
0.6024999708179735	0.5442831061467430	0.3346412809766193
0.5537192852938730	0.6072397556520954	0.5061937937732210
0.5534655058489690	0.5045944059254026	0.6072626679569950
0.5535010396023594	0.3924452082667429	0.4931807832558590
0.5534264261293060	0.4937526501056553	0.3926266427557934
0.5649579429282099	0.6579635065004723	0.4887816950168968

0.5648492969267380	0.4886677322379782	0.6578907599971942
0.5645405136602122	0.3419940369924390	0.5111133734410700
0.5647124148551086	0.5111341901722284	0.3420114625942290
0.5514182402458232	0.7433749861468115	0.4937622028032109
0.5491998591705638	0.4911874555662266	0.7411377527346588
0.5510870131114842	0.2566916995671633	0.5062938424606713
0.5510156624533238	0.5067004610201167	0.2571501366293631
0.5381223969599915	0.6965570405385648	0.5048688081381840
0.5359484833396451	0.5043069175977095	0.6948432416347643
0.5359101579428440	0.3034408213200024	0.4954674629031288
0.5359936411195889	0.4954978059162229	0.3036525104650991
0.3107048957686658	0.3683626688846289	0.6321275894074461
0.3108426115225171	0.6317936375375623	0.3681632584873853
0.4196903296336103	0.4463475433336228	0.5535146318234894
0.4197633312672764	0.5510854343172511	0.4462334480226460
0.3881657623098960	0.4149861509705488	0.5846817990879316
0.3883946892419985	0.5847889350569133	0.4151571468941526
0.3347787678762657	0.3482061317838188	0.5936176205862012
0.3326726451043598	0.5915429085051542	0.3464817678220840
0.3325235114819236	0.6535633187704804	0.4082397606071524
0.3327420169812996	0.4083891212957980	0.6535332484406666
0.3706386746923940	0.3706958482156608	0.5671025997603181
0.3703572193351690	0.5685898387118087	0.3708158129248176
0.3704608527210169	0.6293837163698583	0.4303394152366877
0.3704613759083415	0.4312647850218951	0.6292170801570194
0.6563157792884411	0.5246405632648177	0.5201801457376760
0.4106566007547698	0.4217539507474442	0.3904515441776155
0.3912700852315965	0.4106777109747100	0.4435586598000114
0.4128674990521546	0.5759159518028056	0.6085504041142684
0.3913664085105282	0.5892833504754194	0.5564451591911563

0.6045411875579916	0.4638153430810448	0.7880590875433882
0.6429014612775035	0.4183354348060533	0.7203652220520667
0.5065766179431321	0.5285121712572760	0.6876042556174440
0.6241331343927641	0.4434523716277994	0.6355635625281408
0.6228764787014711	0.6356041381703790	0.4415553559850949
0.5087613522256049	0.6896633984027432	0.5290689261410080
0.5314341367815985	0.7741567572870451	0.5068639488688049
0.6048468434976897	0.7878844162838448	0.4594544316839233
0.6234973156362216	0.5567954203065142	0.3649100816987172
0.6429149089231387	0.5815754970568250	0.2796458230838823
0.6044155212720668	0.5401490070571455	0.2119572212806133
0.5061033036440463	0.4713378877505363	0.3098738821410691
0.3212886637816723	0.5780843408087080	0.3111864836576288
0.6427624143995843	0.2791383189439287	0.5822121661908142
0.6048719517868122	0.2115065187409684	0.5384404393237645
0.5314319749015155	0.2257587301507655	0.4931277790660431
0.5062163777192623	0.3102468825319065	0.4705934634490347
0.3839642390203082	0.3548511914592041	0.5339961818989494
0.2795608009479835	0.3503721229874728	0.6473027954113846
0.3234891043671482	0.4199780482111493	0.6894762484557759
0.3840399834138495	0.4657873585531931	0.6412982048652864
0.3214266668991002	0.6888036379367293	0.4219278750154132
0.2813064651668427	0.6496661270283042	0.3503127134841648
0.3839091229237539	0.5338775957223234	0.3592251288029757
0.5178174132334092	0.5043907726474330	0.7812847478163647
0.6449621102391916	0.7210153056796855	0.4000199332431933
0.5178387070548638	0.4955465971311384	0.2186661584486558
0.6339660482156617	0.3703806457017507	0.5624296346511954
0.3214960233417017	0.2991563653240803	0.5803721377889772
0.3883659702894704	0.6518107055163966	0.4733288270693237

6. CF3-MIL-101(Fe) (-739.03597101 eV)

```
 CF3-MIL-101(Fe)
 1.00000000000000
   27.9719696044999999    0.0000000000000000    0.0000000000000000
    0.0000000000000000   27.9719696044999999    0.0000000000000000
    0.0000000000000000    0.0000000000000000   27.9719696044999999
  Fe   O    C    H    F
    3   16   48   29   18
Direct
  0.5784896926721146  0.5008574098124967  0.4985335830205528
  0.4749300265152669  0.4582309223949679  0.4580490787021390
  0.4758144362404266  0.5413237723939446  0.5425530780220100
  0.5060232640787916  0.4999908256387400  0.4999890978822421
  0.5826616772594164  0.5739008442180022  0.4976313352552353
  0.5825108094124687  0.4976940178064311  0.5738948502235104
  0.5760071735780272  0.4284917036821696  0.4952833292014276
  0.5824499145447675  0.5022632388195944  0.4261222346147591
  0.5153620818696822  0.6005332397199865  0.5291301860963600
  0.5131626237508371  0.5269262308288845  0.6026252318016390
  0.5069716781099842  0.3950329174092886  0.4710473020364887
  0.5133619383454899  0.4716623486947142  0.3973700235234290
  0.4306940235218804  0.4308779175985151  0.5114359095327146
  0.4307465949700600  0.5114680833104615  0.4308119987111567
  0.4309638279423922  0.5683087267415132  0.4882821294009361
  0.4307752479548839  0.4884955006002940  0.5691431756787608
  0.4155002341855791  0.4240812086528507  0.4241717427007217
  0.4174195630898296  0.5738176098209422  0.5739659430412445
  0.6431180125381033  0.4998752355459501  0.4999037992578224
  0.6135071039058602  0.7124896736943853  0.4442385452259288
  0.6142971154778394  0.4439771366799121  0.7121629090489989
```

0.6140290801161115	0.2876969962187488	0.5553813361337774
0.6143285716324769	0.5559360254177292	0.2878832820216459
0.5914278342314248	0.7518438976584676	0.4662292574417535
0.5893902550179817	0.4641713028610823	0.7500773060203372
0.5915803426147335	0.2480536761390582	0.5339588219027931
0.5894424723175007	0.5357955489866271	0.2499879869800452
0.6024153865667117	0.6654048077527221	0.4558273592562330
0.6025228180249584	0.4556341730850875	0.6653717815193474
0.6024056344803057	0.3345817698110523	0.5420778823839285
0.6025649548176588	0.5442098914626072	0.3347769021651104
0.5536666822801877	0.6072827464521851	0.5061839106874828
0.5534618110140315	0.5045836834187583	0.6072589150166081
0.5489627571629256	0.3928680075285413	0.4910906038563922
0.5536555065032758	0.4929679533474243	0.3928567084136532
0.5648627494436980	0.6579451488863555	0.4888171213562558
0.5648631594774969	0.4886469208460085	0.6578392932506958
0.5633299805526661	0.3437419440049254	0.5110427336683614
0.5669605146134487	0.5090634811448780	0.3437589176724387
0.5513211331926158	0.7433503744649812	0.4938068137285043
0.5492665735444930	0.4911407318944114	0.7407859140051869
0.5513317881564532	0.2566349861885584	0.5062476159969194
0.5511030358683442	0.5067966484994102	0.2607275970836156
0.5376222380679323	0.6965623087933963	0.5046647500937596
0.5360518020403262	0.5044090103607317	0.6947048243800609
0.5376756690327369	0.3034758500185148	0.4953691111528400
0.5384959333147421	0.4931873163796610	0.3060683332627718
0.3108327806833842	0.3682990648111115	0.6319160205217287
0.3127649804174268	0.6337745899713241	0.3661804393239265
0.4197356563227288	0.4466257211340547	0.5534095433175565
0.4198131817168758	0.5532095879492118	0.4465934228710395

0.3885139966187197	0.4149229775731911	0.5842458015765857
0.3882451301490022	0.5846963168973645	0.4150288099814929
0.3349374625983828	0.3482916299520582	0.5935110545493174
0.3327273590840605	0.5916613873787924	0.3464898393920990
0.3325899584316474	0.6536554830903825	0.4081256129257724
0.3346929763115156	0.4067600502270068	0.6519651901335237
0.3706432383219465	0.3707439512979533	0.5670805036483415
0.3703876350859261	0.5684836591777724	0.3707326017323425
0.3685052909831867	0.6275699765126390	0.4312370223493787
0.3724052456454530	0.4307298647099103	0.6277079696036836
0.5195931042346089	0.5040275957081519	0.7810342847424465
0.6432395731724014	0.7228935820545885	0.4020757878540380
0.6365408525291087	0.3707552158998908	0.5616624558826473
0.3233054757246805	0.2992997814097649	0.5807624060245615
0.3880611884363816	0.6474738285517176	0.4774058517821871
0.5152034323564649	0.4998617483731138	0.2232666373533903
0.6541220252784967	0.5263876095999791	0.5198501134720317
0.4104008161778268	0.4212639181381874	0.3904330748592955
0.3905451417920105	0.4106698915081211	0.4437513114591383
0.4129245742955661	0.5757669738254378	0.6085443362681389
0.3915414121639600	0.5893283609347932	0.5558909890511075
0.6002465546553850	0.4589822129634697	0.7873631118551003
0.6428680074200201	0.4181480165387228	0.7203157917792566
0.5066158646549255	0.5285349227006293	0.6875425825223473
0.6229221229300137	0.4419663273226675	0.6359562013691189
0.6228916224875078	0.6359465187418181	0.4419039549598196
0.5065765926368186	0.6896194227410746	0.5268194507900006
0.5313497991558762	0.7741298225395425	0.5068276869093964
0.6026918662593914	0.7880566388104526	0.4575138230561038
0.6229478551952781	0.5580809895262391	0.3640915751401508

0.6428427786313407	0.5818524879860902	0.2797239247097068
0.5994654403132600	0.5433012368706969	0.2128728635550132
0.5110207041327541	0.4669476249183901	0.3125468127392423
0.3191949490596002	0.5778098782675016	0.3127971365763003
0.6445370293300314	0.2828563092236251	0.5794635365641696
0.6047590908192859	0.2119470239725203	0.5403290807333221
0.5314024493952871	0.2258174320007882	0.4931410743981388
0.5086568259999211	0.3102086551202561	0.4709490012659430
0.3839913263164618	0.3548927673138564	0.5340105542439915
0.2796523604360814	0.3504474986327821	0.6473762074240668
0.3215250041210780	0.4219971327008523	0.6853975130393977
0.3863666606857947	0.4641015833789339	0.6410121140845959
0.3214415479883073	0.6888163534346461	0.4219115916688239
0.2826470647048467	0.6506141860339625	0.3492485381007455
0.3838893911859955	0.5338003759997605	0.3591570873153600
0.4978253809993944	0.5424429046925283	0.2071543076741804
0.5330679603424926	0.4753887680421514	0.1851502796495978
0.4768565585503097	0.4768432611149578	0.2417270436783099
0.3725706473154986	0.6187177323393129	0.5155874889622254
0.4353042668224631	0.6540957587971334	0.4758815138052697
0.3683182641885026	0.6901765400309685	0.4888396173995133
0.3326505114145277	0.2696503359855313	0.6183606711344751
0.2764041532686861	0.2944838515984856	0.5712682323238027
0.3490551965457840	0.2847523033542103	0.5436854150424466
0.6879140147718132	0.7053747260272161	0.4106539863122549
0.6272070559866947	0.6986631892053197	0.3633891371282303
0.6455156170062111	0.7700007140033094	0.3905969050977305
0.5044095746252424	0.4639883095016870	0.8038846553896377
0.5430407248091527	0.5314340926923862	0.8127923153513663
0.4796487815555821	0.5249566594093409	0.7656637425546506

0.6406961502069777	0.3680317816429763	0.6097587432530176
0.6810001464796542	0.3634557261831333	0.5424297035784704
0.6258180160149749	0.4160311812675630	0.5543716337604252

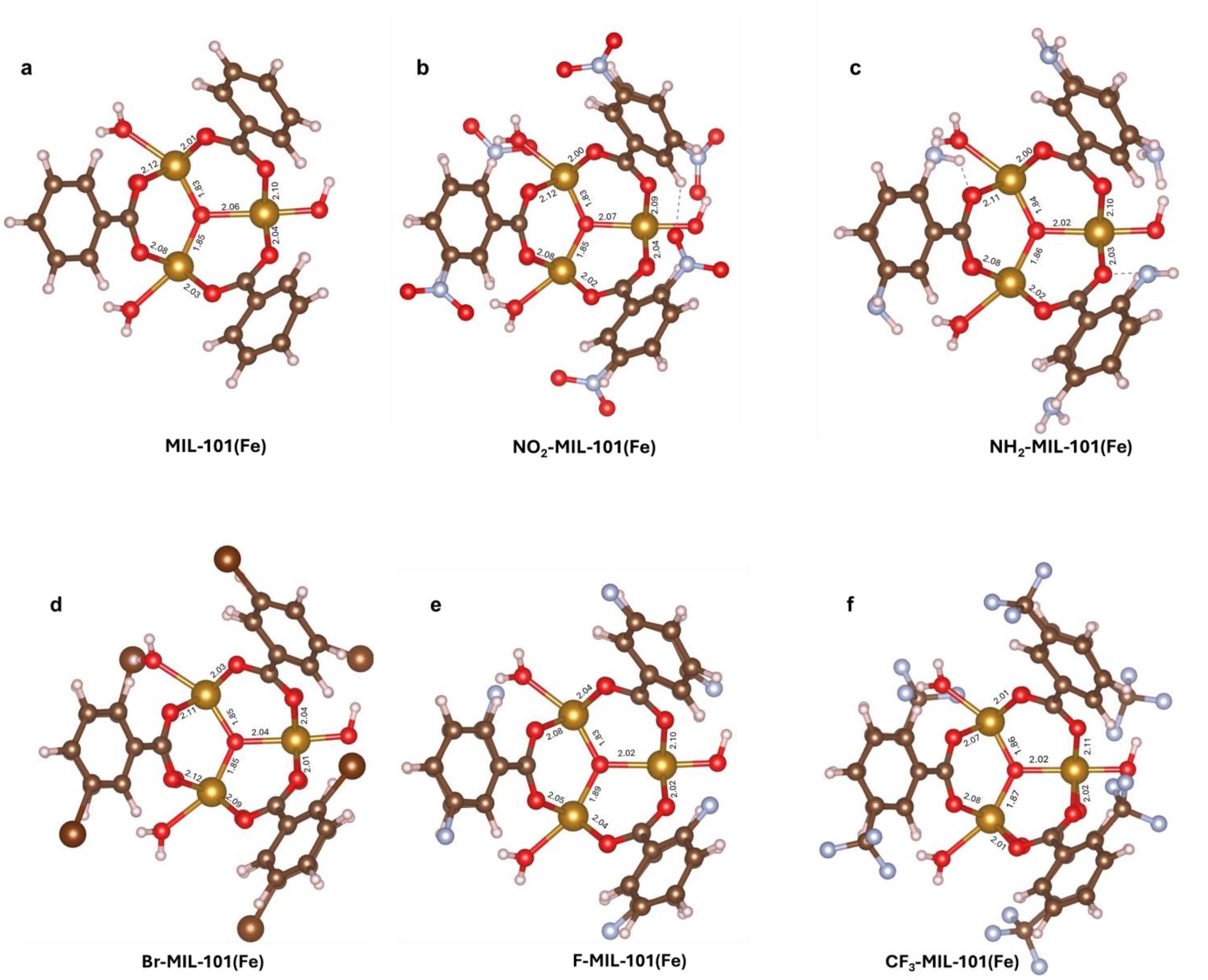

Figure S39. DFT cluster structures of single-ligand MIL-101(Fe) derivatives, showing selected bond lengths (Å). Distances include Fe–central O bonds and Fe–O(COO) coordination bonds for all samples.

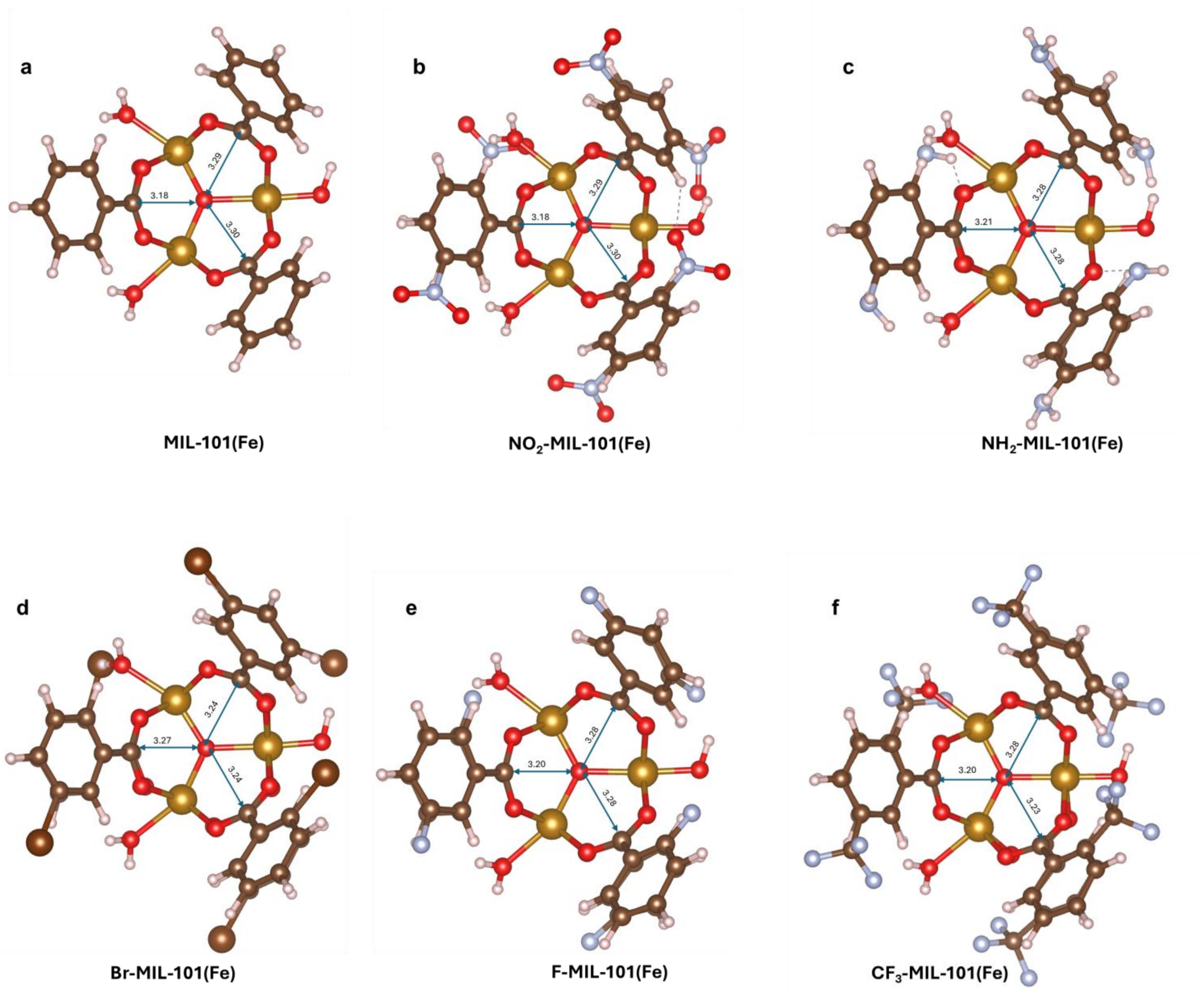

Figure S40. DFT cluster structures of single-ligand MIL-101(Fe) derivatives, showing selected distances (Å). Distances correspond to the central O atom to C atom of the carboxylate (COO^-) coordination for all samples.

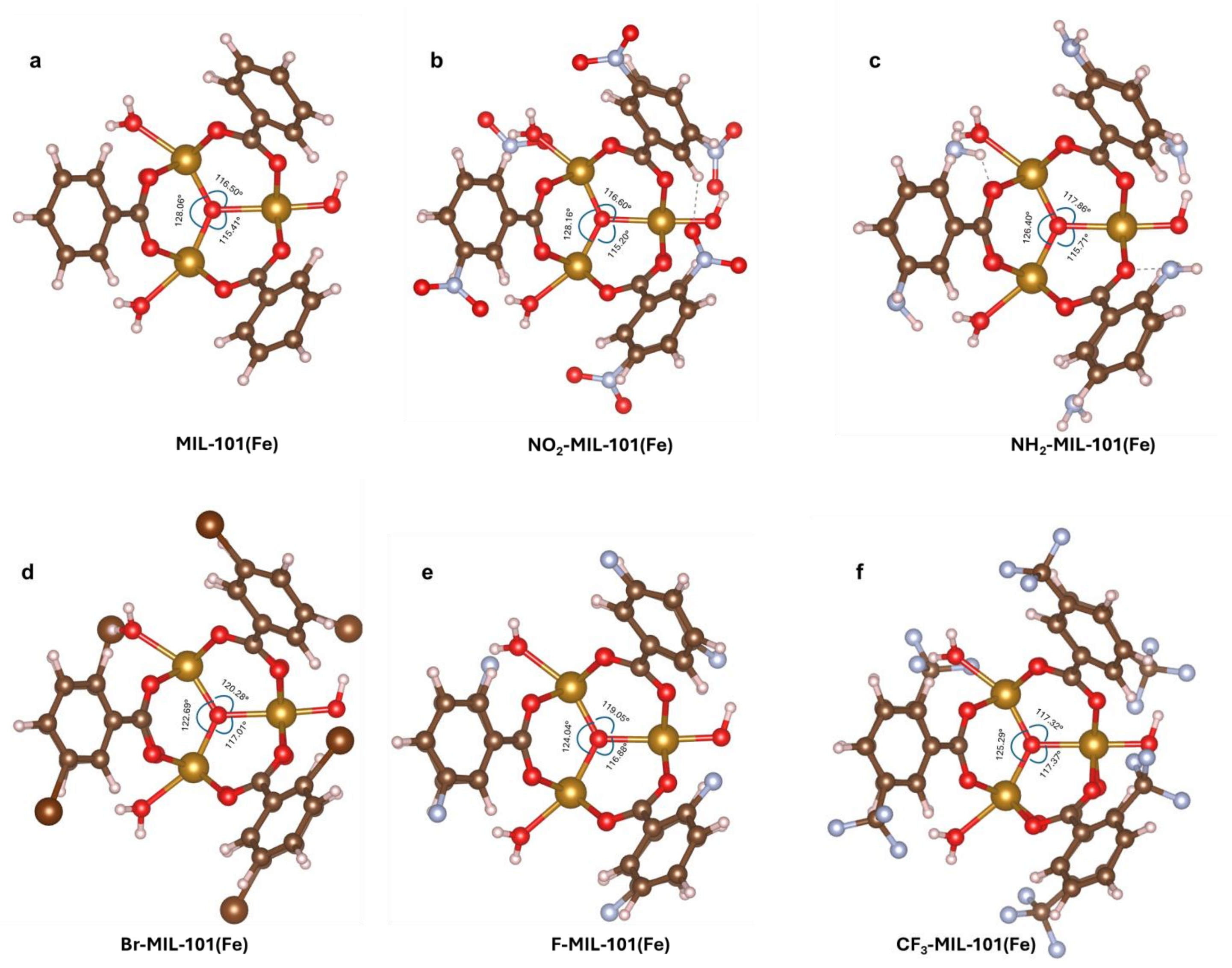

Figure S41. DFT cluster structures of single-ligand MIL-101(Fe) derivatives, showing selected bond angles (°) for all samples.

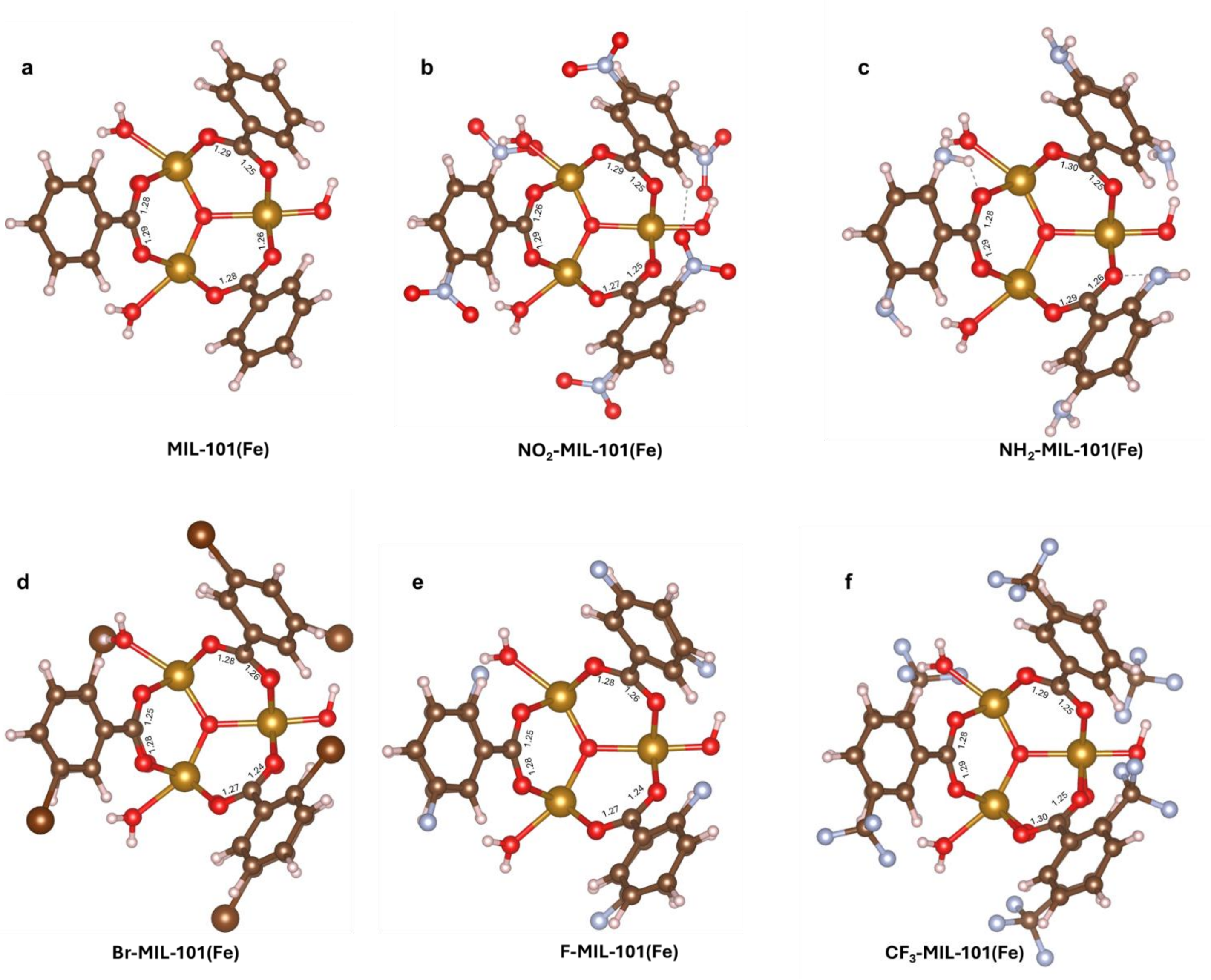

Figure S42. DFT cluster structures of single-ligand MIL-101(Fe) derivatives, showing selected bond lengths (Å). Distances correspond to C–O coordination bonds for all samples.

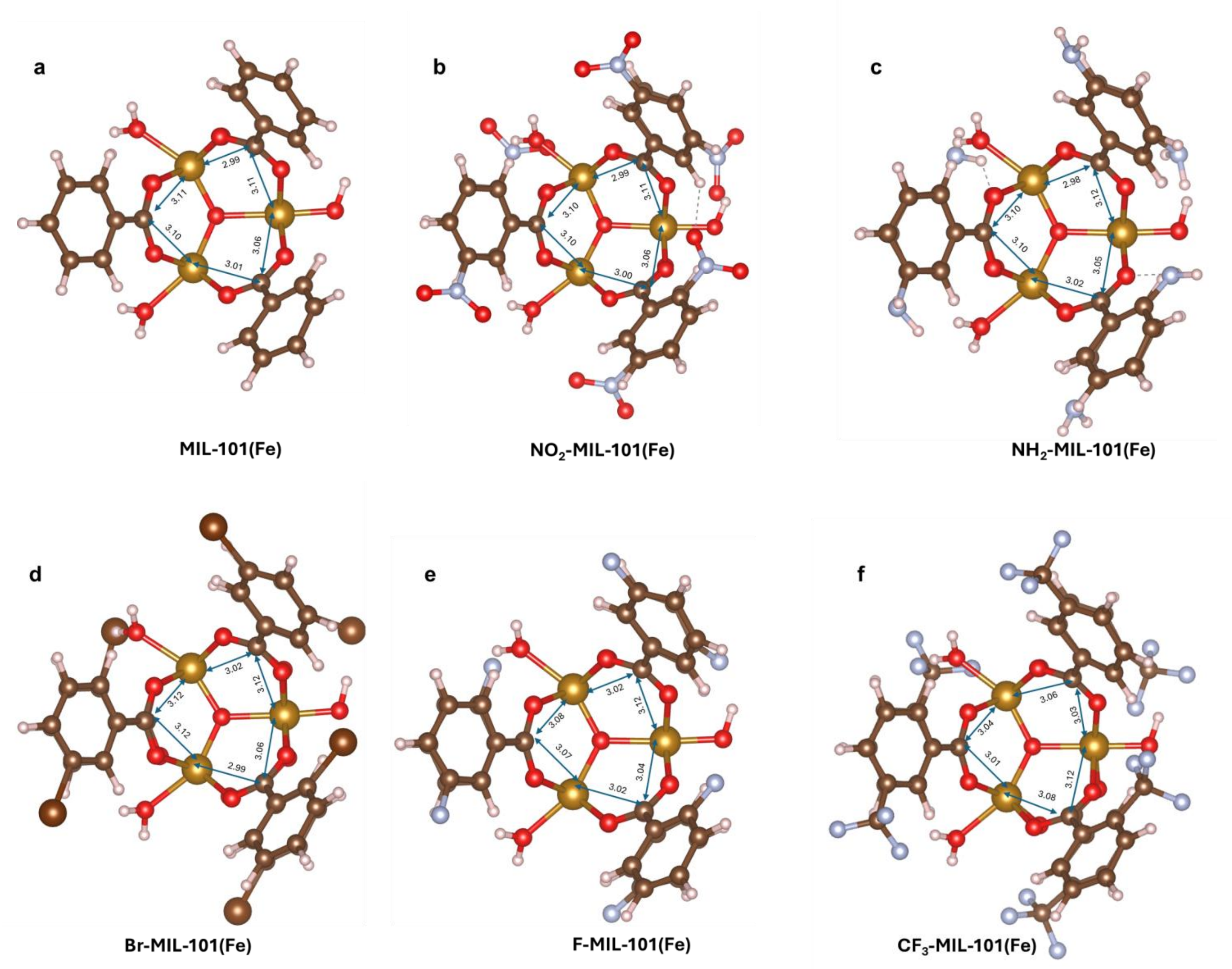

Figure S43. DFT cluster structures of single-ligand MIL-101(Fe) derivatives, showing selected distances (Å) corresponding to Fe–C from carboxylate (COO^-) coordination.

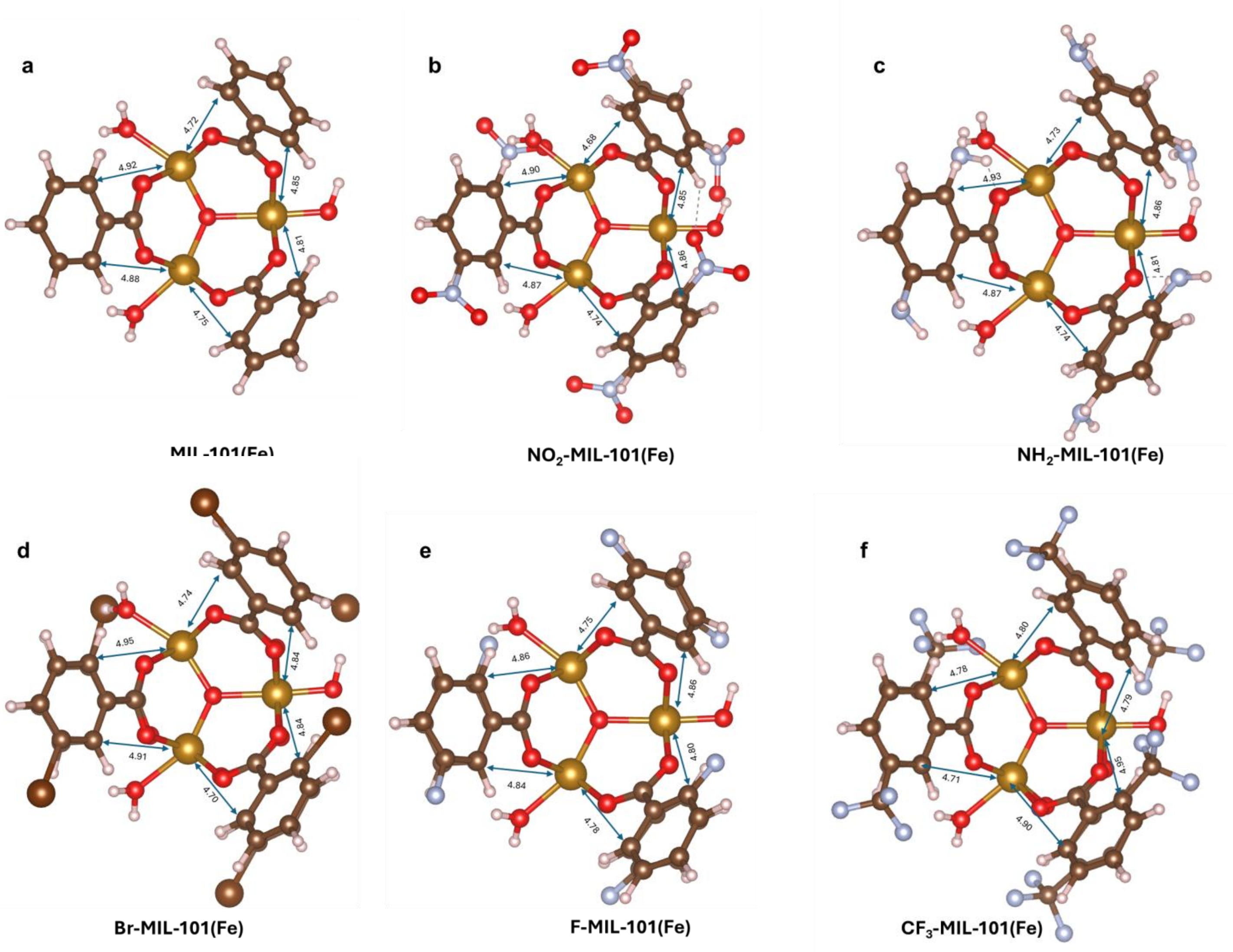

Figure S44. DFT cluster structures of single-ligand MIL-101(Fe) derivatives, showing selected distances (Å) corresponding to Fe–C distances to the carbon atoms of the benzene ring.

S6. SUPPLEMENTARY TABLES

Table S1. Actual molar ratios of NH_2-BDC to BDC ligands in mixed-ligand MOFs based on peak integration variation calculated by ^{1}H-NMR.

Synthesis NH_2-BDC content (mol.%)	Actual content (mol.%)
**2**	2.5
**5**	4.7
**17**	16.6
**50**	28.6

Table S2. Specific surface area and porosity parameters.

Sample	BET S_A ($m^2\ g^{-1}$)	Total pore volume ($cm^3\ g^{-1}$)	Micropore volume ($cm^3\ g^{-1}$)	Micropore area ($m^2\ g^{-1}$)
**MIL-101(Fe)**	2845	1.688	1.480	2700
**F-MIL-101(Fe)**	2512	1.681	1.462	2338
**NO_2-MIL-101(Fe)**	1779	1.147	0.978	1669
**Br-MIL-101(Fe)**	1822	0.993	0.912	1772
**NH_2-MIL-101(Fe)**	1936	1.073	0.947	1845
**2% NH_2-MIL-101(Fe)**	2348	1.622	1.396	2196
**5% NH_2-MIL-101(Fe)**	2174	1.365	1.188	1961
**17% NH_2-MIL-101(Fe)**	1971	1.180	1.043	1872
**29% NH_2-MIL-101(Fe)**	1942	1.207	1.018	1812
**CF_3-MIL-101(Fe)**	1536	0.887	0.746	1435
**MIL-88B(Fe)**	564	0.594	0.152	285
**F-MIL-88B(Fe)**	1510	1.074	0.626	1196

Table S3. Control photocatalytic experiments over MIL-101(Fe) under standard reaction conditions[a].

Catalyst	Atmosphere	Solvent	Light	NH_4^+
**No**	N_2	H_2O	Yes	N.D.[b]
**Yes**	Ar	H_2O	Yes	N.D.
**Yes**	N_2	H_2O	No	N.D.

[a] Reaction conditions: catalyst (5 mg), light intensity (23 mW cm^{-2}), solvent (10 mL), reaction time (1h). [b] N.D. = not detected.

Table S4. Hydrogen evolution reaction experiments[a].

Sample	NH_4^+
**MIL-101(Fe)**	N.D.[b]
**F-MIL-101(Fe)**	N.D.
**NO_2-MIL-101(Fe)**	N.D.
**Br-MIL-101(Fe)**	N.D.
**NH_2-MIL-101(Fe)**	N.D.
**2% NH_2-MIL-101(Fe)**	N.D.
**5% NH_2-MIL-101(Fe)**	N.D.
**17% NH_2-MIL-101(Fe)**	N.D.
**29% NH_2-MIL-101(Fe)**	N.D.
**CF_3-MIL-101(Fe)**	N.D.
**MIL-88B(Fe)**	N.D.
**F-MIL-88B(Fe)**	N.D.

Table S5. ʒ-Potential as average value of six measurements with denoted pH values.

Sample	pH	ʒ-Potential (mV)
**MIL-101(Fe)**	3.5	+27 ± 0.4
**F-MIL-101(Fe)**	3.2	+19.9 ± 0.4
**NO_2-MIL-101(Fe)**	3.1	+6.3 ± 0.1
**Br-MIL-101(Fe)**	3.2	+17.2 ± 0.3
**NH_2-MIL-101(Fe)**	3	+28.3 ± 2.9
**CF_3-MIL-101(Fe)**	3.2	+ 19.1 ± 0.4
**MIL-88B(Fe)**	3.5	+24.3 ± 0.8
**F-MIL-88B(Fe)**	3.2	+ 18.6 ± 0.2

Table S7. Fitted time constants for different MOFs at representative wavenumbers based on TRIR measurements.

Sample	a_1 (.10^{-5})	t_1 (μs)	a_2 (·10^{-5})	t_2 (μs)	<t> (ns)	Ref.
**MIL-101(Fe)**	-7.15	28.8	-	-	28.8	[22]
**NH_2-MIL-101(Fe)**	-3.50	5.2	-4.7	17. 7	12.4	[22]
**NO_2-MIL-101(Fe)**	-10.2	19.7	-10.7	113.8	67.9	[22]
**F-MIL-101(Fe)**	-3.2	11.0	-2.5	57.9/64.3	32.1	This work
**Br-MIL-101(Fe)**	-2.8	10.2	-2.5	51.5/62.6	29.7	This work

Table S8. Fe leaching content as average value of three measurements with relative standard deviations.

Sample	Fe in ppb = µg/L	RSD (%)	Fe content (%)
**MIL-101(Fe)**	3290.46	0.6	1.43
**F-MIL-101(Fe)**	2497.18	1.2	1.16
**NO_2-MIL-101(Fe)**	3395.59	0.4	1.75
**Br-MIL-101(Fe)**	2660.94	0.7	1.53
**NH_2-MIL-101(Fe)**	2810.06	0.4	1.30
**2% NH_2-MIL-101(Fe)**	2397.60	0.6	1.04
**5% NH_2-MIL-101(Fe)**	1898.87	0.4	0.82
**17% NH_2-MIL-101(Fe)**	3541.99	1.1	1.56
**29% NH_2-MIL-101(Fe)**	2941.14	0.4	1.30
**Fe_3O-Bz complex**	5703.26	0.4	3.76

Table S9: Bader charge (eV) on Fe ions in MIL-101(Fe) with and without central oxygen.

Material	MIL-101(Fe)	MIL-101(Fe) without central oxygen
**Fe1**	-1.79	-1.65
**Fe2**	-1.85	-1.36
**Fe3**	-1.84	-1.36

Table S10. Central O–Fe bond lengths (Å) for MIL-101(Fe) and all single-ligand derivatives (F, CF_3, Br, NO_2, NH_2).

MOF	NO_2	NH_2	Br-	F	CF_3
1.85	1.85	1.86	1.85	1.89	1.87
1.83	1.83	1.84	1.85	1.83	1.86
2.06	2.07	2.02	2.04	2.02	2.02

Table S11. Fe–O(COO) bond lengths (Å) for MIL-101(Fe) and all single-ligand derivatives.

MOF	NO_2	NH_2	Br-	F	CF_3
2.08	2.08	2.12	2.12	2.05	2.08
2.12	2.12	2.11	2.11	2.08	2.07
2.01	2.00	2.00	2.03	2.04	2.01
2.10	2.09	2.10	2.04	2.10	2.11
2.04	2.04	2.03	2.01	2.02	2.02
2.03	2.02	2.02	2.09	2.04	2.01

Table S12. Central O–C(COO) distances (Å) for MIL-101(Fe) and all single-ligand derivatives.

MOF	NO_2	NH_2	Br-	F	CF_3
3.18	3.18	3.21	3.27	3.20	3.20
3.29	3.29	3.28	3.24	3.28	3.28
3.30	3.30	3.28	3.24	3.28	3.23

Table S13. Fe–central O–Fe angles (°) for MIL-101(Fe) and all single-ligand derivatives.

MOF	NO_2	NH_2	Br-	F	CF_3
128.06	128.16	126.40	122.69	124.04	125.29
116.50	116.60	117.86	120.28	119.05	117.32
115.41	115.20	115.71	117.01	116.88	117.37

Table S14. C–O(COO) (coordination bond lengths) (Å) for MIL-101(Fe) and all single-ligand derivatives.

MOF	NO_2	NH_2	Br-	F	CF_3
1.29	1.29	1.29	1.28	1.29	1.29
1.28	1.26	1.28	1.25	1.28	1.28
1.29	1.29	1.30	1.28	1.29	1.29
1.25	1.25	1.25	1.26	1.25	1.25
1.26	1.25	1.26	1.24	1.26	1.25
1.28	1.27	1.29	1.27	1.27	1.30

Table S15. Fe to C of COO distance (Å) for MIL-101(Fe) and all single-ligand derivatives.

MOF	NO_2	NH_2	Br-	F	CF_3
3.10	3.10	3.10	3.12	3.07	3.09
3.11	3.10	3.10	3.12	3.08	3.09
2.99	2.99	2.98	3.02	3.02	3.00
3.11	3.11	3.12	3.12	3.12	3.12
3.06	3.06	3.05	3.06	3.04	3.13
3.01	3.00	3.02	2.99	3.02	2.91

Table S16. Fe to C in benzene ring distance (Å) for MIL-101(Fe) and all single-ligand derivatives.

MOF	NO_2	NH_2	Br-	F	CF_3
4.88	4.87	4.87	4.91	4.84	4.87
4.92	4.90	4.93	4.95	4.86	4.85
4.72	4.68	4.73	4.74	4.75	4.69
4.85	4.85	4.86	4.84	4.86	4.88
4.81	4.86	4.81	4.84	4.80	4.85
4.75	4.74	4.74	4.70	4.78	4.78

Table S17: Comparison of NH_3 synthesis performance for Fe-based MOFs reported in the literature and this work. Broader summaries of MOF systems can be found in recent reviews[23,24,25].

Catalyst	Concentrations	Scavenger	Light source	Detection method	NH_3 evolution rate/μmol h^{-1} g^{-1}	Ref.
**MIL-53(FeII/FeIII)-0.1**	10 mg in 100 mL H_2O	K_2SO_4	300 W Xe lamp (λ > 420 nm)	Ion chromatography	306	26
**MIL-101(FeII/FeIII)**	50 mg in 100 mL H_2O	none	300 W Xe lamp (λ > 420 nm)	Nessler's reagent method	466.8	27
**Cu doped MIL-101(Fe)**	50 mg in 100 mL H_2O	none	300 W Xe lamp (full spectrum)	Nessler's reagent method	315.2	28
**HMOF(FeIII/FeII)-2.5 (MOF-235(Fe))**	10 mg in 100 mL H_2O	K_2SO_4	300 W Xenon lamp (full spectrum)	Ion chromatography	164	29
**F-MIL-101(Fe)**	5 mg in 10 mL H_2O		150 W Xe lamp (λ > 420 nm)	Nessler's reagent method	702	This work

References

(1) Li, S.; Sun, S.; Wu, H.; Wei, C.; Hu, Y. Effects of Electron-Donating Groups on the Photocatalytic Reaction of MOFs. Catal. Sci. Technol. 2018, 8 (6), 1696–1703. https://doi.org/10.1039/C7CY02622F.

(2) Li, D.; Hao, Y.; Zhou, H.; Ma, W.; Zhang, N.; Lu, M. Construction of Ionic Liquid Modified MIL-88(Fe)–NH2 for Efficient Separation and Enrichment of Bisphenols Contaminants in Water and Honey. Chemical Engineering Journal 2025, 507, 160784. https://doi.org/10.1016/j.cej.2025.160784.

(3) Ye, Q.; Cairnie, D. R.; Troya, D.; Kumar, N.; Yang, X.; Morris, A. J. Photoinduced Dynamic Ligation in Metal–Organic Frameworks. J. Am. Chem. Soc. 2024, 146 (1), 101–105. https://doi.org/10.1021/jacs.3c12217.

(4) Zhang, L.; Chen, R.; Liu, Y.; Ding, C.; Wang, Y.; Chen, X.; Ji, Y.; Liu, Y.; Hang, X. Heterogeneous Fenton Degradation of Enrofloxacin over Quinone-Modified MIL-101: Density Functional Theory Calculation and Mechanism Insight. Journal of Environmental Chemical Engineering 2025, 13 (3), 116817. https://doi.org/10.1016/j.jece.2025.116817.

(5) Kresse, G.; Furthmüller, J. Efficiency of Ab-Initio Total Energy Calculations for Metals and Semiconductors Using a Plane-Wave Basis Set. Computational Materials Science 1996, 6 (1), 15–50. https://doi.org/10.1016/0927-0256(96)00008-0.

(6) Kresse, G.; Furthmüller, J. Efficient Iterative Schemes for Ab Initio Total-Energy Calculations Using a Plane-Wave Basis Set. Phys. Rev. B 1996, 54 (16), 11169–11186. https://doi.org/10.1103/PhysRevB.54.11169.

(7) Kresse, G.; Hafner, J. Ab Initio Molecular Dynamics for Liquid Metals. Phys. Rev. B 1993, 47 (1), 558–561. https://doi.org/10.1103/PhysRevB.47.558.

(8) Vitillo, J. G.; Lu, C. C.; Cramer, C. J.; Bhan, A.; Gagliardi, L. Influence of First and Second Coordination Environment on Structural Fe(II) Sites in MIL-101 for C–H Bond Activation in Methane. ACS Catal. 2021, 11 (2), 579–589. https://doi.org/10.1021/acscatal.0c03906.

(9) Liu, Z.; Su, R.; Sun, X.; Zhou, W.; Gao, B.; Yue, Q.; Li, Q. The Obvious Advantage of Amino-Functionalized Metal-Organic Frameworks: As a Persulfate Activator for Bisphenol F Degradation. Science of The Total Environment 2020, 741, 140464. https://doi.org/10.1016/j.scitotenv.2020.140464.

(10) Gecgel, C.; Simsek, U. B.; Turabik, M.; Ozdemir, S. Synthesis of Titanium Doped Iron Based Metal–Organic Frameworks and Investigation of Their Biological Activities. J Inorg Organomet Polym 2020, 30 (3), 749–757. https://doi.org/10.1007/s10904-019-01329-3.

(11) Geng, N.; Chen, W.; Xu, H.; Ding, M.; Lin, T.; Wu, Q.; Zhang, L. Insights into the Novel Application of Fe-MOFs in Ultrasound-Assisted Heterogeneous Fenton System: Efficiency, Kinetics and Mechanism. Ultrasonics Sonochemistry 2021, 72, 105411. https://doi.org/10.1016/j.ultsonch.2020.105411.

(12) Mehar U Nisa; Chen, Y.; Li, X.; Li, Z. Highly Efficient Iron Based MOFs Mediated Catalysts for Fischer–Tropsch Synthesis: Effect of Reduction Atmosphere. Journal of the Taiwan Institute of Chemical Engineers 2020, 107, 44–53. https://doi.org/10.1016/j.jtice.2019.10.025.

(13) Férey, G.; Mellot-Draznieks, C.; Serre, C.; Millange, F.; Dutour, J.; Surblé, S.; Margiolaki, I. A Chromium Terephthalate-Based Solid with Unusually Large Pore Volumes and Surface Area. Science 2005, 309 (5743), 2040–2042. https://doi.org/10.1126/science.1116275.

(14) Taylor, J. The High Temperature Synthesis of Transition Metal Oxo-Carboxylate Polynuclear Complexes. Master's Thesis, Victoria University of Wellington, 2007.

(15) Pazhand, H.; Alvani, A. A. S.; Sameie, H.; Salimi, R.; Poelman, D. Exploring the Effect of Morphologies of Metal Organic Framework MIL-53(Fe) on the Photocatalytic Performance. September 16, 2022. https://doi.org/10.21203/rs.3.rs-2025694/v1.

(16) Cao, W.; Jiang, Z.; Gai, C.; Barrón, V.; Torrent, J.; Zhong, Y.; Liu, Q. Re-Visiting the Quantification of Hematite by Diffuse Reflectance Spectroscopy. Minerals 2022, 12 (7), 872. https://doi.org/10.3390/min12070872.

(17) Vuong, G.-T.; Pham, M.-H.; Do, T.-O. Synthesis and Engineering Porosity of a Mixed Metal Fe2 Ni MIL-88B Metal–Organic Framework. Dalton Trans. 2013, 42 (2), 550–557. https://doi.org/10.1039/C2DT32073H.

(18) Vuong, G.-T.; Pham, M.-H.; Do, T.-O. Direct Synthesis and Mechanism of the Formation of Mixed Metal Fe2Ni-MIL-88B. CrystEngComm 2013, 15 (45), 9694. https://doi.org/10.1039/c3ce41453a.

(19) Lobanov, S. S.; Hsu, H.; Lin, J.; Yoshino, T.; Goncharov, A. F. Optical Signatures of Low Spin Fe3+ in NAL at High Pressure. JGR Solid Earth 2017, 122 (5), 3565–3573. https://doi.org/10.1002/2017JB014134.

(20) Musho, T.; Li, J.; Wu, N. Band Gap Modulation of Functionalized Metal–Organic Frameworks. Phys. Chem. Chem. Phys. 2014, 16 (43), 23646–23653. https://doi.org/10.1039/C4CP03110E.

(21) Liu, N.; Xu, J.; Zhai, Y.; Zhang, Z.; Dang, Y.; Cao, Y.; Li, Z.; Huang, W.; Zhang, X.; Tang, L. Structure–Activity Relationship in Periodate Activation by Fe–MOFs: Why MIL-101(Fe) Outperforms Other MIL-Series in Antibiotic Degradation. Green Energy & Environment 2025, S2468025725002626. https://doi.org/10.1016/j.gee.2025.10.006.

(22) Ye, Q.; Herlinger, I. A.; Fredin, L. A.; Cairnie, D. R.; Yang, X.; Yan, M.; Morris, A. J. Tuning the Lifetimes of Photoinduced Deligation in a Metal–Organic Framework via Linker Functionalization. J. Am. Chem. Soc. 2025, 147 (38), 34690–34696. https://doi.org/10.1021/jacs.5c09921.

(23) Hu, T.; Cheng, X.; Luo, J.; Yan, Y.; Zhang, Q.; Li, Y. Fe-Based Materials for Photocatalytic Nitrogen Reduction to Ammonia: Unique Advantages, Challenges, and Perspectives. ACS Catal. 2024, 14 (19), 14539–14563. https://doi.org/10.1021/acscatal.4c03431.

(24) Zhao, J.; Ren, G.; Meng, X. Recent Advances on MOFs for Photocatalytic and Electrocatalytic Nitrogen Reduction to Produce Ammonia. Nano Energy 2024, 130, 110109. https://doi.org/10.1016/j.nanoen.2024.110109.

(25) Fan, L.; Yu, Q.; Chen, J.; Khan, U.; Wang, X.; Gao, J. Achievements and Perspectives in Metal–Organic Framework-Based Materials for Photocatalytic Nitrogen Reduction. Catalysts 2022, 12 (9), 1005. https://doi.org/10.3390/catal12091005.

(26) Zhao, Z.; Yang, D.; Ren, H.; An, K.; Chen, Y.; Zhou, Z.; Wang, W.; Jiang, Z. Nitrogenase-Inspired Mixed-Valence MIL-53(FeII/FeIII) for Photocatalytic Nitrogen Fixation. Chemical Engineering Journal 2020, 400, 125929. https://doi.org/10.1016/j.cej.2020.125929.

(27) Guo, L.; Li, F.; Liu, J.; Jia, Z.; Li, R.; Yu, Z.; Wang, Y.; Fan, C. Improved Visible Light Photocatalytic Nitrogen Fixation Activity Using a FeII -Rich MIL-101(Fe): Breaking the Scaling Relationship by Photoinduced FeII /FeIII Cycling. Dalton Trans. 2022, 51 (34), 13085–13093. https://doi.org/10.1039/D2DT01215D.

(28) Zhang, Z.; Li, F.; Li, G.; Li, R.; Wang, Y.; Wang, Y.; Zhang, X.; Zhang, L.; Li, F.; Liu, J.; Fan, C. Cu-Doped MIL-101(Fe) with Enhanced Photocatalytic Nitrogen Fixation Performance. Journal of Solid State Chemistry 2022, 310, 123041. https://doi.org/10.1016/j.jssc.2022.123041.

(29) Zhao, Z.; Ren, H.; Shi, Y.; Tan, J.; Xin, X.; Yang, D.; Jiang, Z. Active Site Engineering in Heterovalent Metal Organic Frameworks for Photocatalytic Ammonia Synthesis. Chemical Engineering Journal 2022, 443, 136559. https://doi.org/10.1016/j.cej.2022.136559.
